# EpCAM–PSMA: Potential predictors of treatment outcomes for PSMA-targeted alpha therapies in metastatic castration-resistant prostate cancer

**DOI:** 10.1016/j.omton.2026.201143

**Published:** 2026-02-02

**Authors:** Gábor Bakos, Ulrike Bauder-Wüst, Jonathan Landry, Mareike Roscher, Beáta Ramasz, Frank Bruchertseifer, Alfred Morgenstern, Clemens Kratochwil, Vladimír Beneš, Martina Benešová-Schäfer

**Affiliations:** 1 Research Group Molecular Biology of Systemic Radiotherapy, German Cancer Research Center (DKFZ), 69120 Heidelberg, Germany; 2 Research Group Translational Radiotheranostics, German Cancer Research Center (DKFZ), 69120 Heidelberg, Germany; 3 Genomics Core Facility, European Molecular Biology Laboratory (EMBL), 69117 Heidelberg, Germany; 4 Service Unit for Radiopharmaceuticals and Preclinical Studies, German Cancer Research Center (DKFZ), 69120 Heidelberg, Germany; 5 Flow Cytometry Core Facility, European Molecular Biology Laboratory (EMBL), 69117 Heidelberg, Germany; 6 European Commission, Joint Research Centre (JRC), 76125 Karlsruhe, Germany; 7 Department of Nuclear Medicine, University Hospital Heidelberg, 69120 Heidelberg, Germany

**Keywords:** prostate-specific membrane antigen, PSMA, epithelial cell adhesion molecule, EpCAM, metastatic castration-resistant prostate cancer, mCRPC, targeted alpha therapy, TαT, circulating tumor cells, CTCs, treatment response prediction, surface marker dynamics, liquid biopsy, gene expression analysis, treatment resistance

## Abstract

Targeted radionuclide therapy and targeted alpha therapy directed at prostate-specific membrane antigen (PSMA) represent emerging treatment modalities for metastatic castration-resistant prostate cancer (mCRPC). However, therapeutic resistance remains a significant barrier to their clinical success. We discovered that dynamic changes in cell surface levels of epithelial cell adhesion molecule (EpCAM) and PSMA can serve as predictive biomarkers in late-stage mCRPC patients treated with the beta-minus-particle-emitting [^177^Lu]Lu-PSMA-617, in combination with the alpha-particle-emitting [^225^Ac]Ac-PSMA-617, and we further explored the underlying molecular mechanisms. Using flow cytometry to profile EpCAM and PSMA on circulating tumor cells (CTCs), we observed that Nonresponders displayed significantly higher EpCAM and lower PSMA levels than Responders, both at baseline and after the first treatment cycle. Over subsequent cycles, both markers declined in Nonresponders, whereas Responder CTCs maintained EpCAM expression but progressively lost PSMA. Transcriptome analysis identified upregulation of hub genes involved in the regulation of key pathways such as enhanced DNA-damage repair, anti-apoptotic activity, increased tumor cell growth, and altered surface marker trafficking and recycling, potentially driving EpCAM-PSMA dynamics and contributing to therapy resistance. Ultimately, integrating surface-marker-driven treatment response predictions with novel treatment strategies may help to overcome treatment resistance in mCRPC.

## Graphical abstract

**Figure:**
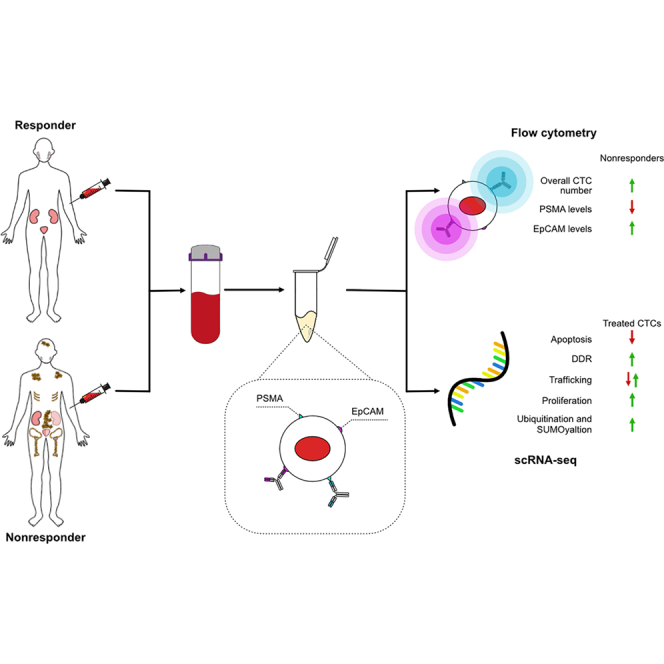

## Teaser

This study examines circulating tumor cells from patients with advanced prostate cancer undergoing PSMA-targeted radionuclide therapy. Dynamic changes in EpCAM and PSMA surface markers reveal therapy-driven phenotypic plasticity indicative of treatment response. These findings underscore the value of CTC profiling for understanding treatment response and resistance in late-stage metastatic disease.

## Introduction

Targeted alpha therapy (TαT) is an emerging systemic treatment modality against prostate-specific membrane antigen (PSMA)-expressing prostate cancer (PCa) and is particularly promising for treating the metastatic forms of the disease.^1^ TαT relies on a two-component drug, also called radiopharmaceutical, consisting of a PSMA-targeting component, and an alpha-particle-emitting radionuclide. These alpha-emitters deliver a highly localized, cytotoxic dose of ionizing radiation directly to cancer cells, minimizing damage to surrounding healthy tissue.^2^ As PSMA is frequently overexpressed on the surface of PCa cells, including those in advanced stages, TαT has shown promising efficacy offering a novel, precision approach to combat aggressive PCa.^3^^,^^4^ This modality is especially valuable for patients with limited treatment options, demonstrating significant potential in improving survival rates and quality of life.

Even though many patients react favorably to TαT, the ever-looming threat of therapy resistance is still present.^5^ In order to develop potential countermeasures against TαT resistance and to efficiently utilize the limited ^225^Ac supplies, an early treatment-response detection and a better understanding of treatment resistance are necessary. Liquid biopsies are known for enabling the detection of early signs of treatment resistance, monitoring tumor progression, and assessing minimal residual disease without the need for repeated, invasive tissue sampling.^6^ This is particularly important in metastatic castration-resistant prostate cancer (mCRPC), where tracking genetic alterations or monitoring of relapse can significantly impact clinical decisions and personalized treatment plans. The ability to detect and analyze circulating tumor cells (CTCs) also allows for better assessment of therapeutic responses, making liquid biopsy a powerful tool for improving precision medicine and hence potentially enhancing patient outcomes.^7^

Therefore, we set out to explore various cell populations based on cell surface marker signals and to identify transcriptional changes in CTCs obtained from mCRPC patients undergoing PSMA-TαT. Our aim was to explore molecular markers, helpful in the early detection of treatment outcomes, and to shed light on potential mechanisms leading or contributing to treatment resistance. In order to carry out this study, we selected patients with late-stage mCRPC currently undergoing a targeted radionuclide therapy (TRNT) combination with [^177^Lu]Lu-PSMA-617 and [^225^Ac]Ac-PSMA-617, for peripheral blood collection (Figure S1). This allowed us to isolate CTCs and analyze changes in the levels of selected cell surface markers—namely epithelial cell adhesion molecule (EpCAM) and PSMA—as well as general transcription levels of various genes upon PSMA-TαT by utilizing flow cytometry and single-cell mRNA sequencing. It also allowed us to compare these changes among responding and non-responding patients, referred to as Responders and Nonresponders, respectively, in the hope of finding molecular markers useful in predicting treatment outcomes and explaining potential mechanisms behind the different treatment response.

## Results

### Patient pre-classification

We set out to collect and store CTC-containing liquid biopsy samples from heavily pre-treated patients undergoing PSMA-TRNT/-TαT using a combination of [^177^Lu]Lu-PSMA-617 and [^225^Ac]Ac-PSMA-617. In order to minimize the potential batch effect and experimental bias during downstream sample processing, we opted to pre-classify the patients before CTC isolation and divide them into two patient groups based on their treatment response: Responders and Nonresponders. The pre-classification was done by a trained and treating physician, and it was based on the trajectory of the patient’s overall prostate-specific antigen (PSA) levels throughout the treatment course (Table S1), combined with positron emission tomography (PET) and/or single photon emission computed tomography (SPECT) imaging data, if available. Although clinical treatment response designation defines more than two outcomes,^8^^,^^9^ we opted to classify both partial (PR) and complete response (CR) as response, while both stable (SD) and progressive disease (PD) as non-response, despite the fact that SD may represent clinical benefit in late-stage disease. Overall, out of the 66 patients (143 total samples), we classified 28 as overall Responders (74 total samples) and 16 as overall Nonresponders (43 total samples). Twenty two patients (28 total samples) were excluded from the analysis, as their response status could not be determined.

### Buffy coat and subsequent CTC isolation

The liquid biopsy samples were collected right before each scheduled treatment cycle, together with the standard blood samples for biomarker testing. The described collection approach was designed to minimize harm to the patients and to avoid the need to process radioactive blood samples. To minimize potential batch effect and experimental bias, we adopted a previously described procedure to freeze CTCs concentrated in the buffy coat layer of whole blood^10^ until downstream processing. To further tailor the protocol to our needs, we adjusted the freezing and thawing procedures using healthy whole blood samples spiked with PSMA^+^EpCAM^+^ C4-2 cells. After optimization, we achieved an over 90% survival rate among C4-2 cells following buffy coat isolation, freezing, and thawing, confirmed by crystal violet staining and subsequent cell counting under light microscope (data not shown).

Next, we set out to isolate the CTCs found in cryopreserved buffy coat samples of each pre-classified patient and to monitor the potential difference in CTC numbers between the response groups. In order to effectively isolate CTCs, we devised a gating strategy involving two exclusion gates to filter out any apoptotic or dead cells, and any potential leukocytes remaining after depletion, and a sort gate that served to capture CTCs expressing two surface markers: EpCAM, a commonly used CTC marker,^11^^,^^12^^,^^13^ and PSMA, a specific marker found on the surface of PCa cells,^14^^,^^15^ which serves as the target for radiopharmaceuticals.^16^^,^^17^^,^^18^ We reasoned that, by using both markers we can maximize sort efficiency by capturing three hypothetical CTC populations: EpCAM^+^PSMA^−^, EpCAM^+^PSMA^+^, and EpCAM^−^PSMA^+^ (Figures S2 and S3). It is important to note that, although EpCAM/PSMA-based staining may underrepresent certain CTC populations at different stages of epithelial-to-mesenchymal transition (EMT),^19^^,^^20^^,^^21^ this strategy was deliberately chosen to interrogate EpCAM/PSMA-expressing CTC subsets that are most relevant to PSMA-TRNT. Indeed, by using the described staining and gating strategy, we isolated a total of 436 CTCs (192 from Responders and 244 from Nonresponders) from 117 samples obtained from 46 patients. As not every sample yielded viable CTCs, the final number of patients (17 Responders, 12 Nonresponders) and samples (74) were reduced after sorting. Therefore, this study is exploratory and not powered for definitive statistical inference. Nonetheless, we observed cells from all three hypothesized subpopulations based on their surface marker status.

### Surface marker profile of isolated CTC populations

To search for overall trends in surface marker status among the response groups and to gain insight into how PSMA-TαT affects the chosen surface markers and the isolated CTC populations, we conducted an in-depth analysis of the obtained FACS data.

**Figure 1:**
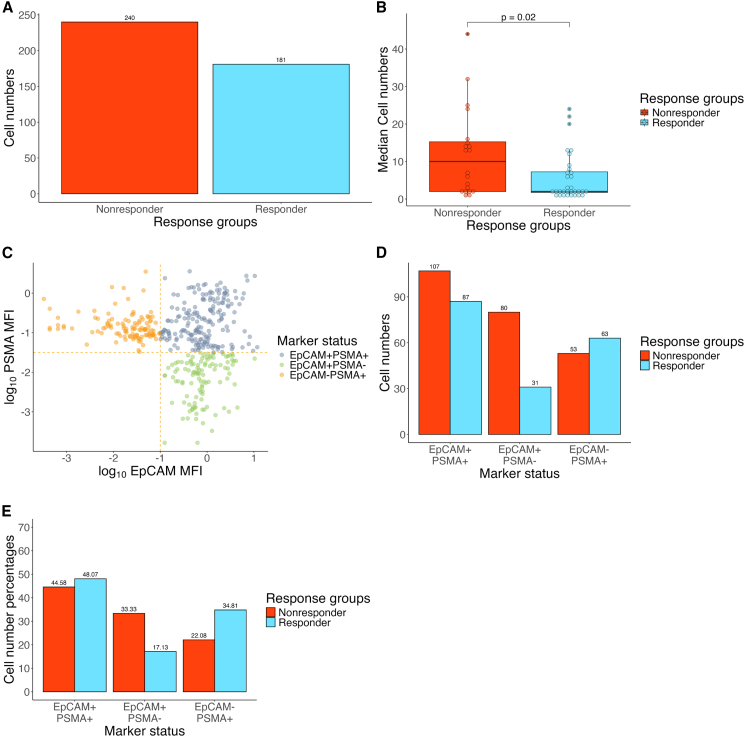
CTC numbers and surface marker distribution (A) A bar chart showing the total sorted CTC cell numbers after data normalization and filtering. (B) A boxplot showing the median sorted CTC numbers per response group. The *p* value (0.02) was calculated using the Mann-Whitney U test. (C) A scatterplot showing the CTC gating strategy and the resulting CTC subpopulations. The yellow dotted line represents the thresholds used to distinguish between various CTC subpopulations, based on their EpCAM and PSMA MFI levels. Note that the sort gate encompassed all three subpopulations. (D) A bar chart showing the distribution of sorted CTCs between the three subpopulations based on EpCAM and PSMA. The chart shows the raw cell numbers in each sub-population. (E) A bar chart showing the distribution of sorted CTCs between the three subpopulations. The chart shows the percentage-based distribution of CTCs from Responder and Nonresponder patients among each sub-population.

First, we performed a general assessment of the obtained cell numbers, to explore potential differences between response groups. Following data normalization and filtering (Figure S4), the final cell number used for data analysis was 422 from a total of 54 samples. We observed a higher total cell number in the Nonresponder samples (*n* = 241) compared to samples from Responders (*n* = 181) (Figure 1A). Additionally, the median sorted CTC number in the Nonresponder group (8, range: 1–44, *n* = 21) was significantly higher (*p* value = 0.0234, Mann-Whitney U test) than in the Responder group (2, range: 1–24, *n* = 33) (Figure 1B). This suggests that Nonresponders have a higher number of CTCs compared to Responders.

Next, we explored how cells from the overall response groups are distributed among the three sorted CTC subpopulations (EpCAM^+^PSMA^−^, EpCAM^−^PSMA^+^, and EPCAM^+^PSMA^+^). To define these subpopulations, we used the minimum median fluorescent intensity (MFI) values to set thresholds on both EpCAM and PSMA channels (Figure 1C). To see, if there are any differences between the distribution of Responder and Nonresponder cells in the various surface marker subpopulations, we looked at both the raw cell counts and the proportional distribution of the two response groups among the subpopulations. We expected to see a close to equal distribution of the two response groups in all three surface marker subpopulations. While the percentage of Responder and Nonresponder cells in the EpCAM^+^PSMA^+^ subpopulation was quite close to equal, we observed a skewed distribution in the EpCAM^+^PSMA^−^ and EpCAM^−^PSMA^+^ subpopulations (Figures 1D and 1E). This suggests that, in general, Nonresponders have a higher number of EpCAM^+^PSMA^−^ CTCs compared to Responders, while Responders have a higher number EpCAM^−^PSMA^+^ CTCs compared to the Nonresponders.

**Figure 2:**
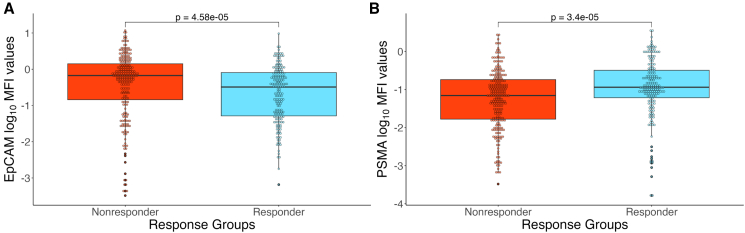
Surface marker comparison between response groups (A) A boxplot showing the comparison of EpCAM MFI values on CTCs sorted from Responder and Nonresponder patients. The *p* value (4.58e−05) was calculated using the Mann-Whitney U test. (B) A boxplot showing the comparison of PSMA MFI values on CTCs sorted from Responder and Nonresponder patients. The *p* value (3.43e−05) was calculated using the Mann-Whitney U test.

This skewed distribution piqued our interest, as this could suggest a generally lower PSMA expression in CTCs found in Nonresponder samples vs. CTCs found in Responder samples. To gain a more granular insight into the differences among the observed cell populations, we further explored the general surface marker levels between Responder and Nonresponder CTCs. Our results showed that the median EpCAM MFI value among the CTCs from Nonresponders (log_10_median-MFI = −0.173, range: −3.36–1.07) was significantly higher (*p* value = 4.58e−05, Mann-Whitney U test) than among CTCs from Responders (log_10_median-MFI = −0.490, range: −3.36–0.983) (Figure 2A). As for the PSMA channel, we observed the opposite, where the median MFI value among the CTCs from the Nonresponders (log_10_median-MFI = −1.160, range: −3.481–0.438), was significantly lower (*p* value = 3.43e−05, Mann-Whitney U test) than among CTCs from Responders (log_10_median-MFI = −0.940, range: −3.297–0.553) (Figure 2B). These data reinforce our previous observation and suggests that CTCs sorted from Nonresponders have higher EpCAM and lower PSMA surface levels than CTCs sorted from Responders.

### Dynamic surface marker changes in various CTC populations

Aggregate MFI values provided valuable information regarding overall differences in EpCAM and PSMA surface levels between Responder and Nonresponder CTC populations. However, because these values were pooled across all treatment cycles, they lacked the temporal resolution necessary to assess dynamic, longitudinal changes in surface marker expression during therapy. Therefore, to investigate potential treatment-associated dynamics in EpCAM and PSMA surface levels, we performed a cycle-to-cycle analysis of median MFI values both within and between response groups.

**Figure 3:**
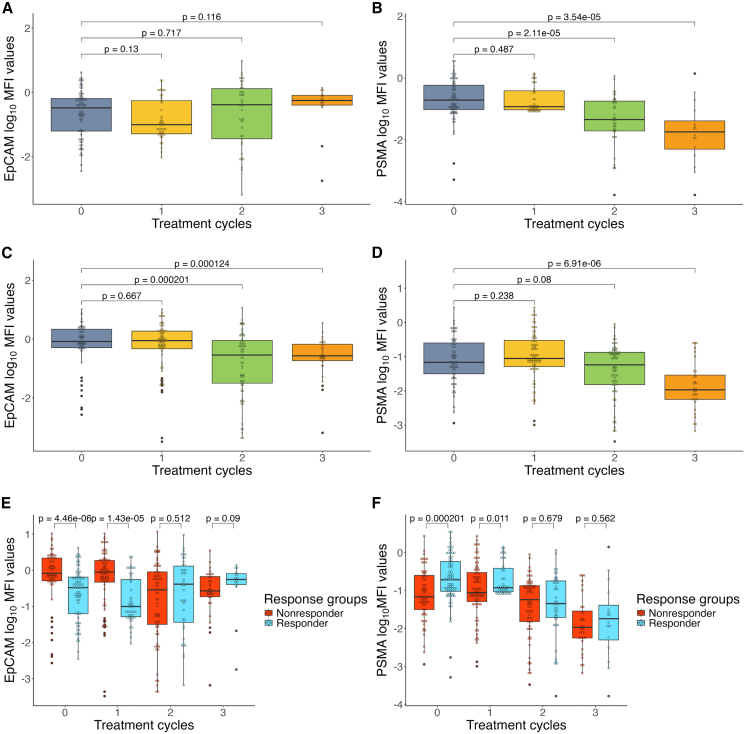
EpCAM and PSMA surface level over the treatment cycles (A) A boxplot showing the changes in EpCAM surface marker levels on CTCs from Responder patients, from cycle 0 to cycle 3. The *p*-values were calculated using the Mann-Whitney-U-test with a significance level of *p* < 0.05 for this and all subsequent panels. (B) A boxplot showing the changes in PSMA surface marker levels on CTCs from Responder patients, from cycle 0 to cycle 3. (C) A boxplot showing the changes in EpCAM surface marker levels on CTCs from Nonresponder patients, from cycle 0 to cycle 3. (D) A boxplot showing the changes in PSMA surface marker levels on CTCs from Nonresponder patients, from cycle 0 to cycle 3. (E) A boxplot showing the changes in EpCAM surface marker levels on CTCs from both Responder and Nonresponder patients, from cycle 0 to cycle 3. *p* values represent the difference in EpCAM surface marker levels on CTCs from the two response groups in any given cycle. (F) A boxplot showing the changes in PSMA surface marker levels on CTCs from both Responder and Nonresponder patients, from cycle 0 to cycle 3. *p* values represent the difference in EpCAM surface marker levels on CTCs from the two response groups in any given cycle.

To this end, first we looked at the number of sorted CTCs per treatment cycle for both Responder and Nonresponder samples. As cycle 4 had a low number of sorted cells (Responders: 3; Nonresponders: 0), we made a cutoff at the 3^rd^ treatment cycle for both response groups. Then we compared the log-transformed MFI values of CTCs from each treatment cycle to the 0-time point (baseline cycle, blood collected shortly before the first PSMA-TαT cycle was administered), to see if there were any changes in the EpCAM and PSMA levels throughout the treatment. In the case of CTCs sorted from Responder samples, we observed a relatively flat trajectory with no significant difference in the EpCAM MFI values in cycles 1–3, compared to the 0-time point (Figure 3A). In the case of PSMA, the results showed a decreasing trajectory with a significant decrease in the median MFI values at cycle 2 (0-time-point median MFI: 0.1920; cycle 2 median MFI: 0.0453; *p* value = 2.11e−05, Mann-Whitney U test) and cycle 3 (0-time-point median MFI: 0.1920; cycle 3 median MFI: 0.0186; *p* value = 3.54e−05, Mann-Whitney U test) compared to the 0-time point (Figure 3B). As for CTCs sorted from Nonresponder samples, the EpCAM MFI values show a declining trend, with a significant decrease at cycle 2 (0-time-point median MFI: 0.8258; cycle 2 median MFI: 0.2872; *p* value = 0.0002, Mann-Whitney U test) and at cycle 3 (0-time-point median MFI: 0.8258; cycle 3 median MFI: 0.2706; *p* value = 0.0001, Mann-Whitney U test) compared to the 0-time point (Figure 3C). In the case of PSMA, we also observed a decline over time with a significant decrease in median MFI values at cycle 3 (*p* value = 6.91e−06, Mann-Whitney U test) compared to the 0-time point (Figure 3D). When compared to CTCs from Nonresponders, the median EpCAM MFI values were significantly lower on CTCs from Responders at 0-time point (Nonresponder-median-MFI: 0.8258; Responder median MFI: 0.3299; *p* value = 4.46e−06, Mann-Whitney U test) and at cycle 1 (Nonresponder-median-MFI: 0.8849; Responder-median-MFI: 0.0992; *p* value = 1.43e−05, Mann-Whitney U test). However, this difference disappeared at later cycles (Figure 3E). In the case of PSMA, although CTCs from both Responder and Nonresponder samples show the same declining trend, cells from the Nonresponder samples show a significantly lower median MFI value at 0-time point (Nonresponder-median-MFI: 0.0683; Responder-median-MFI: 0.1920; *p* value = 0.0002, Mann-Whitney U test) and at cycle 1 (Nonresponder-median-MFI: 0.0883; Responder-median-MFI: 0.1181; *p* value = 0.0110, Mann-Whitney U test) compared to cells from Responder samples. Together, these results suggest that CTCs from Responder samples start with relatively lower EpCAM levels than their Nonresponder counterparts, which stays mostly stable throughout the treatment. Cells form Nonresponders seem to have higher levels of EpCAM at baseline, which shows a decrease over the treatment, most prominently after the first treatment cycle. As for PSMA, cells from Responder samples seem to possess higher levels at baseline; however, this quickly decreases throughout the treatment. A similar decrease in surface PSMA levels can be observed in the cells from Nonresponder samples, in addition to their lower baseline PSMA. The progressive loss of PSMA expression in Responder CTCs may reflect therapy-induced antigen modulation or selective elimination of PSMA-high clones, a phenomenon previously reported for TRNT.

### Exploring the CTC transcriptome: Library preparation and data filtering

The observed dynamic changes in the surface marker levels appeared to have a good potential to predict treatment outcomes. Therefore, we opted to explore molecular mechanisms that underpin both the observed EpCAM-PSMA dynamics and treatment resistance. To this end, we examined the transcriptome of the sorted CTCs by single-cell RNA sequencing (scRNA-seq).

As a first step, we prepared amplified cDNA libraries from the sorted cells, using a Smart-seq2-based approach.^22^ During the library preparation, we noticed that many of the cells produced low-concentration, low-quality, partially degraded RNA. Therefore, we conducted rigorous quality checks by running the cDNA from every cell—following both reverse transcription and tagmentation—on a fragment analyzer. Out of the 436 sorted cells, 31.6% (138 cells total; Responders: baseline, 36 cells; cycle 1, 31 cells; cycle 2, 11 cells; Nonresponders: baseline, 15 cells; cycle 1, 38 cells; cycle 2, 7 cells) passed the quality controls and were moved on to sequencing (Figure S5).

Next, we set out to prepare the resulting sequencing data for the downstream analysis. After filtering, we were left with 99 cells in total (41 cells from Nonresponder and 58 cells from Responder samples) (Figure S6A). In these cells, the median number of detected transcripts was 4,018,895 (range: 4,439–17,336,862) and the median number of detected genes was 3,326 (range: 1,013–7,314) while the median mitochondrial (MT) gene expression percentage was 16.4% (range: 0.1–74.6) (Figures S6B–S6E).

### Exploring the CTC transcriptome: Clustering and gene expression analysis

**Figure 4:**
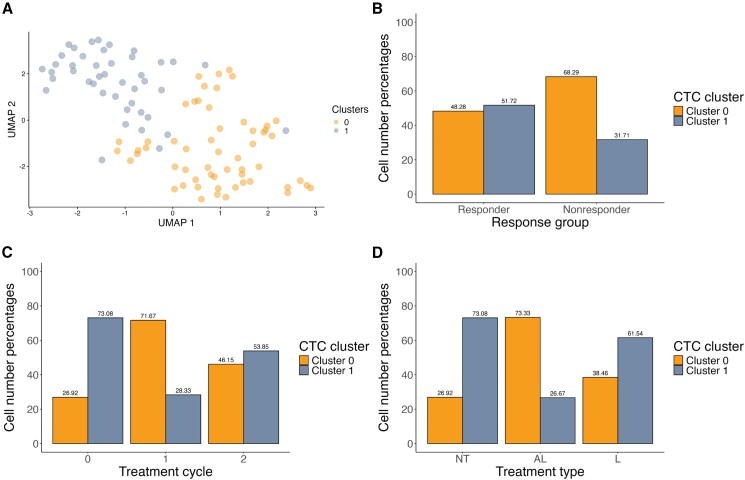
CTC clusters and their correlation to various treatment variables (A) A uniform manifold approximation and projection (UMAP) plot showing the clustering of CTCs based on their gene expression pattern. (B) A bar chart showing the distribution of CTCs between clusters 0 and 1, based on patient response status. (C) A bar chart showing the distribution of CTCs between clusters 0 and 1 based on the treatment cycles they originate from. (D) A bar chart showing the distribution of CTCs between clusters 0 and 1 based on the treatment type the patient received. NT = no treatment, AL = [^225^Ac]Ac-PSMA-617-[^177^Lu]Lu-PSMA-617, L = [^177^Lu]Lu-PSMA-617.

Next, we set out to differentiate various CTC populations by clustering analysis. We performed unsupervised clustering analysis, then visualized the results by a uniform manifold approximation and projection (UMAP) plot (Figure 4A). UMAP clustering showed two distinct clusters among the analyzed CTCs.

To determine if the observed clustering behavior was dependent on the overall treatment response, we explored the relationship between the clusters and response groups (Figure 4B). The clustering behavior showed no significant dependence on the overall treatment response (*p* value = 0.0761, chi-squared test of independence). Therefore, we looked at how other variables—such as the treatment cycle, the cell-cycle phase, treatment type, and surface marker status—influence the clustering behavior of CTCs. Out of these tested variables, treatment cycle and with that, overall treatment status (*p* value = 0.0004, chi-squared test of independence) and treatment type (*p* value = 0.0356, chi-squared test of independence) showed a significant influence on the clustering (Figures 4C, 4D, and S7A–S7C). Overall, this suggests that cluster 0 is mostly composed of cells subjected to treatment with [^225^Ac]Ac-PSMA-617 alongside [^177^Lu]Lu-PSMA-617, collected during treatment cycles 1 or 2. In contrast, cluster 1 is mostly composed of cells not subjected to treatment—collected before the first treatment cycle (baseline)—or exposed to treatment with [^177^Lu]Lu-PSMA-617—collected before the patient received [^225^Ac]Ac-PSMA-617 alongside [^177^Lu]Lu-PSMA-617.

**Figure 5:**
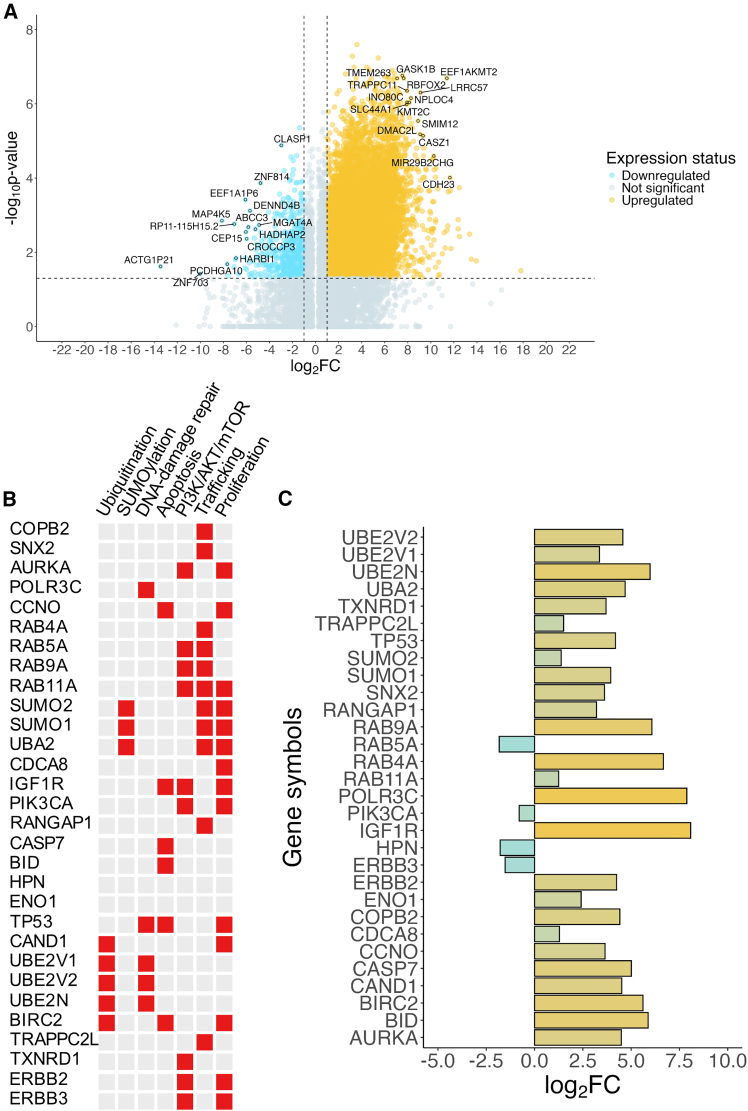
Differential gene expression and selected hub genes (A) A volcano plot showing the differentially expressed genes (DEGs) between cluster 0 vs. cluster 1. The top 15 DEGs based on ranking (log_2_FC ∗ *p*-adjusted value) are highlighted with gene symbols. (B) A heatmap showing the processes the selected hub genes are directly involved in. (C) A bar chart showing the relative expression level of the selected hub genes in cluster 0 vs. cluster 1.

After obtaining some insight about the composition of the clusters, we opted to explore the potential gene expression differences, most likely attributing to the combination treatment with [^225^Ac]Ac-PSMA-617. To this end, we conducted a differential gene expression (DGE) analysis, which identified 8,544 genes as significantly differentially expressed between the two clusters with a *p*-adjusted value of <0.05 (Bonferroni correction) (Table S2). To refine our results, we implemented further filtering based on expression level (log2FC), selecting only those genes with at least a 2-fold difference in expression (log2FC ≤ −1 or log2FC ≥ 1). After the applied filtering, we were left with a total of 7,630 significantly differentially expressed genes (DEGs). Out of these, 7,176 genes were upregulated in cluster 0 vs. cluster 1, while 454 genes were downregulated in cluster 0 compared to cluster 1 (Figure 5A).

To further explore the processes and pathways these DEGs are involved in, we followed-up the DEG analysis with an integrated gene set enrichment analysis (irGSEA).^23^ Using the MSigDB database as a reference to explore impacted pathways, we ran the analysis against multiple reference gene sets containing both higher level (Hallmark, Biocarta, and GO:Biological) and more detailed pathway information (Processes and REACTOME). The results showed that multiple key cellular processes are differentially regulated between the two clusters, including DNA-damage repair (DDR), apoptosis, cellular trafficking, and cell growth (Figures S8 and S9).

To elucidate how these pathways are impacted by the treatment, we opted to explore the key associated genes and their expression status in the clusters. We conducted a hub gene analysis using the irGSEA.hub function, which after thresholding identified 30 hub genes (Table S3), directly involved in ubiquitination, SUMOylation, cellular trafficking, and the regulation of the PI3K/AKT/mTORC pathway. Most of the hub genes were upregulated in cluster 0 compared to cluster 1, with a few notable exceptions (RAB5A, PIK3CA, HPN, and ERBB3) (Figures 5B and 5C). Taken together, the results indicate that CTCs from later treatment cycles show enhanced DDR and reduced apoptosis. Furthermore, they seem to have decreased clathrin-dependent endocytosis and perturbed anterograde and retrograde trafficking. Finally, they seem to exhibit increased SUMOylation and a positive modulation of the PI3K/AKT/mTORC pathway.

## Discussion

Since PCa incidence is projected to rise substantially in high-income countries over the coming decades,^24^ the importance of efficient treatment modalities and predictive diagnostic tools will increase in the coming years. PSMA-TαT shows promise as an effective treatment option for late- and potentially early-stage mCRPC patients, who have developed resistance to other therapies, offering a median overall survival of 15 months.^25^ However, therapy resistance remains a significant challenge, often resulting in poor treatment outcomes. To identify markers capable of predicting treatment outcomes and uncover mechanisms that might lead to or contribute to therapy resistance, we collected samples from a limited number of patients with diverse pre-treatment backgrounds who underwent combined [^225^Ac]Ac-PSMA-617 and [^117^Lu]Lu-PSMA-617 therapy (Table S6). Although this increases the complexity of the obtained data, we believe it is important to reflect the current clinical reality and explore the potential resistance mechanisms under frequently used treatment schedules and pre-treatment conditions. The obtained samples were processed by flow cytometry and single-cell RNA sequencing.

Single-cell sorting data revealed significant differences in EpCAM and PSMA surface levels on CTCs from Responders versus Nonresponders. Specifically, CTCs from Nonresponders showed a significantly higher median EpCAM and a significantly lower median PSMA level than CTCs from Responders (Figures 2A and 2B). Furthermore, by analyzing marker levels across treatment cycles, we found that this difference was already present before treatment initiation (Figures 3E and 3F). During treatment, surface levels of both EpCAM and PSMA declined in Nonresponder CTCs, whereas Responder CTCs showed relatively stable EpCAM and decreasing PSMA levels (Figures 3A–3D). Although it would stand to reason that this decline is connected to the downregulation of these markers, differential gene expression analysis revealed no significant differences between the response groups. This indicates that the surface levels of these markers are regulated at the translational or posttranslational, rather than the transcription, level. Interestingly, the differences in cell surface markers between the two response groups diminished over the course of treatment and disappeared by later cycles (Figures 3E and 3F). This suggests that treatment with [^225^Ac]Ac-PSMA-617 induces a similar adaptation or selection process in CTCs from both response groups. This means that as the treatment progresses, CTCs with higher PSMA and lower EpCAM levels are effectively eliminated in both patient groups. Therefore, we end up isolating the surviving cells that might display similar gene expression patterns. This notion is supported by the obtained scRNA-seq data, where the clusters obtained by unsupervised clustering analysis (Figure 4A) correlated primarily with the treatment status (non-treated, cycle 0; treated, cycle 1–2) and treatment type ([^177^Lu]Lu-PSMA-617 alone or [^177^Lu]Lu-PSMA-617 combined with [^225^Ac]Ac-PSMA-617). Notably, these clusters were less influenced by other parameters such as overall response status (Figures 4B–4D; Figure S6). Nonetheless the combination of lower PSMA and higher EpCAM levels on CTCs, along with increased detected CTC numbers, appears to correlate with treatment resistance. This observation may offer a valuable complementary diagnostic tool for predicting treatment outcomes either before (baseline) or during early treatment cycles (cycles 1 and 2). Given the fact that the observed EpCAM-PSMA dynamics was detected using flow cytometry, our depletion and staining protocol combined with the existing or potentially improved analysis script can offer an easy to adopt method for clinical laboratories. Such tools could be particularly relevant in patient stratification if [^225^Ac]Ac-PSMA-617 therapy becomes widely adopted—especially as a third- or second-line treatment option—given current constraints in the ^225^Ac supply.

As for the molecular pathways potentially underpinning both the observed EpCAM-PSMA dynamics and potential treatment resistance, transcriptome analysis revealed significant changes in the expression of various hub genes directly involved in ubiquitination, SUMOylation, DDR, regulation of the PI3K/AKT/mTOR pathway, proliferation control, and most importantly, cellular trafficking (Figure 5B; Table S3). Most of these hub genes were upregulated in response to [^225^Ac]Ac-PSMA-617 treatment apart from a few notable exceptions (RAB5A, PIK3CA, HPN, and ERBB3), prompting the modulation of key survival processes.

Among the hub genes upregulated upon PSMA-TαT, several may explain the observed surface marker dynamics and highlight mechanisms leading to treatment resistance. For example, we identified upregulated genes such as UBE2N, UBE2V1, and UBE2V2, ubiquitin-conjugating enzymes essential for DSB repair,^26^^,^^27^^,^^28^^,^^29^ as well as POLR3C, a subunit of RNA polymerase III involved in homologous recombination repair of DSBs.^30^ This represents a key survival response to the DNA damage induced by alpha radiation. Upregulation of anti-apoptotic regulators further highlights the adaptive survival mechanisms in TαT-treated CTCs. BIRC2, an inhibitor of apoptosis involved in the ubiquitination and degradation of caspase-3 and -7,^31^ and IGF1R, which supports cell survival by activating the AKT pathway,^32^^,^^33^ were upregulated upon treatment. Significant alterations in cellular trafficking pathways were also observed in TαT-treated CTCs, with the upregulation of key regulators such as COPB2. COPB2, a member of the COPI complex and an important regulator of Golgi-to-ER retrograde trafficking, regulates the nuclear trafficking of receptor tyrosine kinases (RTKs) such as ERBB2.^34^ Similarly, members of the RAB family, which is crucial for intracellular trafficking, were also upregulated. RAB4A, located in early and recycling endosomes, promotes the rapid recycling of integrins, EGFR, and other surface proteins, enhancing proliferation, invasiveness, and metastasis formation.^35^^,^^36^^,^^37^^,^^38^ RAB11A, a key player in the slow recycling pathway, may disrupt normal trafficking of internalized PSMA (FOLH1), increasing retention in recycling endosomes (RE). This mirrors the behavior observed for transferrin,^38^^,^^39^ which shares remarkably similar trafficking dynamics with PSMA.^40^ The upregulation of TRAPPC2L, a TRAPPII/III complex adaptor with guanosine exchange factor (GEF) activity, may further enhance RAB11 activity and increase COPI and COPII vesicle trafficking, thereby affecting ER-to-Golgi and Golgi-to-ER transport.^41^^,^^42^

Conversely, we also identified hub genes with reduced expression upon PSMA-TαT. Among these was RAB5A, another member of the RAB family found in early endosomes. RAB5A is primarily responsible for cargo sequestration and participates in the clathrin-mediated endocytosis of transferrin and G-protein-coupled receptors.^37^^,^^43^ Its downregulation aligns with previous observations in advanced PCa.^44^ Similarly, PIK3CA, the catalytic subunit of phosphoinositide-3-kinase (PI3K), was downregulated. This gene is commonly overexpressed and reported to more frequently harbor activating rather than non-activating mutations in PCa.^45^^,^^46^ It plays a role in activating the PI3K/AKT/mTOR pathway, which promotes cell survival, growth and cell-cycle progression, and is upstream of AKT.^47^ Notably, AKT can still be activated independently of PI3K by various kinases such as TBK1, found to be upregulated in cells from cluster 0.^47^^,^^48^^,^^49^ Lastly, ERBB3, an RTK connected to PI3K/AKT and AR signaling in PCa,^50^ was also downregulated. While ERBB3 typically requires ERBB2 as a dimerization partner for AKT activation, ERBB2 alone can activate the PI3K/AKT pathway when overexpressed.^51^

Overall, these transcriptional changes may represent key survival and adaptation mechanisms in CTCs. They include enhanced DSB repair activity, suppression of apoptotic pathways, and modulation of the trafficking and surface levels of critical proteins such as PSMA, ERBB2, and IGF1R.

Based on these findings, we propose a model to explain the observed surface marker dynamics on CTCs—and potentially on solid metastatic sites—as well as the mechanisms that may contribute to treatment resistance in patients with advanced mCRPC. In this model, mCRPC patients harbor multiple populations of CTCs and likely solid metastatic cells as CTCs stem from solid tumor sites. These populations can be distinguished by a detectable difference in their surface EpCAM and PSMA levels likely linked to the differential expression of genes responsible for cellular trafficking (e.g., COPB2, TRAPPC2L, SUMO1, SUMO2, and UBA2) and recycling (e.g., RAB4A and RAB11A). Additionally, differences in surface marker expression are accompanied by a distinct gene expression pattern, termed the “Cluster 0” (C0) pattern, which promotes CTC survival. This pattern is characterized by increased DSB repair activity (e.g., UBE2N, UBE2V1, UBE2V2, and POLR3C), elevated anti-apoptotic activity (e.g., BIRC2, IGF1R, and CCNO), and enhanced growth and proliferation, driven primarily by the PI3K/AKT/mTOR pathway. Therefore, CTCs and potentially other tumor cells with lower PSMA surface levels tend to be targeted less effectively by [^225^Ac]Ac-PSMA-617, resist damage more efficiently, and retain the ability to proliferate even under therapeutic pressure. Over time, this results in the selection and enrichment of such CTCs—and potentially PSMA-low solid tumor cells—in treated patients, ultimately leading to reduced treatment efficacy and resistance. Our model suggests that patients who develop treatment resistance earlier possess a larger population of PSMA-low, EpCAM-high, C0-pattern CTCs and potentially solid tumor cells, even before [^225^Ac]Ac-PSMA-617 treatment begins. This pre-existing cellular heterogeneity could explain the observed reduction in treatment efficacy and poorer clinical outcomes in resistant cases. As the PSMA-low, EpCAM-high, C0-pattern CTC population is not unique to Nonresponders, early detection of treatment resistance is extremely important, as it can offer physicians the time to develop alternative treatment plans and regimes such as the use of combination therapies, using DDR inhibitors, RTK inhibitors, and AKT inhibitors.

**Figure 6:**
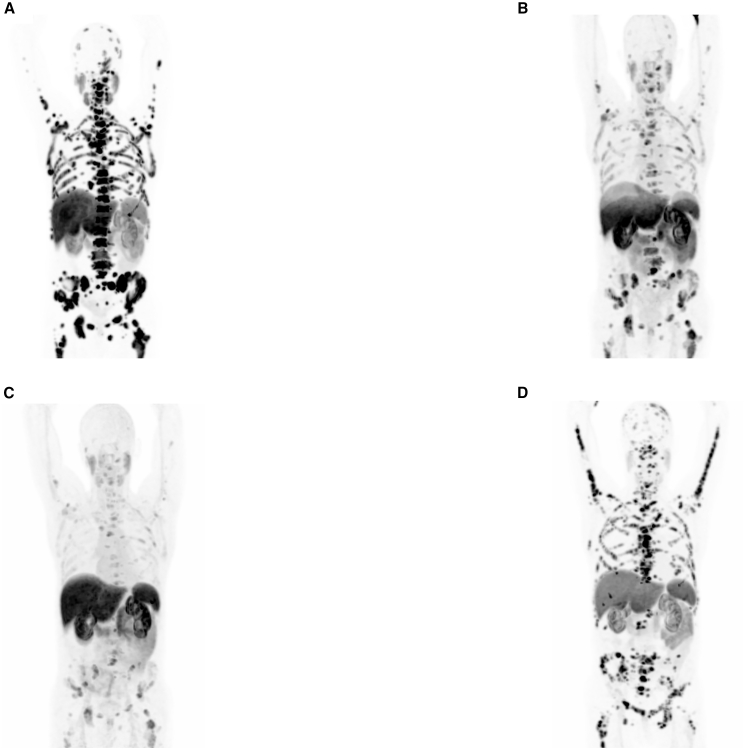
[^68^Ga]Ga-PSMA-PET scans of a Responder patient (ALM60C8) (A) A scan showing solid tumor sites before the combined treatment with [^225^Ac]Ac-PSMA-617-[^177^Lu]Lu-PSMA-617 (cycle 0). (B) A scan showing solid tumor sites after the 3rd treatment cycle. (C) A follow-up scan ∼3 months after the last recorded treatment cycle. (D) A scan showing the resurgence of the tumor 2 years following the last recorded treatment cycle. The relapsed patient showed resistance to continued [^225^Ac]Ac-PSMA-617-[^177^Lu]Lu-PSMA-617 treatment even though the metastatic sites are well visible on the [^68^Ga]Ga-PSMA-PET scan.

Nonetheless, it is important to note that our model is based on data obtained from CTCs, making it challenging to extrapolate findings to solid tumor sites due to their vastly different microenvironments. This might also help to explain the discrepancy with previously reported clinical observations,^5^ where resistant patients show strong PSMA expression in solid tumor sites even after multiple treatment cycles (Figure 6). While the relationship between PSMA levels expressed on the tumor cells necessary for diagnostic imaging and those sufficient for effective therapeutic targeting remains poorly understood, it is likely that additional resistance mechanisms independent of targeting evasion are at play and warrant further investigation. Moreover, we cannot exclude preferential loss of fragile CTC subsets, although this limitation applies uniformly across the cohort.

Ultimately, our results suggest the presence of various CTC populations based on their surface EpCAM and PSMA levels, in mCRPC patients. Among these populations, we identified two, which seems to correspond well with the development of resistance to [^225^Ac]Ac-PSMA-617. The presence of EpCAM-high and PSMA-low population seems to be dominant in patients with poor therapy response, while the presence of EpCAM-low and PSMA-high population seems to be dominant in patients with favorable therapy response even before the first round of treatment. These observations, although from a limited cohort, suggests that the observed EpCAM-PSMA dynamics could be used as a powerful complementary diagnostic tool, in predicting resistance to PSMA-TαT. This gives us an exciting opportunity to further explore these dynamics and develop robust prediction algorithms using large patient cohorts.

## Materials and methods

### Patient selection and treatment

[^225^Ac]Ac-PSMA-617 and [^177^Lu]Lu-PSMA-617 were prepared for injection as described before^52^^,^^53^ and administered as a compassionate use in adherence to paragraph 37, “Unproven Interventions in Clinical Practice,” of the revised Declaration of Helsinki. The administered activities ranged from 2 to 8 MBq for [^225^Ac]Ac-PSMA-617 and 2 to 8.5 GBq for [^177^Lu]Lu-PSMA-617. In total, 46 heavily pre-treated patients with age ranging from 57 to 88 years (74.3 years average and 75.5 years median) were enrolled for the study. All patients had a high metastatic burden despite receiving multiple lines of prior systemic therapy, including at least one novel androgen-receptor-targeted therapy (e.g., abiraterone or enzalutamide) and/or prior chemotherapy (e.g., docetaxel), and had shown either disease progression or discontinued treatment due to adverse effects. Given the heavily pre-treated nature of the cohort, baseline EpCAM expression reflects therapy-adapted disease rather than early-stage mCRPC biology. Enrollment in the study is contingent upon obtaining written informed consent from patients under ethical approval given by the ethical committee of University Hospital Heidelberg (S-882/2020).

### Cell culture

Cell lines were cultured according to standard mammalian tissue culture protocol and sterile technique at 37°C in 5% CO_2_. C4-2 and PC-3 cells were cultured in RPMI-1640 media, supplemented with 10% FCS and 1% stable l-glutamine. Routine cell culture was performed twice a week using a room-tempered PBS (pH 7.4) for wash and 0.05% trypsin for cell detachment.

### Blood collection

Both patient and healthy control blood was collected in order to isolate CTCs or to optimize the isolating procedure.

Briefly, 2× 9 mL full blood was drawn from patients if health conditions allowed, into 10 mL Vacutainer tubes (BD Biosciences, Franklin Lakes, New Jersey, USA). The collected blood samples were placed on 4°C for 2–4 h until collection. Collected samples were transferred to ice until further buffy coat isolation. In the case of healthy blood used for process optimization, the samples were spiked with 1^2^–10^6^ C4-2 cells before buffy coat isolation.

### Buffy coat isolation and freezing

In order to isolate and preserve CTCs for fluorescent-activated cell sorting, the buffy coat layers of full blood samples were isolated by taking the following steps:

Samples were centrifuged at 800×*g* for 10 min on 4°C, without brake. Following centrifugation, both the plasma and the buffy coat were carefully transferred into 15 mL tubes. Tubes containing plasma were stored on ice until the end of the procedure, then transferred into −80°C for storage.

Tubes containing the buffy coat were supplied with 5× sample volume TheraPEAK ACK lysis buffer (Lonza, Basel, Switzerland, cat # BP10-548E), then gently mixed and incubated for 5 min at room temperature. Following incubation, the samples were centrifuged at 200×*g* for 5 min, then the lysis was repeated once more.

Following complete lysis, the samples were centrifuged at 200×*g* for 5 min and washed with 10 mL of PBS.

After washing, the samples were re-suspended in 500 μL RPMI-1640 media supplemented with 10% FCS. The samples were supplemented with 2.5 nM MgCl_2_ (Sigma-Aldrich, St. Louis, USA, CAS # 7786-30-3) and 120 IU (32.4 μM) DNAse I (Roche, Basel, Switzerland, cat # 11284932001) and incubated on ice for 30 min. Following incubation, the samples were mixed with an equivalent volume of FCS supplemented with 20% V/V DMSO (Sigma-Aldrich, St. Louis, USA, cat #D2650-100ML) by dropwise mixing.

The mixes were transferred into Mr. Frosty (Nalgene Nunc, Thermo Fisher Scientific, Rochester, USA) freezing containers and placed on −80°C. For storage longer than 3 months, the samples were moved from −80°C to liquid nitrogen.

All samples underwent identical cryopreservation and thawing procedures, minimizing systematic bias between response groups.

### Buffy coat processing and CTC staining

In order to set up buffy coat thawing and CTC sorting conditions, C4-2-spiked healthy blood samples were used. Based on these results, further optimization was carried out using real patient samples, which resulted in the finalized procedure consisting of the following main steps.

#### Buffy coat thawing and surface-marker staining

Frozen buffy coat samples from −80°C or liquid nitrogen were thawed rapidly in a 37°C water bath. To avoid batch effect, Responder and Nonresponder samples were randomly paired, and a two-step isolation process was carried out on the paired buffy coat samples after thawing.

Samples were transferred into 30 mL warm RPMI-1640 media supplemented with 10% FCS, 1 μM Tirofiban Hydrochloride (Sigma-Aldrich, St. Louis, USA, cat # 150915-40-5), 2.5 mM MgCl_2_, and 600 IU (2.7 μM) DNAse I.

The samples were incubated at room temperature for 30 min to reduce cell aggregation by removing free DNA and inhibiting glycoprotein IIb/IIIa.

Following the incubation, the samples were centrifuged at 200×*g* for 5 min on room temperature, then re-suspended in 200 μL anti-CD45 antibody mix containing 2.5 ng/μL anti-CD45-biotin (eBioscience, San Diego, USA, HI30 cat # 13-0459-82), 2.5 nM MgCl_2_, and 2 U (27 nM) DNAse I. The samples were placed on ice and incubated for 45 min.

The samples were washed with 1 mL PBS supplemented with 2% FCS (PBS-FCS), then centrifuged on 200×*g* for 5 min.

Following centrifugation, the supernatant was carefully discarded, and the pellet was re-suspended in 240 μL PBS-FCS supplemented with 2.5 nM MgCl_2_, 2.4 U (27 nM) DNAse I, and 30 μL anti-biotin MicroBeads (Miltenyi, Bergisch Gladbach, Germany, cat # 130-090-485).

The re-suspended samples were incubated on ice for 20 min, then washed with 1 mL PBS-FCS.

#### Leukocyte depletion, apoptotic staining, and live-dead staining

As the thawed buffy coat is mainly consisting of leukocytes while the CTCs represented an extremely low fraction, we carried out a leukocyte depletion step before cell sorting using a combination of anti-CD45-biotin antibody and magnetic streptavidin beads.

The samples were centrifuged at 200×*g* for 5 min, re-suspended in 500 μL PBS-FCS, and transferred onto LS magnetic separator columns (Miltenyi, Bergisch Gladbach, Germany, cat # 130-042-401) where the CD45-depleted fraction was collected as flow-through.

Following separation, the columns were washed 4× with 2 mL PBS-FCS, and the resulting flow-through was pooled with the CD45-depleted fraction.

Following washing, the columns were removed from the magnetic stand, flushed out with 500 μL PBS-FCS to collect the CD45^+^ cell containing fraction for further use. This step resulted in a significant reduction in overall cell numbers and allowed us to reduce cell-sort times and preserve CTC viability during the last step of the isolation process.

Next, the CD45-depleted samples were centrifuged at 200×*g* for 5 min, then the supernatant was carefully discarded, and the remaining cells were re-suspended in 160 μL staining-mix containing 1.25 ng/μL Brilliant Violet 421 Streptavidin (BioLegend, San Diego, USA, cat # 405226), Alexa Fluor 488 Anti-EpCAM antibody [323/A3] (Abcam, Cambridge, UK cat # ab253268) in 1:50× dilution, and anti-PSMA antibody REA408—PE-Vio 770 (Miltenyi, Bergisch Gladbach, Germany, cat # 130-131-264) in 1:160× dilution.

The samples were incubated on ice for 60 min, then washed with 1 mL PBS-FCS, centrifuged at 200×*g* for 5 min and re-suspended in 300 μL PBS-FCS, supplemented with 120 nM DAPI (Sigma-Aldrich, St. Louis, USA, cat #D9542-10MG) and 166 nM Incucyte Caspase-3/7 Red Dye (Sartorius, Göttingen, Germany, cat # 4704).

The samples were kept on ice for 15–30 min, which was followed by single-cell sorting on a cell sorter.

### Flow cytometry: Single-cell sorting

Stained cell suspensions were sorted using a BD FACSAria Fusion Flow Cytometer using either a 100 μm or 130 μm nozzle (no impact on post-sort quality) with standard BD FACSFlow sheath fluid. The instrument is equipped with five lasers (355 nm UV, 405 nm violet, 488 nm blue, 561 nm yellow-green, and 633 nm red), and further details regarding the optical configuration can be found in Table S5.

Single cells were index-sorted directly into 4.4 μL lysis buffer (0.4% Triton X-100, 2 U/μL RNasin Plus, 1 μM Oligo-dT30VN, 2 μM dNTP) in 96-well plates to enable downstream correlation of EpCAM and PSMA fluorescence intensity with scRNA-seq data. To ensure accuracy and consistency in gating, three types of control samples were routinely used at the beginning of each sorting experiment (Figures S3B–S3D). These included (1) a negative control without antibody staining, (2) a positive control consisting of C4-2 PCa cells processed and stained identically to patient samples, and (3) a CD45^+^ leukocyte population isolated during the CD45 depletion step for circulating tumor cell (CTC) enrichment, also stained with the same antibody cocktail. The consistent use of all three control samples ensured reliable and reproducible gating across all experiments. The employed gating hierarchy is illustrated in the Supplemental Material (Figure S2F). Following sorting, the plates were stored at −80°C.

### Sequencing library preparation

Cells sorted into lysis buffer were processed using a modified version of the SmartSeq2 protocol, based on the works by Picelli et al., 2014^22^ and Hennig et al., 2018.^54^ As RNA degradation during sample processing cannot be excluded, the Smart-Seq2 method was selected for its robustness in low-input single-cell applications.

#### Reverse transcription

Samples were thawed on ice after being removed from −80°C and placed into a thermocycler, where they were heat-lysed by incubating at 72°C for 3 min.

The lysed samples were then cooled on ice for at least 2 min before being supplied with 6.35 μL of reverse transcription (RT) master mix per sample. For a single sample, the RT master mix contained 2 μL SSRT IV 5× buffer (Invitrogen/Thermo Fisher Scientific, Waltham, USA, cat # 18091050), 0.5 μL 100 mM DTT, 2 μL 5M betaine, 0.1 μL 1M MgCl_2_, 0.25 μL RNAse inhibitor (40 U/μL, Clontech Takara, Kusatsu, Japan, cat # 2313A), 0.25 μL SSRT IV (Invitrogen/Thermo, Waltham, USA, cat # 18091050), and 0.1 μL 100 μM Template Switching Oligo (TSO; sequence in Table S7).

The mixture was then pulse-vortexed and placed in a thermocycler for RT, using the following program: 52°C for 15 min, 80°C for 10 min, stored at 10°C.

#### Pre-amplification

The samples were cooled on ice and supplied with 15 μL of amplification master mix per sample. The amplification master mix contained 12.5 μL of KAPA HiFi Hot start ReadyMix (Roche, Basel, Switzerland, cat # KK2601) and 2.5 μL of H_2_O.

The mixture was then pulse-vortexed and placed into a thermocycler for pre-amplification, using the following program: 98°C for 3 min; 23 cycles of 98°C for 20 s, 67°C for 15 s, and 72°C for 6 min; 72°C for 5 min; stored at 10°C.

#### cDNA purification I

The samples were filled up with H_2_O to a final volume of 50 μL, then each sample was supplied with a 0.6× sample volume of solid-phase reversible immobilization (SPRI) beads (Beckman Coulter, Brea, USA). The samples were mixed by pulse-vortexing and were let to incubate for 15 min at room temperature.

Following the incubation, the samples were placed on a magnet, and after bead sedimentation the supernatant was carefully removed. The samples were immediately eluted by adding 13 μL of fresh H_2_O to the beads, pulse vortexing them, and incubating for 3 min at room temperature.

Following incubation, the samples were placed back on a magnet, and 12 μL of the supernatant was carefully transferred into new plates.

#### Quality control I

The eluted samples were analyzed using the Agilent Fragment Analyzer Systems in combination with the HS NGS Fragment kit (Agilent, Santa Clara, USA, cat #DNF-474) according to the manufacturer’s recommendations. Samples were selected for further processing based on their cDNA content and fragment analysis profile between the 200 and 1,500 bp range.

#### Primer annealing

Lyophilized Tn5ME-A, Tn5ME-B, and Tn5RMErev primers (for sequences see Table S7) were re-suspended in annealing buffer (50 mM NaCl, 40 mM Tris-HCl pH 8.0) to a concentration of 100 μM.

Tn5ME-A or Tn5ME-B was mixed with Tn5Merev in a 1:1 ratio in a 50 μM final concentration and annealed in a thermocycler, using the following program: 95°C for 5 min, cool down to 65°C (ramp-down rate: 0.1°C/s), keep at 65°C for 5 min, and finally slowly cool down to 4°C (ramp-down rate: 0.1°C/s).

The annealed oligonucleotides were diluted to 35 μM with H_2_O and store at −20°C until use.

#### Transposase loading

The Tn5 enzyme (0.9 mg/mL, Tn5_(R27S),E54K,L372P_, EMBL-Protein Expression and Purification Core Facility, Heidelberg, Germany) was diluted in H_2_O in a 1:1 ratio, in 10 μL final volume, then mixed with 0.7 μL annealed Tn5ME-A/Tn5Merev and Tn5ME-B/Tn5Merev oligoes, pulse-vortexed, and placed in a shaker at 23°C, 350 rpm for 45 min. Following pre-loading, the Tn5 mix was diluted in 1:450 ratio in H_2_O and was kept on ice until use.

As cDNA concentration after pre-amplification was generally low (median 0.4 ng/μL), 4 μL of each sample was transferred into a new 96-well plate and was concentrated to ∼1–1.5 μL using a SpeedVac (Eppendorf, Hamburg, Germany) vacuum concentrator.

Following concentration, each sample was supplied with 1.25 μL Tn5 mix (1:450 dilution), 1.25 μL 4× tagmentation mix (40 mM Tris-HCl pH 7.5, 40 mM MgCl_2_), and 1.25 μL 100% DMF (Sigma-Aldrich, St. Louis, USA), mixed well by pipetting, and incubated on 55°C for 3 min in a thermocycler.

Following incubation, the samples were cooled on ice for a short time, then they were supplied with 1.25 μL of 0.2% SDS, mixed well, and incubated on room temperature for 5 min to stop the tagmentation. Following incubation, the samples were stored on ice until further use.

#### PCR amplification

Samples were supplied with 6.75 μL Kappa HiFi Hot start ReadyMix (cat # KK2601), 0.75 μL 100% DMSO (Sigma-Aldrich, St. Louis, USA), and 2.5 μL i5/i7adapter index primer mix (Table S4).

The samples were mixed and amplified in a thermocycler using the following program: 72°C for 3 min; 18 cycles of 95°C for 30 s, 98°C for 20 s, 58°C for 15 s, and 72°C for 30 s; 72°C for 3 min, stored at 10°C.

#### cDNA purification II

Following final PCR amplification, the samples were filled up with H_2_O to a final volume of 50 μL, then each sample was supplied with a 0.7× sample volume of SPRI beads.

The samples were mixed by pulse vortexing and were let to incubate for 10 min on room temperature, then the samples were placed on a magnet, and after bead sedimentation the supernatant was carefully removed. The samples were washed 2× with 200 μL 80% EtOH (30 s each) and were dried for 2 min at room temperature.

After drying, the samples were eluted by adding 14 μL of fresh H_2_O to the beads, pulse-vortexing them, and incubating for 3 min at room temperature. Following incubation, the samples were placed back on a magnet, and 13 μL of the supernatant was carefully transferred into new plates.

#### Quality control II and concentration adjustment

The transferred samples were analyzed using the Agilent Fragment Analyzer Systems in combination with the HS NGS Fragment kit (Agilent, Santa Clara, USA, cat #DNF-474) according to the manufacturer’s recommendations.

The molarity of each sample was calculated based on their fragment analysis profile in the 200–1,500 bp range, using the ProSize data analysis software (Agilent, Santa Clara, USA, version 4.0.2.7). The resulting .csv files were further processed in R v.4.2.3, where a median molar concentration of 6.95 nM was calculated (see the [CTC_scRNA-seq_fragment_cleanup_and_analysis.R] analysis script on GitHub [https://github.com/UBE2C/psma-epcam-scrna-flow]).

Samples were divided into above median and below median groups based on the calculated median molar concentration. Samples above median molar concentration were diluted to the median concentration in a 5 μL final volume in a new 96-well plate. Following dilution, the samples were pooled by transferring approximately 6.95 fmol from each sample into a 1.5 mL DNA LoBind Tube (Eppendorf, Hamburg, Germany). Samples below 1 nM were discarded.

The resulting sample pool was concentrated using SPRI beads as described at the end of sample tagmentation, except that the bead volume was 1× of the sample volume, and the final elution volume was 25 μL.

#### Sample multiplexing and final quality control

Below median and above median sample pools were mixed in an equimolar concentration and was assessed using a Bioanalyzer 2100 (Agilent, Santa Clara, USA). The measurement showed a relatively high number of fragments between the lengths of 150 and 300 bp. The final sample pool was once again cleaned by SPRI beads, as described at the end of sample tagmentation, except that the bead volume was 0.65× of the sample volume. Following cleanup, the final sample multiplex was assessed one last time by a Bioanalyzer 2100 and was used for sequencing.

### RNA sequencing

The prepared dual barcoded libraries were sequenced on an Illumina NextSeq2000 sequencer using the P3 flow cell in a single-end 50 bp configuration.

### Flow cytometry data analysis

Index-sort FCS files (C4-2 control, patient) were loaded into R v.4.2.3 with flowCore v.2.16.

#### Data loading and quality control

The obtained .fcs files were loaded into two separate R lists (C4-2 control list and sample list) using the flowCore::read.FCS function (default arguments). The median of the MFI) values of each recorded channel were calculated for the C4-2 control samples. As part of the quality control (QC) process, any duplicated entries were removed.

To ensure the recorded control samples are fit to be used for normalization, the control samples were evaluated by calculating and plotting the grand median, median absolute deviation (MAD), and median absolute deviation of the medians (MADM) for each control sample. Comparison between the grand median values was done using a Mann-Whitney U test (Figure S4B).

Next, any sample name errors for sorted CTCs were fixed, and any sort control samples were filtered out to match the sorted samples. The cleaned and fixed patient sample sort data were merged into a single data frame for MFI normalization.

#### Data normalization, MFI artifact correction, and filtering

MFI values of all fluorescent channels were normalized, to account for the sort-to-sort variation and to facilitate a more precise sample comparison. MFI values of each sorted patient sample were divided by the session-matched control median MFI. Samples from patients with unknown treatment outcomes were excluded, and negative EpCAM and PSMA MFI values—artefacts of baseline subtraction—were imputed. To ensure that imputed values remained close to the detection baseline while avoiding value duplication, a channel-specific drawing set was constructed from the 10 lowest positive MFI values. Two values were randomly drawn from this set, and their mean was used as the imputed value.

Following imputation, the data were filtered on both EpCAM and PSMA channels to remove outliers with exceptionally high and low MFI values (Figures S4C and S4D).

#### Marker dynamics, population distribution, and statistical analysis

Following data preparation, parameters such as marker expression, marker distribution, and marker dynamics over treatment cycles were analyzed using the base stats R package. Statistics involved median, MAD, Mann-Whitney U test, Shapiro-Wilk test of normality, and χ^2^ goodness-of-fit test, respectively.

The obtained data were visualized using ggplot2 v.3.5.1, ggsci v.3.2.0, and ggpubr v.0.6.0 R packages and Affinity Designer 2. For the detailed analysis, see the [flow Cytometry_analysis_script_V1.0.0.R] analysis script on GitHub [https://github.com/UBE2C/psma-epcam-scrna-flow].

### scRNA-seq: Read alignment and filtering

Following sequencing, raw reads were demultiplexed, adapter- and quality-trimmed, and aligned to the GRCh38 reference with STAR v.2.7.10b (--quantMode). The resulting ReadsPerGene.out.tab files were parsed in R v.4.3.1–4.4.1 to extract ENSEMBL ID-based gene identifiers and unstranded read counts, which were aggregated into a single gene-by-cell matrix and combined with sample metadata into a Seurat v.4.3.0.1 object for downstream analysis.

QC was performed to remove potential duplets and low-quality cells from the dataset. Cells with <4,000 total counts, <1,000 or >7,800 detected genes, or >75% mitochondrial reads were removed (Figures S5B–S5E). A lenient mitochondrial cutoff was chosen, given our dual live-dead/apoptotic sorting, potential radiation-induced mitochondrial upregulation, and the higher MT read fraction typical of Smart-Seq2 (for a detailed explanation see supplemental methods).

### scRNA-seq: Data normalization, clustering, and composition analysis

Gene counts were normalized and variance-stabilized with Seurat’s SCTransform (glmGamPoi method, glmGamPoi package v.1.14.0) before PCA on the top 3,000 highly variable genes. The first 20 PCs—determined by an elbow plot—were used to compute a UMAP embedding (dims 1:20) for visualization and downstream clustering. Cell neighbors were identified with FindNeighbors (dims = 1:20), and clusters were defined via FindClusters (resolution = 0.6). Cluster composition was then compared across treatment variables (detailed/overall response status, treatment type, cycle, cell-cycle phase, and surface marker status) by χ^2^ tests of independence (α = 0.05).

### scRNA-seq: Differential gene expression and gene set enrichment analysis

Differential expression analysis between clusters was performed with Seurat v.5.0.2’s FindMarkers (default Wilcoxon test). Resulting genes were annotated with gene symbols and Entrez IDs, while entries lacking symbols were removed. Genes were ranked by log_2_ FC × (–log_10_ adjusted *p* value) and plotted as a volcano plot (ggplot2; see Figure 5A).

GSEA was conducted with irGSEA v3.3.2 using standard settings and the following msigbdr v.7.5.1 collections: C2 (BIOCARTA, KEGG, REACTOME, WIKIPATHWAYS), C3 (TFT:GTRD), C4 (CGN, CM), C5 (GO:BP, GO:MF, GO:CC), C6, and H. Enrichment results were integrated and visualized via irGSEA.integrate and irGSEA.heatmap. Hub genes within significant pathways were identified with irGSEA.hub and filtered for entries with a rank-correlation score of at least 0.2, a Spearman’s *p* value of <0.05 in at least two methods, and with a significant DEG status. Data visualization was done using ggplot2 v.3.5.1, ggsci v.3.2.0, and ggpubr v.0.6.0 R packages and Affinity Designer 2. For the detailed analysis, see the [CTC_scRNA-seq_data_analysis_script_V1.0.0.R] analysis script on GitHub [https://github.com/UBE2C/psma-epcam-scrna-flow].

## Data and code availability

All data supporting the findings of this study are available from the corresponding author on reasonable request.

## Acknowledgments

Co-funding of the research group Molecular Biology of Systemic Radiotherapy was made available by industrial collaboration with Bayer AG, Berlin, Germany. We would like to thank the EMBL Flow Cytometry Core facility and the EBML Gene Core for all the help and support they provided for the project. This research received no external funding.

## Author contributions

Conceptualization, G.B., M.R., C.K., and M.B.-S.; patient recruitment and blood collection, C.K.; methodology, G.B., U.B.-W., B.R., and M.B.-S.; investigation, G.B., U.B.-W., B.R., and M.B.-S.; data analysis and interpretation, G.B., J.L., B.R., and M.B.-S.; visualization, G.B. and B.R.; writing—original draft, G.B. and M.B.-S.; writing—review & editing, G.B., B.R., J.L., M.R. C.K., F.B., A.M., V.B., and M.B.-S.; funding acquisition, M.B.-S.; supervision, G.B. and M.B.-S.

## Declaration of interests

Patent application on PSMA-617 has been filed by German Cancer Research Center (DKFZ) and University Clinic (UKHD) in Heidelberg, Germany. U.B.W., C.K., and M.B.S. are co-inventors of this patent. No other potential conflicts of interest relevant to this article were reported.

## Supplemental information

Document S1. Figures S1–S9, Tables S1, S3, and S5–S7, and supplemental methods

Table S2. Differentially expressed genes (DEG) table A table showing the significant (p_adjusted_value <0,05; Bonferroni-correction) DEGs between cluster 0 and cluster 1 based on the Wilcoxon rank-sum test.

Table S4. Index primers A table showing the i5–i7 index primer pairs and their sequences used during library preparation.

Document S2. Article plus supplemental information
