# Supplementary material for: EpCAM–PSMA: Potential predictors of treatment outcomes for PSMA-targeted alpha therapies in metastatic castration-resistant prostate cancer
Source: Mol Ther Oncol. 2026 Feb 2;34(1):201143. doi: 10.1016/j.omton.2026.201143 (PMC12925578; doi:10.1016/j.omton.2026.201143)
Supplement: Document S1. Figures S1–S9, Tables S1, S3, and S5–S7, and supplemental methods [file mmc1.pdf]

OMTON, Volume *34*

Supplemental information

EpCAM–PSMA: Potential predictors of treatment outcomes for PSMA-targeted alpha therapies in metastatic castration-resistant prostate cancer

Gábor Bakos, Ulrike Bauder-Wüst, Jonathan Landry, Mareike Roscher, Beáta Ramasz, Frank Bruchertseifer, Alfred Morgenstern, Clemens Kratochwil, Vladimír Beneš, and Martina Benešová-Schäfer

Supplemental Figures

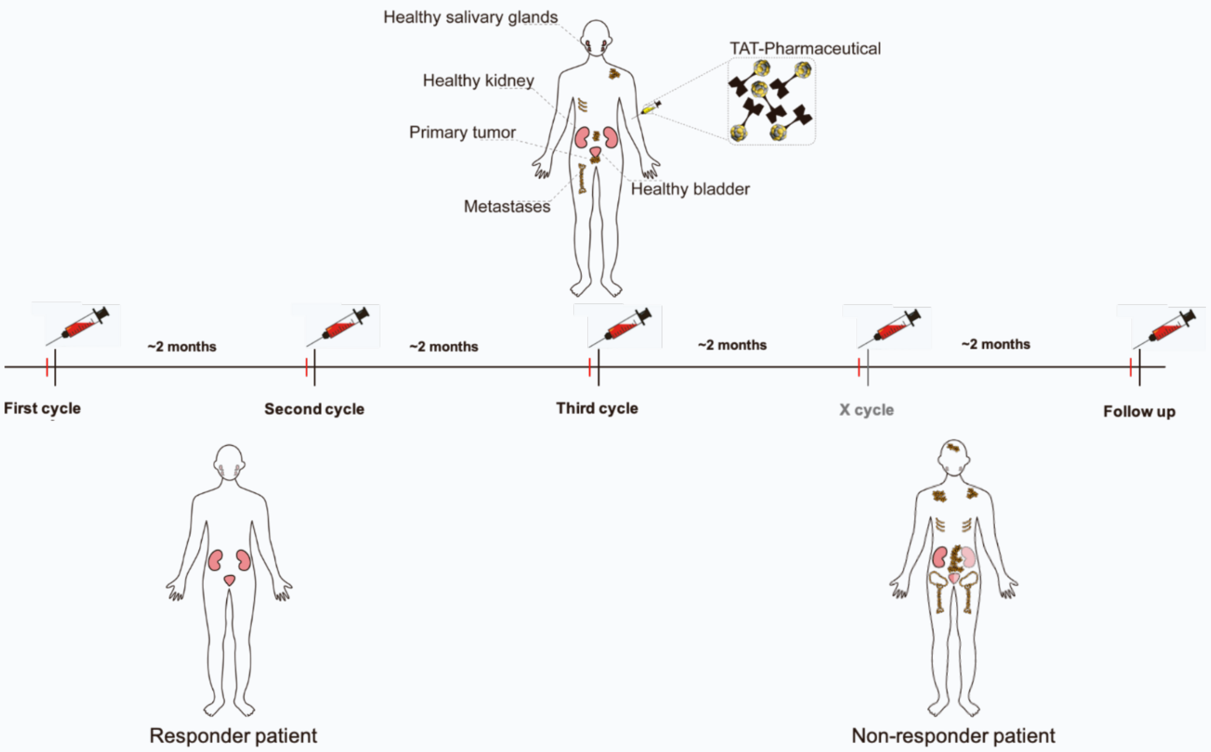

Figure S1. Blood collection layout. Patients with mCRPC who are receiving [^{177}Lu]Lu-PSMA-617 and [^{225}Ac]Ac-PSMA-617 treatment were selected by physicians for blood collection. Blood samples were collected before drug administration for each treatment cycle. The blood samples were prepared for storage the same day as the collection. Later, the patients were classified by trained physicians to three groups: Responder group, Nonresponder group and Unclassified patients. Only the Responder and Nonresponder groups were selected for further analysis.

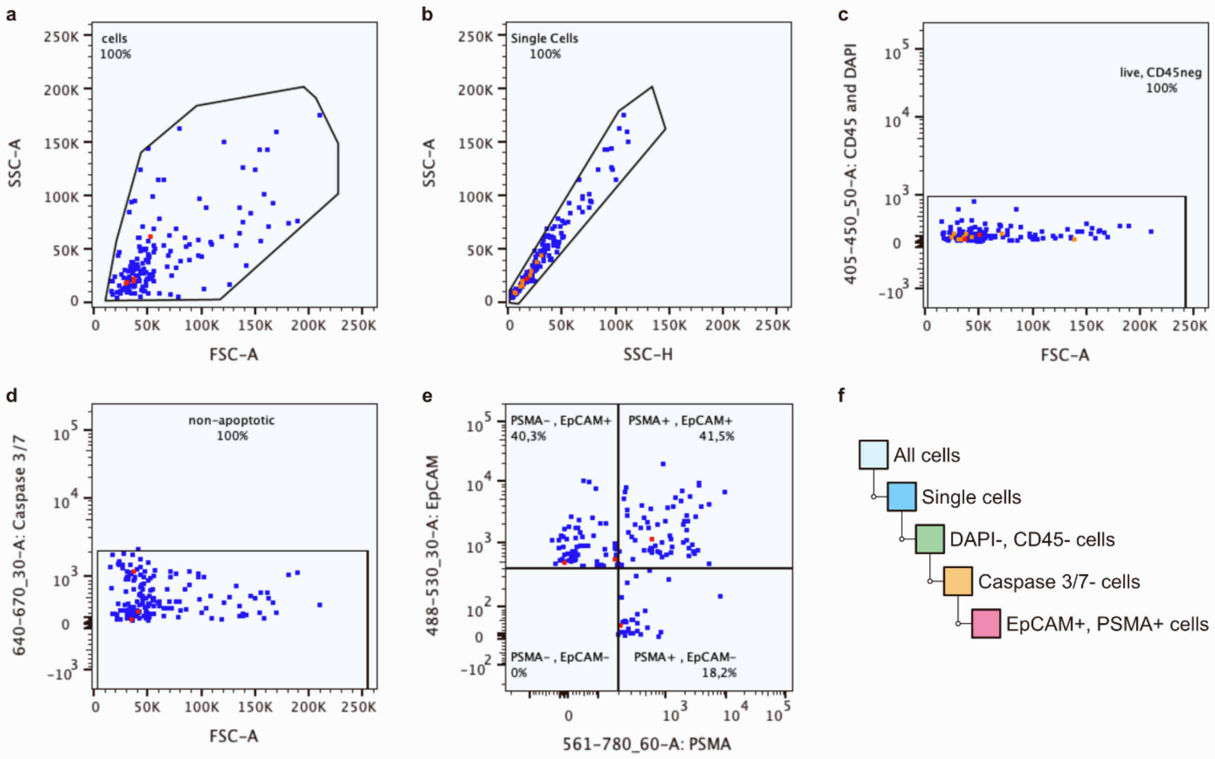

Figure S2. Gating strategy for CTC sorting. The data shown are concatenated from multiple indexed single-cell sorts and include only the cells that were successfully sorted. **(a)** The first step where, cells were identified while debris and aggregates were excluded. **(b)** Next, doublets were removed based on side scatter area (SSC-A) versus side scatter height (SSC-H) parameters. **(c)** Dead cells and $CD45^{+}$ leukocytes were simultaneously excluded using a combined DAPI and CD45 staining gate. **(d)** To avoid sorting apoptotic cells, caspase-positive cells were excluded. **(e)** In the final step, three distinct populations of interest were identified based on PSMA and EpCAM expression. Only cells positive for at least one of these markers were sorted, while cells negative for both were excluded. **(f)** Gating hierarchy, with each population corresponding to the plots shown above.

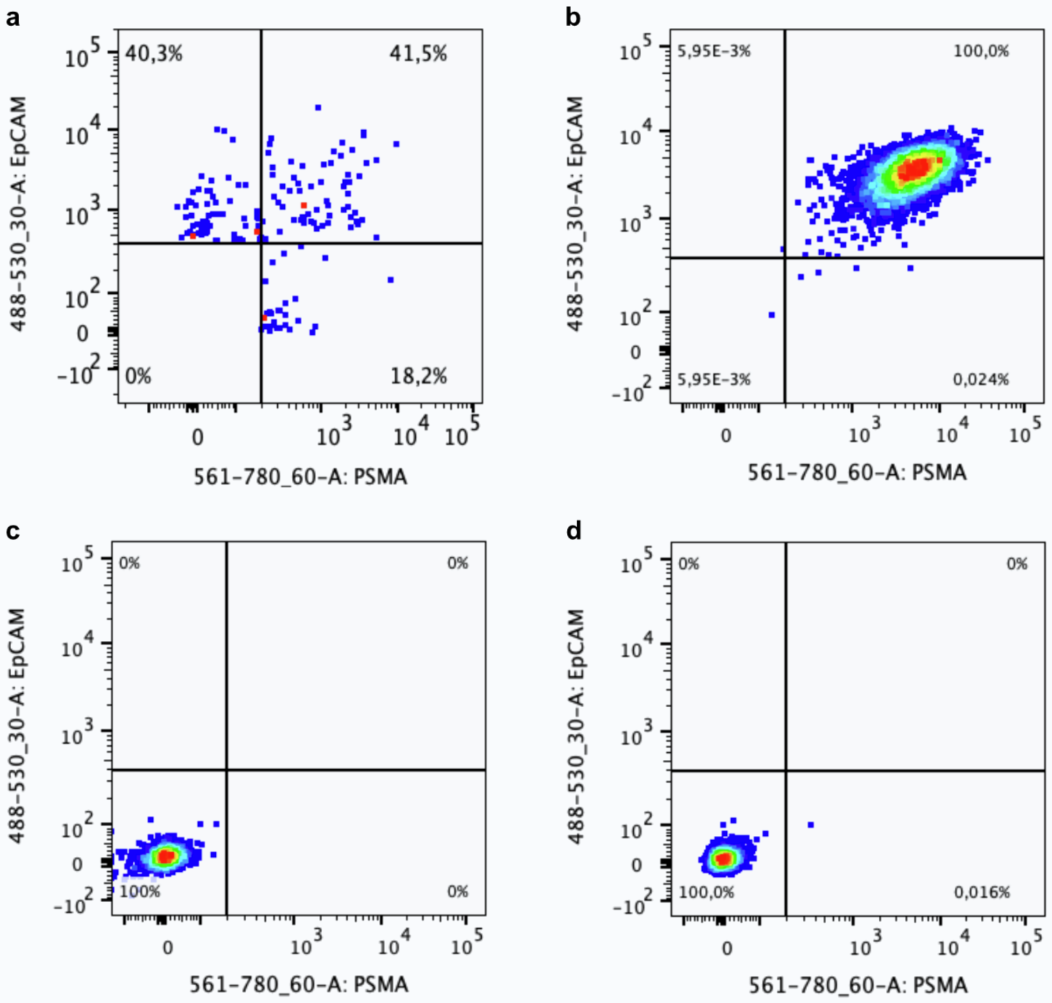

Figure S3. Controls used to establish accurate gating for CTC identification. (a) A panel showing concatenated data from multiple indexed single-cell sorts. All cells depicted were classified as circulating tumor cells (CTCs), as they expressed at least one of the markers PSMA or EpCAM. **(b)** The positive control, consisting of C4-2 prostate cancer cells, which are known to express both PSMA and EpCAM. **(c)** $CD45^+$ leukocytes that underwent the same staining protocol as the pre-enriched $CD45^-$ cell fraction; these cells were stained with the full antibody panel, including PSMA and EpCAM. **(d)** The negative control, which was not stained with any antibodies and served to assess background fluorescence.

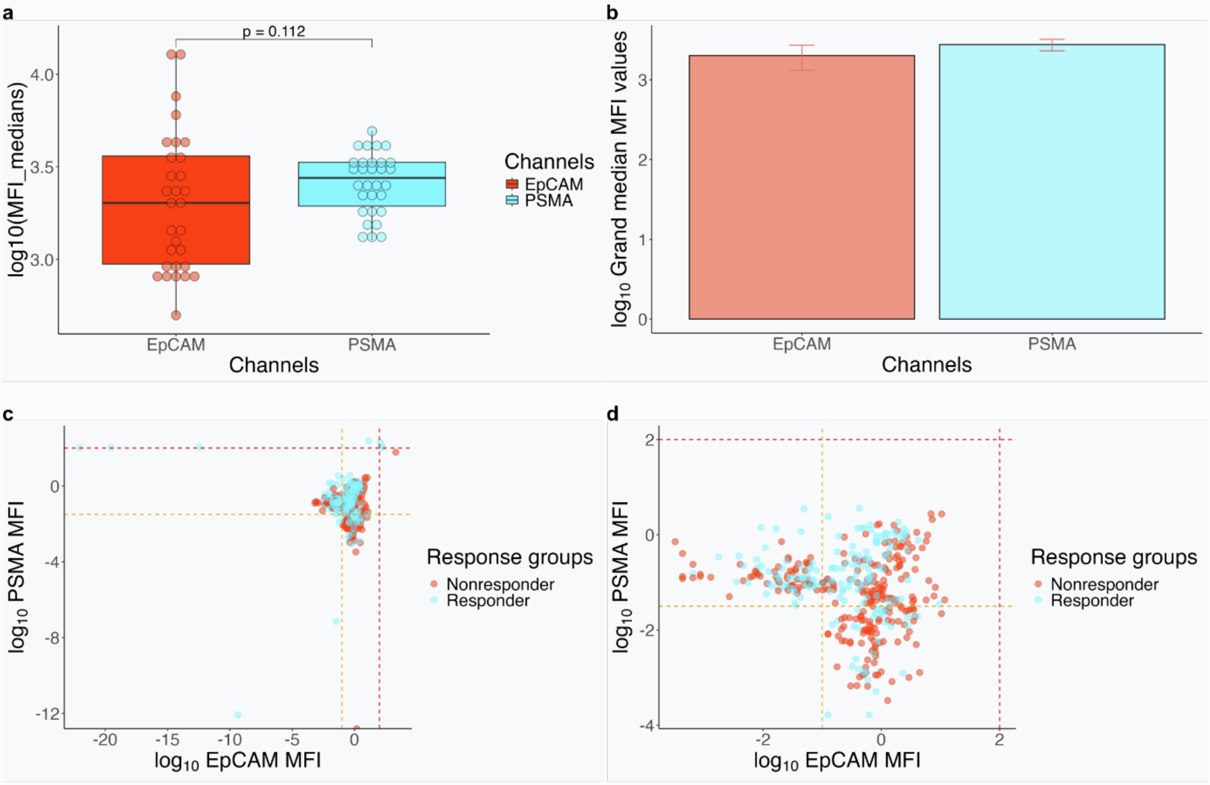

Figure S4. Control C4-2 surface marker levels and CTC filtering after FACS sorting. (a) A box plot showing the median EpCAM and PSMA MFI values on the sorted C4-2 control cells from multiple FACS sorts. The p-value (0.112) was calculated using the Mann-Whitney-U-test. **(b)** A box plot showing the grand median MFI values for EpCAM and PSMA on the control C4-2 cells, and the median absolute deviation of the medians (MADM). The grand median was calculated from multiple FACS experiments. **(c)** A scatter plot showing the CTC filtering strategy where cells with outlying EpCAM or PSMA values were excluded from the downstream analysis. **(d)** A scatter plot showing the distribution of sorted CTCs, which passed the filtering step.

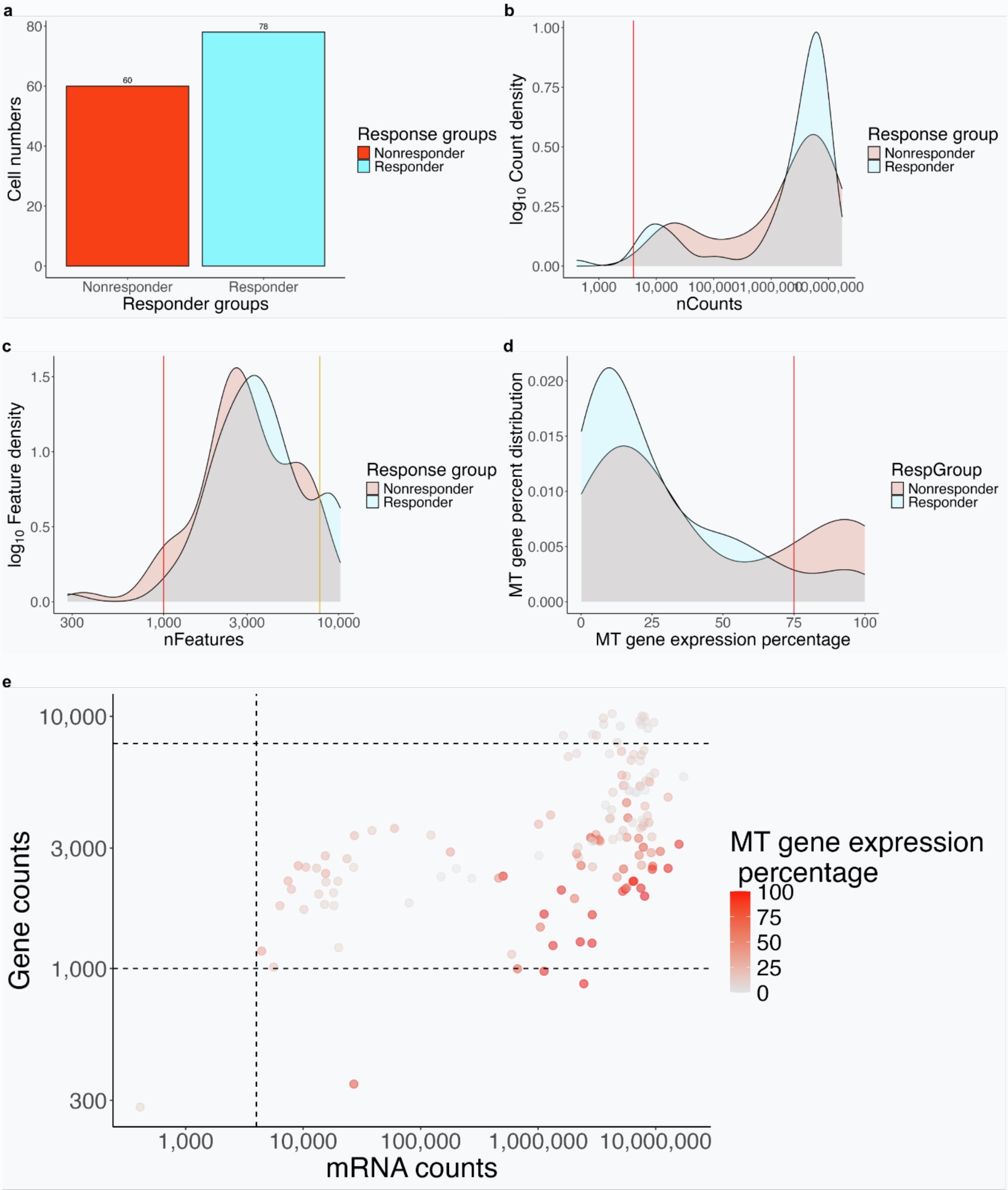

Figure S5. CTC filtering based on various metrics. (a) A bar chart showing the total number of sequenced CTCs from both Responder and Nonresponder samples before filtering. **(b)** A density plot showing the distribution of RNA counts obtained from the sequenced CTCs before filtering. The red vertical bar represents the minimum number of accepted counts for filtering. **(c)** A density plot showing the distribution of detected genes (Features) in the sequenced CTCs before filtering. The red vertical bar represents the minimum number of accepted genes, while the orange bar represents the maximum number of accepted genes for filtering. **(d)** A density plot showing the distribution of mitochondrial (MT) gene percentage obtained from the sequenced CTCs before filtering. The red vertical bar represents the maximum accepted MT gene percentage for filtering. **(e)** A scatter plot showing the distribution of the sequenced CTCs based on RNA and gene counts, and colored by their MT gene expression percentage before filtering. The dotted lines represent the various RNA and gene count thresholds used for filtering.

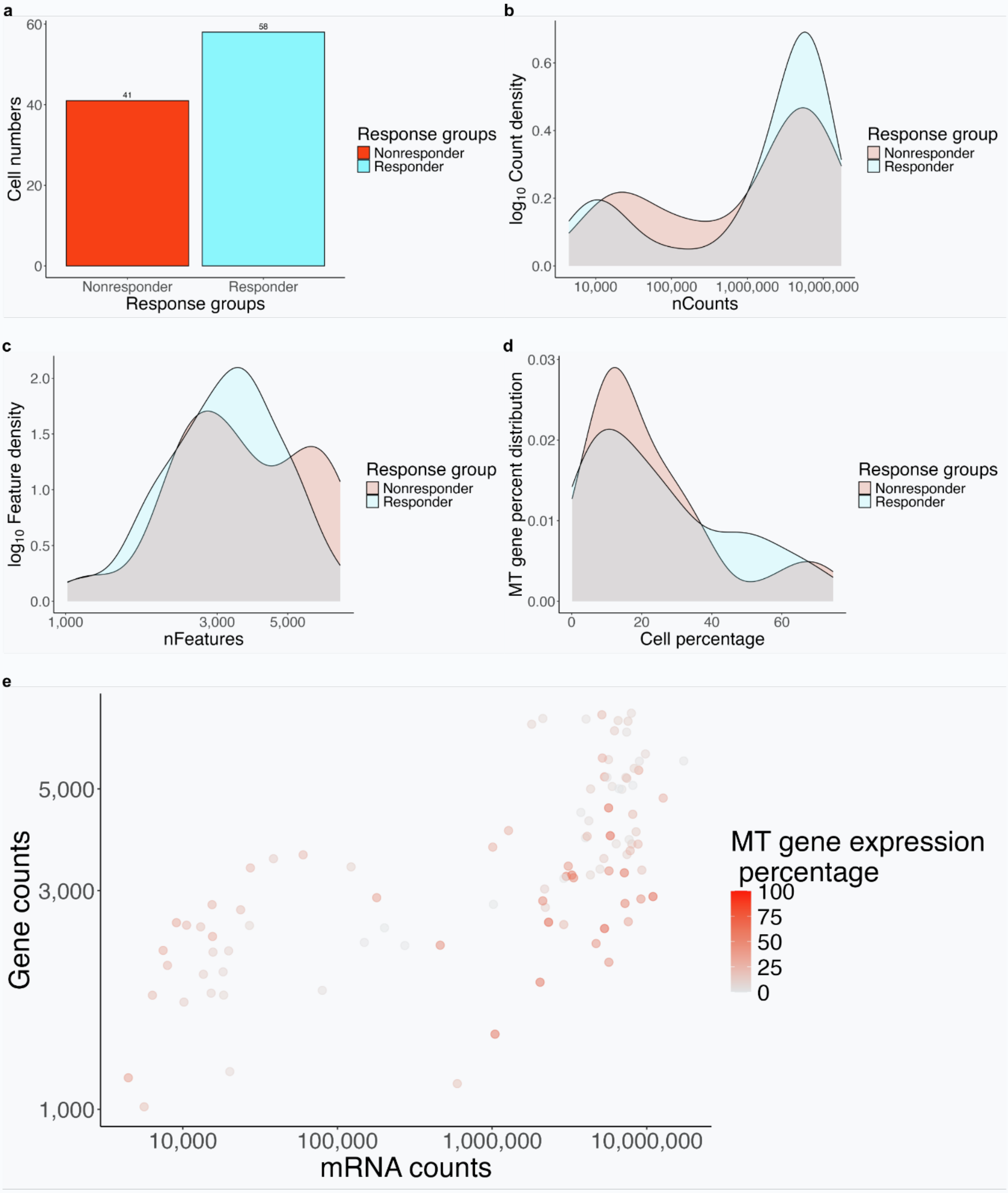

Figure S6. Various CTC metrics after filtering. (a) A bar chart showing the total number of sequenced CTCs from both Responder and Nonresponder samples after filtering. **(b)** A density plot showing the distribution of RNA counts in the filtered CTCs. **(c)** A density plot showing the distribution of detected genes (Features) in the filtered CTCs. **(d)** A density plot showing the distribution of mitochondrial (MT) gene percentages in the filtered CTCs. **(e)** A scatter plot showing the distribution of the filtered CTCs based on RNA and gene counts, and colored by their MT gene expression percentage.

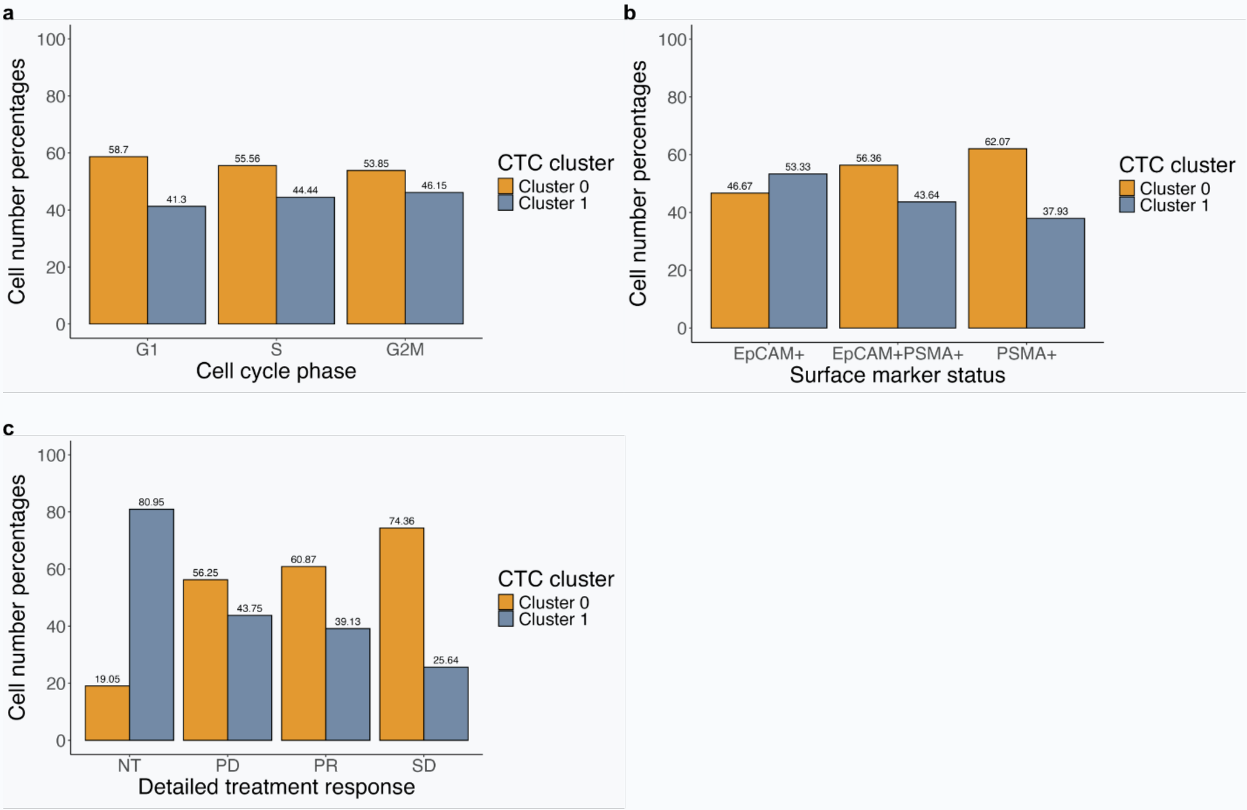

Figure S7. CTC clusters and their correlation to various treatment variables. (a) A bar chart showing the distribution of CTCs between clusters 0 and 1, based on their cell cycle phase. **(b)** A bar chart showing the distribution of CTCs between clusters 0 and 1, based on their surface marker status. **(c)** A bar chart showing the distribution of CTCs between clusters 0 and 1, based on detailed patient response status. NT = not treated, PD = progressive disease, PR = partial response, SD = stable disease.

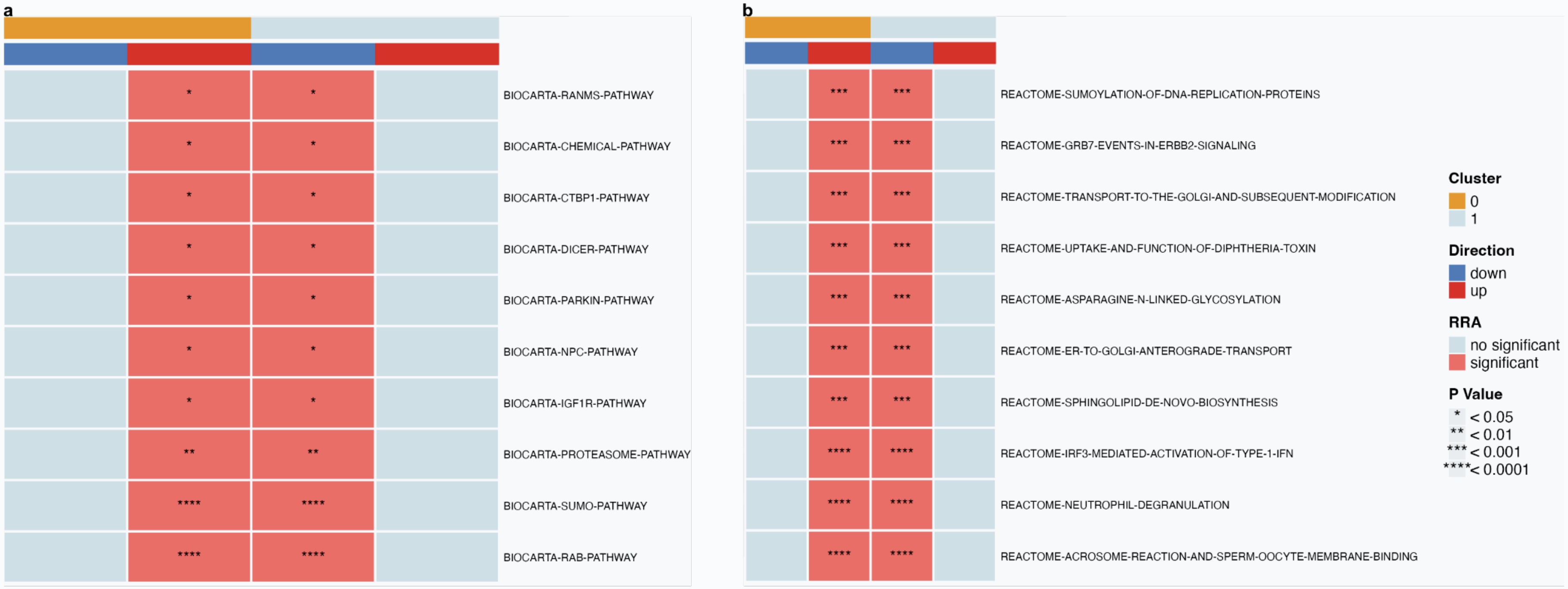

Figure S8. Differentially regulated pathways among the CTC clusters found by irGSEA. **(a)** A heat map showing the 10 most significantly impacted pathways based on the BIOCARTA gene sets. **(b)** A heat map showing the 10 most significantly impacted pathways based on the REACTOME gene sets.

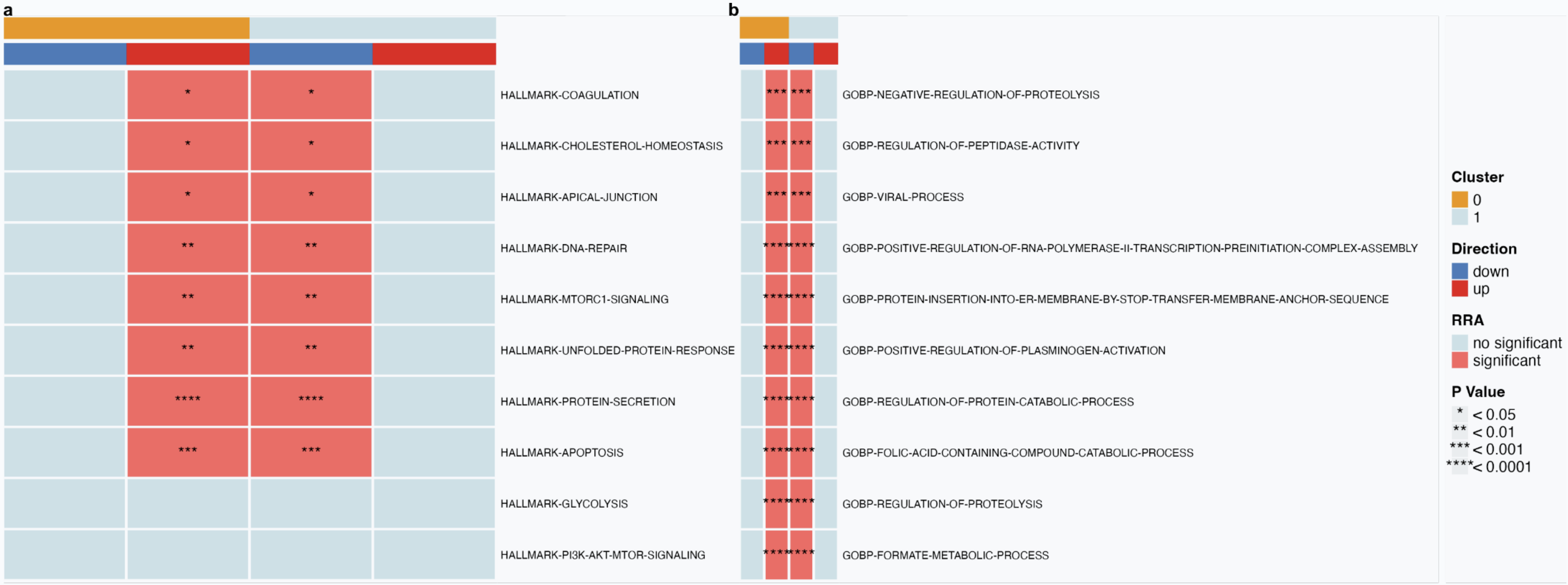

Figure S9. Differentially regulated pathways among the CTC clusters found by irGSEA. (a) A heat map showing the 10 most significantly impacted pathways based on the HALLMARK gene sets. **(b)** A heat map showing the 10 most significantly impacted pathways based on the GENE ONTOLOGY: BIOLOGICAL PROCESSES (GO:BP) gene sets.

Supplemental Tables

Table S1. PSA values over the treatment cycles. A table showing changes in PSA values for patients whose CTC samples were successfully sequenced and included in the final scRNA-seq analysis during a combined [225Ac]Ac-PSMA-617 - [177Lu]Lu-PSMA-617 treatment.

Patient code	Baseline PSA (ng/ml)	Follow up 1 (FU1) (ng/ml)	FU2 (ng/ml)	FU3 (ng/ml)	FU4 (ng/ml)	FU5 (ng/ml)	Status at each cycle	Final Response status
ALM60C8002	190	174	80,32	47,39	20,82	15,39	1.-5. PR	Responder
ALM63C9005	328,2	210,9					1. SD	Nonresponder
ALM69C5003	56,17	84,42	105,75				1. SD; 2 PD	Nonresponder
ALM70C8006	688,65	443,74					1. SD; 2 PD	Nonresponder
ALM74C8008	4,7	3,13	1,01				1-2 PR	Responder
ALM76C6003	111,27	373,09					1. PD	Nonresponder
ALM77C5004	68,9	13,35	8,19				1. SD; 2. PR	Responder
ALM77C5005	38,98	17,88	5,36				1. SD; 2. PR	Responder
ALM78C7005	332,8	248,7	83,8	26,2	8,9		1-4 PR	Responder
ALM79C9003	42,7	111,29					1. PD	Nonresponder
ALM80C6007	823,69	633,97	251	480,47			1. SD; 2. SD; 3. SD	Nonresponder
ALM80C7001	119,56	4,34	0,17				1.-2. PR	Responder
ALM80C8004	137,69	66,96	30,5	15,34			1. PR; 2. PR; 3. PR	Responder
ALM81C2001	308,6	48,84	16,39	5,11			1. PR; 2. PR; 3. PR	Responder
ALM82C6002	88,21	26.95					1. PR; 2. PR	Responder
ALM82C7004	12,77	19,5	47,8				1. SD; 2. PD	Nonresponder
ALM83C8001	0,5	0,33	<0,1				1.-2. PR	Responder
ALM88C3002	846	464,25	1684,57				1. SD; 2. PD	Nonresponder
LM66C3003	6,4	4,21	5,58	12,32	13,3	23,8	1-5. SD	Nonresponder
LM67C6011	584	99,4	28,2	4,6			1-3. PR	Responder
LM68C6005	715	578,82	537,08	358,53			1. PR; 2. SD; 3. PR	Responder
LM75C7006	153,2	80,5	41,7	22,5			1-3. PR	Responder
LM81C6017	82,9	15,5	186,9	378			1. PR ; 2. PD; 3. PD	Nonresponder

Table S2. Differentially Expressed Genes (DEG) table. A table showing the significant (p_adjusted_value < 0,05; Bonferroni-correction) DEGs between Cluster 0 and Cluster 1 based on the Wilcoxon Rank Sum test.

Table S3. irGSEA hub gene analysis table. This table presents the hub genes whose expression significantly differs between Cluster 0 and Cluster 1. It includes the genes, their associated MSigDB database entries, the pathways they impact, and relevant references provided in the Supplemental Materials.

ENSEMBL_ID	Gene_symbol	General_expression_in_PCa	DEG_staus-Cluster0_vs_1	References	MSigDB	Pathway
ENSG00000015475	BID	Downregulated	Upregulated	[4, 5]	BIOCARTA	Chemical_pathway
ENSG00000100401	RANGAP1	-	Upregulated	[6]	BIOCARTA	Nuclear_Pore_Complex_pathway
ENSG00000103769	RAB11A	Upregulated	Upregulated	[7, 8]	BIOCARTA	RAB_pathway
ENSG00000116030	SUMO1	Upregulated	Upregulated	[9, 10, 11, 12, 13, 14]	BIOCARTA	SUMO_pathway and CTBP1_pathway
ENSG00000121879	PIK3CA	Upregulated	Downregulated	[15, 16, 17]	BIOCARTA	IGFR1R_signaling
ENSG00000123595	RAB9A	-	Upregulated	[7]	BIOCARTA	RAB_pathway
ENSG00000126261	UBA2	-	Upregulated	[9, 11]	BIOCARTA	SUMO_pathway
ENSG00000140443	IGF1R	Upregulated	Upregulated	[18, 19]	BIOCARTA	IGFR1R_signaling
ENSG00000144566	RAB5A	Downregulated	Downregulated	[7, 20]	BIOCARTA	RAB_pathway
ENSG00000165806	CASP7	Downregulated	Upregulated	[21, 22]	BIOCARTA	Chemical_pathway
ENSG00000168118	RAB4A	Downregulated	Upregulated	[7, 20]	BIOCARTA	RAB_pathway
ENSG00000188612	SUMO2	-	Upregulated	[9, 23, 11]	BIOCARTA	SUMO_pathway
ENSG00000074800	ENO1	Upregulated	Upregulated	[24]	GOBP	Plasminogen_activation
ENSG00000105707	HPN	Upregulated	Downregulated	[25, 26]	GOBP	Plasminogen_activation
ENSG00000110330	BIRC2	-	Upregulated	[27, 28, 29]	GOBP	K63-linked_ubiquitination
ENSG00000111530	CAND1	Upregulated	Upregulated	[30]	GOBP	RNA-POL-II_preinit._complex_assembly
ENSG00000141510	TP53	Upregulated	Upregulated	[31, 32, 33]	GOBP	RNA-POL-II_preinit._complex_assembly
ENSG00000169139	UBE2V2	Upregulated	Upregulated	[34, 35, 36, 37, 38]	GOBP	K63-linked_ubiquitination
ENSG00000177889	UBE2N	Upregulated	Upregulated	[34, 35, 36]	GOBP	K63-linked_ubiquitination
ENSG00000244687	UBE2V1	-	Upregulated	[34, 35, 36]	GOBP	K63-linked_ubiquitination
ENSG00000152669	CCNO	-	Upregulated	[39, 40, 41, 42]	HALLMARK	DNA_repair
ENSG00000184432	COPB2	Upregulated	Upregulated	[43, 44, 45]	HALLMARK	protein_secretion
ENSG00000186141	POLR3C	Upregulated	Upregulated	[46, 47]	HALLMARK	DNA_repair
ENSG00000205302	SNX2	Upregulated	Upregulated	[48, 49, 50]	HALLMARK	protein_secretion
ENSG00000087586	AURKA	Upregulated	Upregulated	[51, 52, 53, 54, 55]	HALLMARK and REACTOME	mTROC1_signaling and Sumoylation_of_DNA-replication_proteins
ENSG00000065361	ERBB3	Upregulated	Downregulated	[56, 57, 58, 59]	REACTOME	GRB7_events_in_ERBB2_signaling
ENSG00000134690	CDCA8	Upregulated	Upregulated	[60, 61, 62, 55, 63]	REACTOME	Sumoylation_of_DNA-replication_proteins
ENSG00000141736	ERBB2	Upregulated	Upregulated	[64, 65, 66]	REACTOME	GRB7_events_in_ERBB2_signaling
ENSG00000196655	TRAPPC4	-	Upregulated	[67, 68]	REACTOME	ER_to_golgi_transsport
ENSG00000198431	TXNRD1	Upregulated	Upregulated	[69, 70]	REACTOME	Diphtheria_toxin_signaling

Table S4. Index primers. A table showing the i5 - i7 index primer pairs and their sequences used during library preparation.

Table S5. Optical configuration table. A table showing the optical configuration of the BD FACSAria Fusion Flow Cytometer used for single-cell sorting.

Laser (nM)	Channel Name-Collection Filter	LP	Fluorochromes (Some)
**355 UV**	450/50	-	DAPI, DyLight 350
	525/50	505	BUV496
405 **Violet**	405-450/50	-	DAPI, Pacific Blue, AF 405, BV421, v450, BFP, CFP, CellTrace Violet
	405-525/50	505	Sytox Blue, L/D Fix Aqua, AmCyan, v500, AF 430, Pacific Orange, Qdot 525
	405-610/20	595	BV605, Qdot 605, eFluor 605NC, L/D Fix Yellow, Pacific Orange
	405-660/20	630	BV650, Qdot 655
	405-710/50	690	BV711
	405-780/60	750	BV786
488 **Blue**	488-530/30	502	FITC, GFP, YFP, Alexa 488, mCitrine, Syto9, Sytox Green, Calcein
	488-710/50	690	PerCP-Cy5.5, DRAQ5, PerCP, efluor710
561 **Yellow-Green**	561-586/15	-	PE, DsRed, RFP, tdTomato, Sytox Orange
	561-610/20	600	PI, mCherry, Texas Red, PE-CF594
	561-670/14	630	7-AAD, PE-Cy5
	561-710/50	685	PE-Cy5.5, PE-Cy5, 7-AAD
	561-780/60	735	PE-Cy7, APC-Vio770, PE-Vio 770
633 **Red**	640-670/30	-	APC, AF 647, DRAQ5, DRAQ7, L/D Fix Far Red, TO-Pro3
	640-730/45	690	AF 700, APC-CY5.5, DRAQ7, Dye Cycle Ruby
	640-780/60	755	APC-Cy7, AF 750, APC-eFluor 780, APC-H7, L/D Fix near-IR

Table S6. Patient treatment history. A table showing the pre-treatment history of patients whose whose CTC samples were successfully sequenced and included in the final scRNA-seq analysis during a combined [225Ac]Ac-PSMA-617 - [177Lu]Lu-PSMA-617 treatment.

Patient code	PSMA-RLT pretreatment	Chemoterapy received	Taxanes received	Platins received	Docetaxel received	Cabazitaxel received	Etoposide received	Xofigo received	ADT received	ADT	Denosumab received	Immunotherapy received	External Adjuvant Radiation received	Palliative External Radiation received	BRCA status	PARP inhibitor received	Surgery (total prostatectomy)
ALM60C8002	No	Yes	Yes	No	Yes	No	No	No	Yes	Leuprorelin acetate, Goserelin Acetate, Bicalutamide	Yes	NA	Yes	Yes	Negative	No	No
ALM63C9005	No	Yes	Yes	No	Yes	No	No	No	Yes	Bicalutamide, Goserelin Acetate, Leuprorelin, Abiraterone	No	NA	Yes	No	Negative	No	Yes
ALM69C5003	No	Yes	Yes	Yes	Yes	No	Yes	No	Yes	Enzalutamide	Yes	NA	Yes	Yes	Postive	Yes	Yes
ALM70C8006	No	Yes	Yes	No	Yes	No	No	No	Yes	Enzalutamide, Abiraterone	No	NA	Yes	No	Negative	Yes	No
ALM74C8008	No	No	No	No	No	No	No	No	Yes	Enzalutamide	Yes	NA	Yes	Yes	Negative	No	No
ALM76C6003	Yes	Yes	Yes	No	Yes	Yes	No	No	Yes	Enzalutamide, Abiraterone, Bicalutamid	No	NA	No	Yes	Negative	No	No
ALM77C5004	no	Yes	Yes	No	Yes	Yes	No	No	Yes	Enzalutamide	Yes	NA	Yes	No	Unknown	Yes	Yes
ALM77C5005	Yes	Yes	Yes	No	Yes	Yes	No	No	Yes	Enzalutamide, Abiraterone	Yes	NA	No	Yes	Negative	No	No
ALM78C7005	Yes	Yes	Yes	No	Yes	No	No	No	Yes	Abiraterone, Goserelin Acetate	No	NA	yes	yes	Unknown	No	No
ALM79C9003	Yes	Yes	Yes	No	Yes	Yes	No	No	Yes	Enzalutamide, Abiraterone, Leuprorelin acetate,	Yes	NA	No	No	Negative	No	No
ALM80C6007	Yes	Yes	Yes	No	Yes	Yes	No	No	Yes	Enzalutamide	No	NA	No	Yes	Negative	No	No
ALM80C7001	Yes	No	No	No	No	No	No	No	Yes	Enzalutamide, Bicalutamid	No	NA	No	No	Unknown	No	No
ALM80C8004	Yes	Yes	Yes	Yes	Yes	Yes	No	No	Yes	Enzalutamide, Abiraterone	No	NA	No	Yes	Negative	No	No
ALM81C2001	Yes	No	No	No	No	No	No	No	Yes	Bicalutamide, Buserelin Acetate, Abiraterone	No	NA	Yes	No	Unknown	No	No
ALM82C6002	Yes	Yes	Yes	No	Yes	Yes	No	No	Yes	Bicalutamide, Leuprorelin acetate, Flutamide	NA	No	Yes	Yes	Unknown	No	Yes
ALM82C7004	Yes	Yes	Yes	No	Yes	No	No	No	Yes	Unknown	NA	No	Yes	Yes	Unknown	No	Yes
ALM83C8001	No	Yes	Yes	Yes	Yes	No	No	No	Yes	Unknown	NA	No	No	Yes	Unknown	No	Yes
ALM88C3002	No	Yes	Yes	No	Yes	No	No	No	Yes	Unknown	NA	No	Yes	Yes	Unknown	No	Yes
LM66C3003	No	No	No	No	No	No	No	No	Yes	Unknown	NA	No	No	No	Unknown	No	Yes
LM67C6011	No	No	No	No	No	No	No	No	Yes	Unknown	NA	No	No	No	Unknown	No	No
LM68C6005	No	Yes	Yes	No	Yes	Yes	No	Yes	Yes	Unknown	NA	No	No	Yes	Unknown	No	No
LM75C7006	No	No	No	No	No	No	No	No	Yes	Unknown	NA	No	No	No	Unknown	Yes	No
LM81C6017	No	Yes	Yes	No	Yes	No	No	No	Yes	Unknown	NA	No	Yes	No	Unknown	No	No

Table S7. Primer sequence table. A table showing the sequences for the primers used for Tn5 based tagmentation.

Primer name	Sequence
**TSO**	5′-AAGCAGTGGTATCAACGCAGAGTACATrGrG+G-3′
**Tn5ME-A**	5′-TCGTCGGCAGCGTCAGATGTGTATAAGAGACAG-3′
**Tn5ME-B**	5′-GTCTCGTGGGCTCGGAGATGTGTATAAGAGACAG-3′
**Tn5RMErev**	5′-[phos]CTGTCTCTTATACACATCT-3′

Supplemental Methods

Single-cell RNA sequencing data analysis – read alignment and filtering

Data filtering:

We opted to be more lenient with the chosen MT gene expression cutoff for three main reasons:

a. We used both live-dead and apoptotic staining during cell sorting, which should ensure that dead and apoptotic cells were excluded from sequencing.
b. Ionizing radiation can affect the expression of various mitochondrial genes and metabolic processes, which might have an impact on radiation resistance [1,2].
c. Smart-Seq2 tends to generate a considerably higher proportion of mitochondrial reads than similar methods, such as 10X Genomics. This is most likely due to the more thorough organelle membrane disruption during the library preparation process [3]. This might be amplified in our case, as we used a higher-than-standard concentration of Triton X-100, to ensure proper cell lysis.

Supplemental References

1. Chaudhry, M.A., and Omaruddin, R.A. (2011). Mitochondrial gene expression in directly irradiated and nonirradiated bystander cells. Cancer Biother Radiopharm *26*, 657-663. 10.1089/cbr.2010.0940.
2. Kostyuk, S.V., Proskurnina, E.V., Konkova, M.S., Abramova, M.S., Kalianov, A.A., Ershova, E.S., Izhevskaya, V.L., Kutsev, S.I., and Veiko, N.N. (2021). Effect of Low-Dose Ionizing Radiation on the Expression of Mitochondria-Related Genes in Human Mesenchymal Stem Cells. Int J Mol Sci *23*. 10.3390/ijms23010261.
3. Wang, X., He, Y., Zhang, Q., Ren, X., and Zhang, Z. (2021). Direct Comparative Analyses of 10X Genomics Chromium and Smart-seq2. Genomics Proteomics Bioinformatics *19*, 253-266. 10.1016/j.gpb.2020.02.005.
