# Supplementary material for: EpCAM–PSMA: Potential predictors of treatment outcomes for PSMA-targeted alpha therapies in metastatic castration-resistant prostate cancer
Source: Mol Ther Oncol. 2026 Feb 2;34(1):201143. doi: 10.1016/j.omton.2026.201143 (PMC12925578; doi:10.1016/j.omton.2026.201143)
Supplement: Document S2. Article plus supplemental information [file mmc4.pdf]

Molecular Therapy
Oncology
Original Article

EpCAM–PSMA: Potential predictors of treatment outcomes for PSMA-targeted alpha therapies in metastatic castration-resistant prostate cancer

Gábor Bakos,[1] Ulrike Bauder-Wüst,[2] Jonathan Landry,[3] Mareike Roscher,[4] Beáta Ramasz,[5] Frank Bruchertseifer,[6] Alfred Morgenstern,[6] Clemens Kratochwil,[7] Vladimír Beneš,[3] and Martina Benešová-Schäfer[2]

[1]Research Group Molecular Biology of Systemic Radiotherapy, German Cancer Research Center (DKFZ), 69120 Heidelberg, Germany; [2]Research Group Translational Radiotheranostics, German Cancer Research Center (DKFZ), 69120 Heidelberg, Germany; [3]Genomics Core Facility, European Molecular Biology Laboratory (EMBL), 69117 Heidelberg, Germany; [4]Service Unit for Radiopharmaceuticals and Preclinical Studies, German Cancer Research Center (DKFZ), 69120 Heidelberg, Germany; [5]Flow Cytometry Core Facility, European Molecular Biology Laboratory (EMBL), 69117 Heidelberg, Germany; [6]European Commission, Joint Research Centre (JRC), 76125 Karlsruhe, Germany; [7]Department of Nuclear Medicine, University Hospital Heidelberg, 69120 Heidelberg, Germany

Targeted radionuclide therapy and targeted alpha therapy directed at prostate-specific membrane antigen (PSMA) represent emerging treatment modalities for metastatic castration-resistant prostate cancer (mCRPC). However, therapeutic resistance remains a significant barrier to their clinical success. We discovered that dynamic changes in cell surface levels of epithelial cell adhesion molecule (EpCAM) and PSMA can serve as predictive biomarkers in late-stage mCRPC patients treated with the beta-minus-particle-emitting [^{177}Lu]Lu-PSMA-617, in combination with the alpha-particle-emitting [^{225}Ac]Ac-PSMA-617, and we further explored the underlying molecular mechanisms. Using flow cytometry to profile EpCAM and PSMA on circulating tumor cells (CTCs), we observed that Nonresponders displayed significantly higher EpCAM and lower PSMA levels than Responders, both at baseline and after the first treatment cycle. Over subsequent cycles, both markers declined in Nonresponders, whereas Responder CTCs maintained EpCAM expression but progressively lost PSMA. Transcriptome analysis identified upregulation of hub genes involved in the regulation of key pathways such as enhanced DNA-damage repair, anti-apoptotic activity, increased tumor cell growth, and altered surface marker trafficking and recycling, potentially driving EpCAM-PSMA dynamics and contributing to therapy resistance. Ultimately, integrating surface-marker-driven treatment response predictions with novel treatment strategies may help to overcome treatment resistance in mCRPC.

INTRODUCTION

Targeted alpha therapy (TαT) is an emerging systemic treatment modality against prostate-specific membrane antigen (PSMA)-expressing prostate cancer (PCa) and is particularly promising for treating the metastatic forms of the disease.[1] TαT relies on a two-component drug, also called radiopharmaceutical, consisting of a PSMA-targeting component, and an alpha-particle-emitting radionuclide. These alpha-emitters deliver a highly localized, cytotoxic dose of ionizing radiation directly to cancer cells, minimizing damage to surrounding healthy tissue.[2] As PSMA is frequently overexpressed on the surface of PCa cells, including those in advanced stages, TαT has shown promising efficacy offering a novel, precision approach to combat aggressive PCa.[3,4] This modality is especially valuable for patients with limited treatment options, demonstrating significant potential in improving survival rates and quality of life.

Even though many patients react favorably to TαT, the ever-looming threat of therapy resistance is still present.[5] In order to develop potential countermeasures against TαT resistance and to efficiently utilize the limited ^{225}Ac supplies, an early treatment-response detection and a better understanding of treatment resistance are necessary. Liquid biopsies are known for enabling the detection of early signs of treatment resistance, monitoring tumor progression, and assessing minimal residual disease without the need for repeated, invasive tissue sampling.[6] This is particularly important in metastatic castration-resistant prostate cancer (mCRPC), where tracking genetic alterations or monitoring of relapse can significantly impact clinical decisions and personalized treatment plans. The ability to detect and analyze circulating tumor cells (CTCs) also allows for better assessment of therapeutic responses, making liquid biopsy a powerful tool for improving precision medicine and hence potentially enhancing patient outcomes.[7]

Therefore, we set out to explore various cell populations based on cell surface marker signals and to identify transcriptional changes in CTCs obtained from mCRPC patients undergoing PSMA-TαT.

Received 18 November 2025; accepted 28 January 2026;
https://doi.org/10.1016/j.omton.2026.201143.

Correspondence: Martina Benešová-Schäfer, Research Group Translational Radiotheranostics, German Cancer Research Center (DKFZ), 69120 Heidelberg, Germany.
E-mail: m.benesova@dkfz-heidelberg.de

© 2026 The Author(s).
Published by Elsevier Inc. on behalf of The American Society of Gene and Cell Therapy.
This is an open access article under the CC BY license (http://creativecommons.org/licenses/by/4.0/).

Our aim was to explore molecular markers, helpful in the early detection of treatment outcomes, and to shed light on potential mechanisms leading or contributing to treatment resistance. In order to carry out this study, we selected patients with late-stage mCRPC currently undergoing a targeted radionuclide therapy (TRNT) combination with [^{177}Lu]Lu-PSMA-617 and [^{225}Ac]Ac-PSMA-617, for peripheral blood collection (Figure S1). This allowed us to isolate CTCs and analyze changes in the levels of selected cell surface markers—namely epithelial cell adhesion molecule (EpCAM) and PSMA—as well as general transcription levels of various genes upon PSMA-TαT by utilizing flow cytometry and single-cell mRNA sequencing. It also allowed us to compare these changes among responding and non-responding patients, referred to as Responders and Nonresponders, respectively, in the hope of finding molecular markers useful in predicting treatment outcomes and explaining potential mechanisms behind the different treatment response.

RESULTS

Patient pre-classification

We set out to collect and store CTC-containing liquid biopsy samples from heavily pre-treated patients undergoing PSMA-TRNT/-TαT using a combination of [^{177}Lu]Lu-PSMA-617 and [^{225}Ac]Ac-PSMA-617. In order to minimize the potential batch effect and experimental bias during downstream sample processing, we opted to pre-classify the patients before CTC isolation and divide them into two patient groups based on their treatment response: Responders and Nonresponders. The pre-classification was done by a trained and treating physician, and it was based on the trajectory of the patient's overall prostate-specific antigen (PSA) levels throughout the treatment course (Table S1), combined with positron emission tomography (PET) and/or single photon emission computed tomography (SPECT) imaging data, if available. Although clinical treatment response designation defines more than two outcomes,[8,9] we opted to classify both partial (PR) and complete response (CR) as response, while both stable (SD) and progressive disease (PD) as non-response, despite the fact that SD may represent clinical benefit in late-stage disease. Overall, out of the 66 patients (143 total samples), we classified 28 as overall Responders (74 total samples) and 16 as overall Nonresponders (43 total samples). Twenty two patients (28 total samples) were excluded from the analysis, as their response status could not be determined.

Buffy coat and subsequent CTC isolation

The liquid biopsy samples were collected right before each scheduled treatment cycle, together with the standard blood samples for biomarker testing. The described collection approach was designed to minimize harm to the patients and to avoid the need to process radioactive blood samples. To minimize potential batch effect and experimental bias, we adopted a previously described procedure to freeze CTCs concentrated in the buffy coat layer of whole blood[10] until downstream processing. To further tailor the protocol to our needs, we adjusted the freezing and thawing procedures using healthy whole blood samples spiked with PSMA^{+}EpCAM^{+} C4-2 cells. After optimization, we achieved an over 90% survival rate among C4-2 cells following buffy coat isolation, freezing, and thawing, confirmed by crystal violet staining and subsequent cell counting under light microscope (data not shown).

Next, we set out to isolate the CTCs found in cryopreserved buffy coat samples of each pre-classified patient and to monitor the potential difference in CTC numbers between the response groups. In order to effectively isolate CTCs, we devised a gating strategy involving two exclusion gates to filter out any apoptotic or dead cells, and any potential leukocytes remaining after depletion, and a sort gate that served to capture CTCs expressing two surface markers: EpCAM, a commonly used CTC marker,[11–13] and PSMA, a specific marker found on the surface of PCa cells,[14,15] which serves as the target for radiopharmaceuticals.[16–18] We reasoned that, by using both markers we can maximize sort efficiency by capturing three hypothetical CTC populations: EpCAM^{+}PSMA^{-}, EpCAM^{+}PSMA^{+}, and EpCAM^{-}PSMA^{+} (Figures S2 and S3). It is important to note that, although EpCAM/PSMA-based staining may underrepresent certain CTC populations at different stages of epithelial-to-mesenchymal transition (EMT),[19–21] this strategy was deliberately chosen to interrogate EpCAM/PSMA-expressing CTC subsets that are most relevant to PSMA-TRNT. Indeed, by using the described staining and gating strategy, we isolated a total of 436 CTCs (192 from Responders and 244 from Nonresponders) from 117 samples obtained from 46 patients. As not every sample yielded viable CTCs, the final number of patients (17 Responders, 12 Nonresponders) and samples (74) were reduced after sorting. Therefore, this study is exploratory and not powered for definitive statistical inference. Nonetheless, we observed cells from all three hypothesized subpopulations based on their surface marker status.

Surface marker profile of isolated CTC populations

To search for overall trends in surface marker status among the response groups and to gain insight into how PSMA-TαT affects the chosen surface markers and the isolated CTC populations, we conducted an in-depth analysis of the obtained FACS data.

First, we performed a general assessment of the obtained cell numbers, to explore potential differences between response groups. Following data normalization and filtering (Figure S4), the final cell number used for data analysis was 422 from a total of 54 samples. We observed a higher total cell number in the Nonresponder samples (n = 241) compared to samples from Responders (n = 181) (Figure 1A). Additionally, the median sorted CTC number in the Nonresponder group (8, range: 1–44, n = 21) was significantly higher (p value = 0.0234, Mann-Whitney U test) than in the Responder group (2, range: 1–24, n = 33) (Figure 1B). This suggests that Nonresponders have a higher number of CTCs compared to Responders.

Next, we explored how cells from the overall response groups are distributed among the three sorted CTC subpopulations (EpCAM^{+}PSMA^{-}, EpCAM^{-}PSMA^{+}, and EPCAM^{+}PSMA^{+}). To define these subpopulations, we used the minimum median

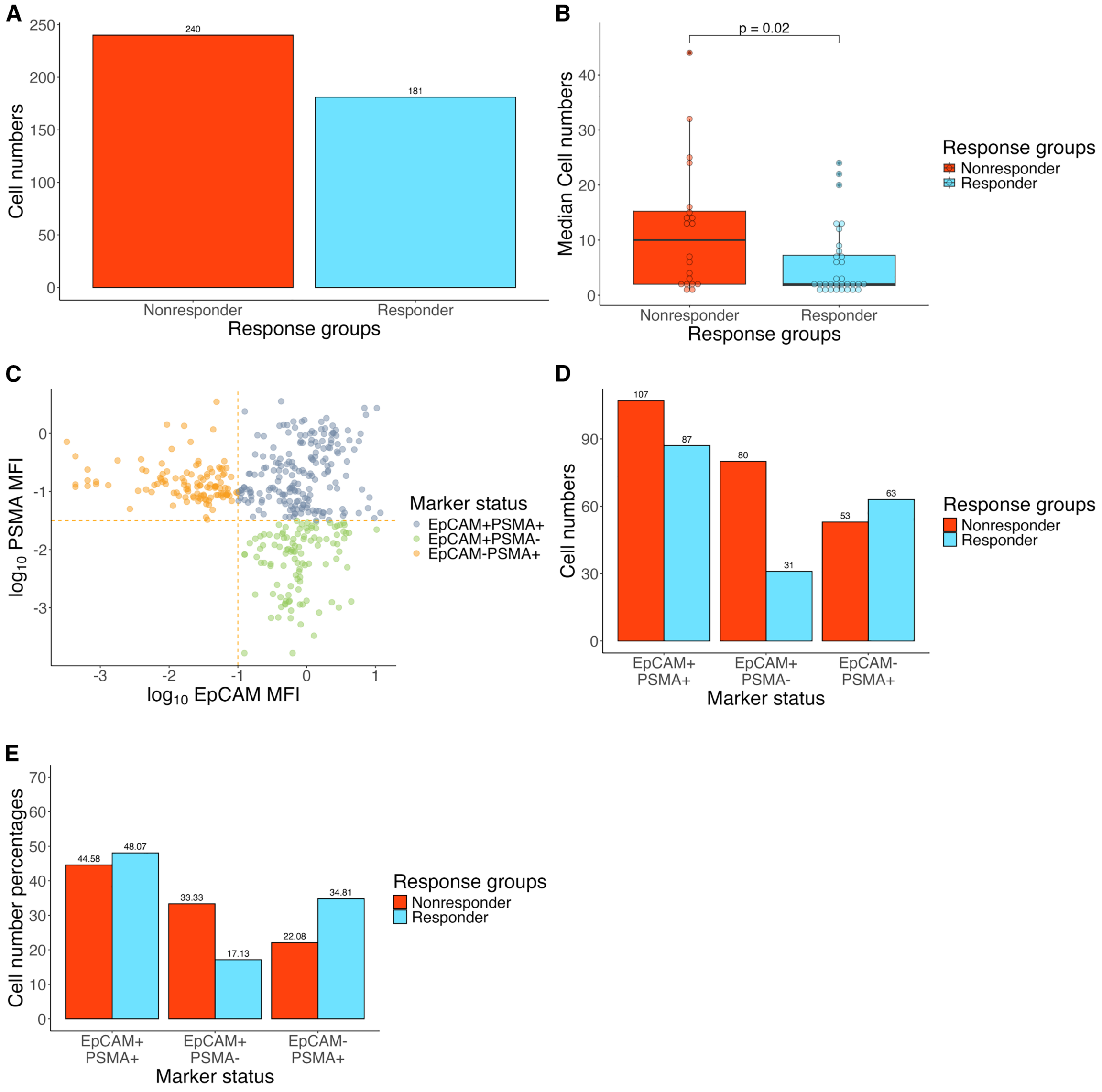

Figure 1. CTC numbers and surface marker distribution

(A) A bar chart showing the total sorted CTC cell numbers after data normalization and filtering. (B) A boxplot showing the median sorted CTC numbers per response group. The p value (0.02) was calculated using the Mann-Whitney U test. (C) A scatterplot showing the CTC gating strategy and the resulting CTC subpopulations. The yellow dotted line represents the thresholds used to distinguish between various CTC subpopulations, based on their EpCAM and PSMA MFI levels. Note that the sort gate encompassed all three subpopulations. (D) A bar chart showing the distribution of sorted CTCs between the three subpopulations based on EpCAM and PSMA. The chart shows the raw cell numbers in each sub-population. (E) A bar chart showing the distribution of sorted CTCs between the three subpopulations. The chart shows the percentage-based distribution of CTCs from Responder and Nonresponder patients among each sub-population.

fluorescent intensity (MFI) values to set thresholds on both EpCAM and PSMA channels (Figure 1C). To see, if there are any differences between the distribution of Responder and Nonresponder cells in the various surface marker subpopulations, we looked at both the raw cell counts and the proportional distribution of the two response groups among the subpopulations. We expected to see a close to equal distribution of the two response groups in all three surface marker subpopulations. While the percentage of Responder and Nonresponder cells in the $EpCAM^{+}PSMA^{+}$ subpopulation was quite close to equal, we observed a skewed distribution in the $EpCAM^{+}PSMA^{-}$ and $EpCAM^{-}PSMA^{+}$ subpopulations (Figures 1D and 1E). This suggests that, in general, Nonresponders have a higher number of $EpCAM^{+}PSMA^{-}$ CTCs compared to Responders, while Responders have a higher number $EpCAM^{-}PSMA^{+}$ CTCs compared to the Nonresponders.

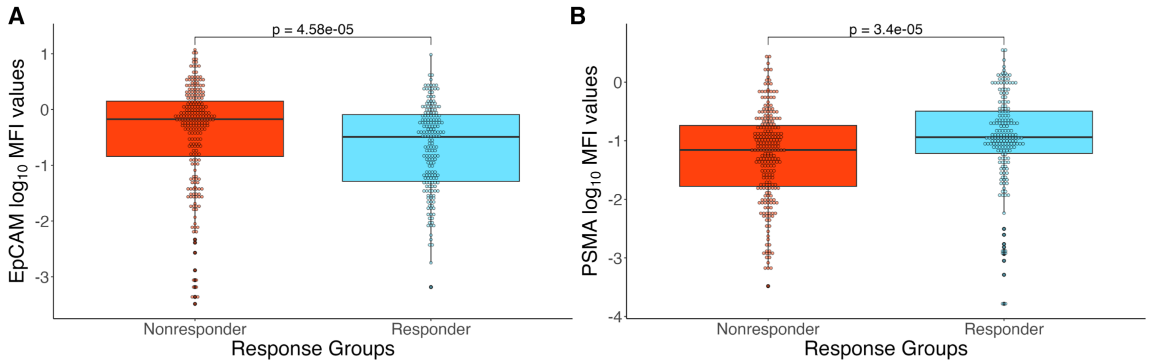

Figure 2. Surface marker comparison between response groups

(A) A boxplot showing the comparison of EpCAM MFI values on CTCs sorted from Responder and Nonresponder patients. The *p* value (4.58e−05) was calculated using the Mann-Whitney U test. (B) A boxplot showing the comparison of PSMA MFI values on CTCs sorted from Responder and Nonresponder patients. The *p* value (3.43e−05) was calculated using the Mann-Whitney U test.

This skewed distribution piqued our interest, as this could suggest a generally lower PSMA expression in CTCs found in Nonresponder samples vs. CTCs found in Responder samples. To gain a more granular insight into the differences among the observed cell populations, we further explored the general surface marker levels between Responder and Nonresponder CTCs. Our results showed that the median EpCAM MFI value among the CTCs from Nonresponders ($\log_{10}$median-MFI = −0.173, range: −3.36–1.07) was significantly higher (*p* value = 4.58e−05, Mann-Whitney U test) than among CTCs from Responders ($\log_{10}$median-MFI = −0.490, range: −3.36–0.983) (Figure 2A). As for the PSMA channel, we observed the opposite, where the median MFI value among the CTCs from the Nonresponders ($\log_{10}$median-MFI = −1.160, range: −3.481–0.438), was significantly lower (*p* value = 3.43e−05, Mann-Whitney U test) than among CTCs from Responders ($\log_{10}$median-MFI = −0.940, range: −3.297–0.553) (Figure 2B). These data reinforce our previous observation and suggests that CTCs sorted from Nonresponders have higher EpCAM and lower PSMA surface levels than CTCs sorted from Responders.

Dynamic surface marker changes in various CTC populations

Aggregate MFI values provided valuable information regarding overall differences in EpCAM and PSMA surface levels between Responder and Nonresponder CTC populations. However, because these values were pooled across all treatment cycles, they lacked the temporal resolution necessary to assess dynamic, longitudinal changes in surface marker expression during therapy. Therefore, to investigate potential treatment-associated dynamics in EpCAM and PSMA surface levels, we performed a cycle-to-cycle analysis of median MFI values both within and between response groups.

To this end, first we looked at the number of sorted CTCs per treatment cycle for both Responder and Nonresponder samples. As cycle 4 had a low number of sorted cells (Responders: 3; Nonresponders: 0), we made a cutoff at the 3rd treatment cycle for both response groups. Then we compared the log-transformed MFI values of CTCs from each treatment cycle to the 0-time point (baseline cycle, blood collected shortly before the first PSMA-TαT cycle was administered), to see if there were any changes in the EpCAM and PSMA levels throughout the treatment. In the case of CTCs sorted from Responder samples, we observed a relatively flat trajectory with no significant difference in the EpCAM MFI values in cycles 1–3, compared to the 0-time point (Figure 3A). In the case of PSMA, the results showed a decreasing trajectory with a significant decrease in the median MFI values at cycle 2 (0-time-point median MFI: 0.1920; cycle 2 median MFI: 0.0453; *p* value = 2.11e−05, Mann-Whitney U test) and cycle 3 (0-time-point median MFI: 0.1920; cycle 3 median MFI: 0.0186; *p* value = 3.54e−05, Mann-Whitney U test) compared to the 0-time point (Figure 3B). As for CTCs sorted from Nonresponder samples, the EpCAM MFI values show a declining trend, with a significant decrease at cycle 2 (0-time-point median MFI: 0.8258; cycle 2 median MFI: 0.2872; *p* value = 0.0002, Mann-Whitney U test) and at cycle 3 (0-time-point median MFI: 0.8258; cycle 3 median MFI: 0.2706; *p* value = 0.0001, Mann-Whitney U test) compared to the 0-time point (Figure 3C). In the case of PSMA, we also observed a decline over time with a significant decrease in median MFI values at cycle 3 (*p* value = 6.91e−06, Mann-Whitney U test) compared to the 0-time point (Figure 3D). When compared to CTCs from Nonresponders, the median EpCAM MFI values were significantly lower on CTCs from Responders at 0-time point (Nonresponder-median-MFI: 0.8258; Responder median MFI: 0.3299; *p* value = 4.46e−06, Mann-Whitney U test) and at cycle 1 (Nonresponder-median-MFI: 0.8849; Responder-median-MFI: 0.0992; *p* value = 1.43e−05, Mann-Whitney U test). However, this difference disappeared at later cycles (Figure 3E). In the case of PSMA, although CTCs from both Responder and Nonresponder samples show the same declining trend, cells from the Nonresponder samples show a significantly lower median MFI value at 0-time point (Nonresponder-median-MFI: 0.0683; Responder-median-MFI: 0.1920; *p* value = 0.0002, Mann-Whitney U test) and at cycle 1

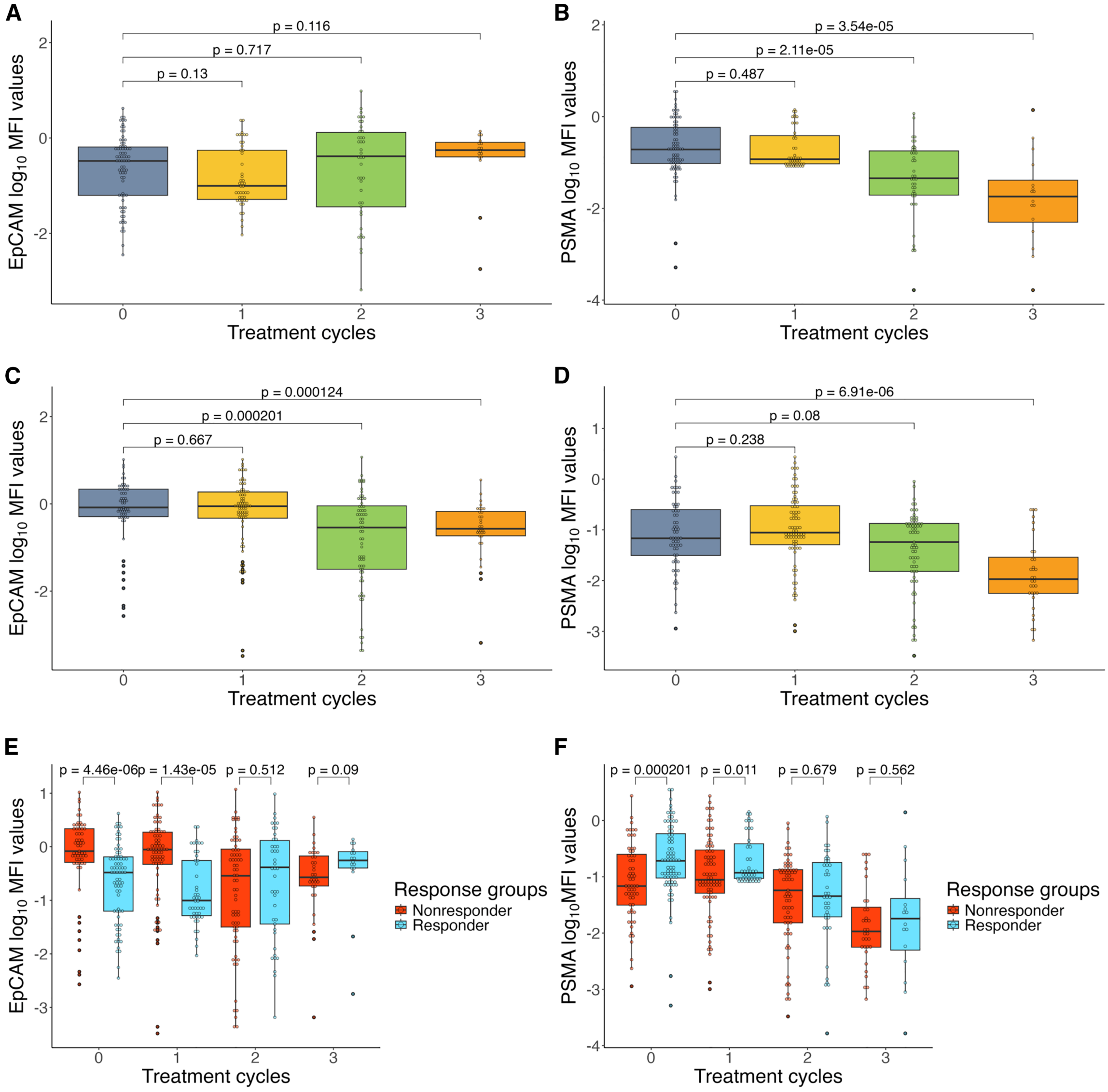

Figure 3. EpCAM and PSMA surface level over the treatment cycles

(A) A boxplot showing the changes in EpCAM surface marker levels on CTCs from Responder patients, from cycle 0 to cycle 3. The *p*-values were calculated using the Mann-Whitney-U-test with a significance level of $p < 0.05$ for this and all subsequent panels. (B) A boxplot showing the changes in PSMA surface marker levels on CTCs from Responder patients, from cycle 0 to cycle 3. (C) A boxplot showing the changes in EpCAM surface marker levels on CTCs from Nonresponder patients, from cycle 0 to cycle 3. (D) A boxplot showing the changes in PSMA surface marker levels on CTCs from Nonresponder patients, from cycle 0 to cycle 3. (E) A boxplot showing the changes in EpCAM surface marker levels on CTCs from both Responder and Nonresponder patients, from cycle 0 to cycle 3. *p* values represent the difference in EpCAM surface marker levels on CTCs from the two response groups in any given cycle. (F) A boxplot showing the changes in PSMA surface marker levels on CTCs from both Responder and Nonresponder patients, from cycle 0 to cycle 3. *p* values represent the difference in EpCAM surface marker levels on CTCs from the two response groups in any given cycle.

(Nonresponder-median-MFI: 0.0883; Responder-median-MFI: 0.1181; *p* value = 0.0110, Mann-Whitney U test) compared to cells from Responder samples. Together, these results suggest that CTCs from Responder samples start with relatively lower EpCAM levels than their Nonresponder counterparts, which stays mostly stable throughout the treatment. Cells form Nonresponders seem to have higher levels of EpCAM at baseline, which shows a decrease over the treatment, most prominently after the first treatment cycle.

As for PSMA, cells from Responder samples seem to possess higher levels at baseline; however, this quickly decreases throughout the treatment. A similar decrease in surface PSMA levels can be observed in the cells from Nonresponder samples, in addition to their lower baseline PSMA. The progressive loss of PSMA expression in Responder CTCs may reflect therapy-induced antigen modulation or selective elimination of PSMA-high clones, a phenomenon previously reported for TRNT.

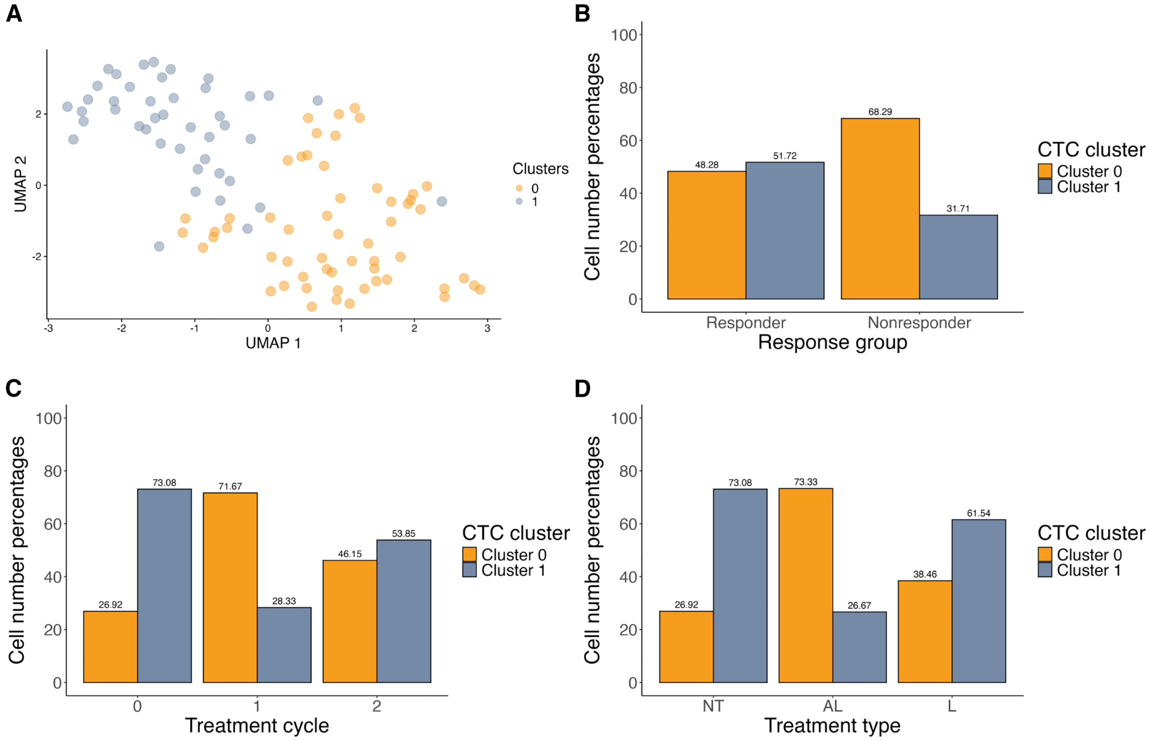

Figure 4. CTC clusters and their correlation to various treatment variables

(A) A uniform manifold approximation and projection (UMAP) plot showing the clustering of CTCs based on their gene expression pattern. (B) A bar chart showing the distribution of CTCs between clusters 0 and 1, based on patient response status. (C) A bar chart showing the distribution of CTCs between clusters 0 and 1 based on the treatment cycles they originate from. (D) A bar chart showing the distribution of CTCs between clusters 0 and 1 based on the treatment type the patient received. NT = no treatment, AL = [^{225}Ac]Ac-PSMA-617-[^{177}Lu]Lu-PSMA-617, L = [^{177}Lu]Lu-PSMA-617.

Exploring the CTC transcriptome: Library preparation and data filtering

The observed dynamic changes in the surface marker levels appeared to have a good potential to predict treatment outcomes. Therefore, we opted to explore molecular mechanisms that underpin both the observed EpCAM-PSMA dynamics and treatment resistance. To this end, we examined the transcriptome of the sorted CTCs by single-cell RNA sequencing (scRNA-seq).

As a first step, we prepared amplified cDNA libraries from the sorted cells, using a Smart-seq2-based approach.[22] During the library preparation, we noticed that many of the cells produced low-concentration, low-quality, partially degraded RNA. Therefore, we conducted rigorous quality checks by running the cDNA from every cell—following both reverse transcription and tagmentation—on a fragment analyzer. Out of the 436 sorted cells, 31.6% (138 cells total; Responders: baseline, 36 cells; cycle 1, 31 cells; cycle 2, 11 cells; Nonresponders: baseline, 15 cells; cycle 1, 38 cells; cycle 2, 7 cells) passed the quality controls and were moved on to sequencing (Figure S5).

Next, we set out to prepare the resulting sequencing data for the downstream analysis. After filtering, we were left with 99 cells in total (41 cells from Nonresponder and 58 cells from Responder samples) (Figure S6A). In these cells, the median number of detected transcripts was 4,018,895 (range: 4,439–17,336,862) and the median number of detected genes was 3,326 (range: 1,013–7,314) while the median mitochondrial (MT) gene expression percentage was 16.4% (range: 0.1–74.6) (Figures S6B–S6E).

Exploring the CTC transcriptome: Clustering and gene expression analysis

Next, we set out to differentiate various CTC populations by clustering analysis. We performed unsupervised clustering analysis, then visualized the results by a uniform manifold approximation and projection (UMAP) plot (Figure 4A). UMAP clustering showed two distinct clusters among the analyzed CTCs.

To determine if the observed clustering behavior was dependent on the overall treatment response, we explored the relationship between the clusters and response groups (Figure 4B). The clustering behavior showed no significant dependence on the overall treatment response (p value = 0.0761, chi-squared test of independence). Therefore, we looked at how other variables—such as the treatment cycle, the cell-cycle phase, treatment type, and surface marker status—influence the clustering behavior of CTCs. Out of these tested

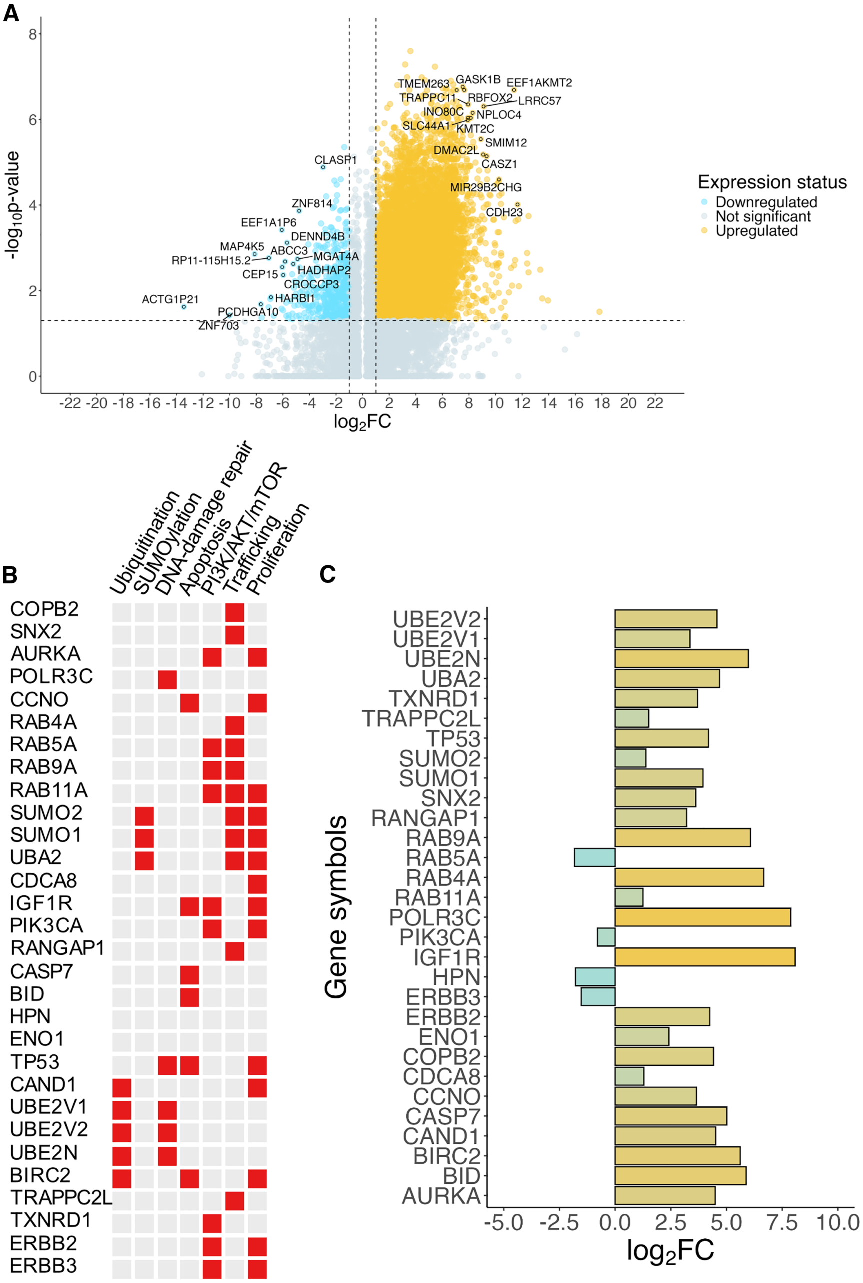

Figure 5. Differential gene expression and selected hub genes

(A) A volcano plot showing the differentially expressed genes (DEGs) between cluster 0 vs. cluster 1. The top 15 DEGs based on ranking (log$_2$FC * p-adjusted value) are highlighted with gene symbols. (B) A heatmap showing the processes the selected hub genes are directly involved in. (C) A bar chart showing the relative expression level of the selected hub genes in cluster 0 vs. cluster 1.

variables, treatment cycle and with that, overall treatment status (p value = 0.0004, chi-squared test of independence) and treatment type (p value = 0.0356, chi-squared test of independence) showed a significant influence on the clustering (Figures 4C, 4D, and S7A–S7C). Overall, this suggests that cluster 0 is mostly composed of cells subjected to treatment with [^{225}Ac]Ac-PSMA-617 alongside [^{177}Lu]Lu-PSMA-617, collected during treatment cycles 1 or 2. In contrast, cluster 1 is mostly composed of cells not subjected to treatment—collected before the first treatment cycle (baseline)—or exposed to treatment with [^{177}Lu]Lu-PSMA-617—collected before the patient received [^{225}Ac]Ac-PSMA-617 alongside [^{177}Lu]Lu-PSMA-617.

After obtaining some insight about the composition of the clusters, we opted to explore the potential gene expression differences, most likely attributing to the combination treatment with [^{225}Ac]Ac-PSMA-617. To this end, we conducted a differential gene expression (DGE) analysis, which identified 8,544 genes as significantly differentially expressed between the two clusters with a p-adjusted value of <0.05 (Bonferroni correction) (Table S2). To refine our results, we implemented further filtering based on expression level (log2FC), selecting only those genes with at least a 2-fold difference in expression (log2FC ≤ -1 or log2FC $\geq$ 1). After the applied filtering, we were left with a total of 7,630 significantly differentially expressed genes (DEGs). Out of these, 7,176 genes were upregulated in cluster 0 vs. cluster 1, while 454 genes were downregulated in cluster 0 compared to cluster 1 (Figure 5A).

To further explore the processes and pathways these DEGs are involved in, we followed-up the DEG analysis with an integrated gene set enrichment analysis (irGSEA).[23] Using the MSigDB database as a reference to explore impacted pathways, we ran the analysis against multiple reference gene sets containing both higher level (Hallmark, Biocarta, and GO:Biological) and more detailed pathway information (Processes and REACTOME). The results showed that multiple key cellular processes are differentially regulated between the two clusters, including DNA-damage repair (DDR), apoptosis, cellular trafficking, and cell growth (Figures S8 and S9).

To elucidate how these pathways are impacted by the treatment, we opted to explore the key associated genes and their expression status in the clusters. We conducted a hub gene analysis using the irGSEA.hub function, which after thresholding identified 30 hub

genes (Table S3), directly involved in ubiquitination, SUMOylation, cellular trafficking, and the regulation of the PI3K/AKT/mTORC pathway. Most of the hub genes were upregulated in cluster 0 compared to cluster 1, with a few notable exceptions (RAB5A, PIK3CA, HPN, and ERBB3) (Figures 5B and 5C). Taken together, the results indicate that CTCs from later treatment cycles show enhanced DDR and reduced apoptosis. Furthermore, they seem to have decreased clathrin-dependent endocytosis and perturbed anterograde and retrograde trafficking. Finally, they seem to exhibit increased SUMOylation and a positive modulation of the PI3K/AKT/mTORC pathway.

DISCUSSION

Since PCa incidence is projected to rise substantially in high-income countries over the coming decades,[24] the importance of efficient treatment modalities and predictive diagnostic tools will increase in the coming years. PSMA-TαT shows promise as an effective treatment option for late- and potentially early-stage mCRPC patients, who have developed resistance to other therapies, offering a median overall survival of 15 months.[25] However, therapy resistance remains a significant challenge, often resulting in poor treatment outcomes. To identify markers capable of predicting treatment outcomes and uncover mechanisms that might lead to or contribute to therapy resistance, we collected samples from a limited number of patients with diverse pre-treatment backgrounds who underwent combined [^{225}Ac]Ac-PSMA-617 and [^{117}Lu]Lu-PSMA-617 therapy (Table S6). Although this increases the complexity of the obtained data, we believe it is important to reflect the current clinical reality and explore the potential resistance mechanisms under frequently used treatment schedules and pre-treatment conditions. The obtained samples were processed by flow cytometry and single-cell RNA sequencing.

Single-cell sorting data revealed significant differences in EpCAM and PSMA surface levels on CTCs from Responders versus Nonresponders. Specifically, CTCs from Nonresponders showed a significantly higher median EpCAM and a significantly lower median PSMA level than CTCs from Responders (Figures 2A and 2B). Furthermore, by analyzing marker levels across treatment cycles, we found that this difference was already present before treatment initiation (Figures 3E and 3F). During treatment, surface levels of both EpCAM and PSMA declined in Nonresponder CTCs, whereas Responder CTCs showed relatively stable EpCAM and decreasing PSMA levels (Figures 3A–3D). Although it would stand to reason that this decline is connected to the downregulation of these markers, differential gene expression analysis revealed no significant differences between the response groups. This indicates that the surface levels of these markers are regulated at the translational or posttranslational, rather than the transcription, level. Interestingly, the differences in cell surface markers between the two response groups diminished over the course of treatment and disappeared by later cycles (Figures 3E and 3F). This suggests that treatment with [^{225}Ac]Ac-PSMA-617 induces a similar adaptation or selection process in CTCs from both response groups. This means that as the treatment progresses, CTCs with higher PSMA and lower EpCAM levels are effectively eliminated in both patient groups. Therefore, we end up isolating the surviving cells that might display similar gene expression patterns. This notion is supported by the obtained scRNA-seq data, where the clusters obtained by unsupervised clustering analysis (Figure 4A) correlated primarily with the treatment status (non-treated, cycle 0; treated, cycle 1–2) and treatment type ([^{177}Lu]Lu-PSMA-617 alone or [^{177}Lu]Lu-PSMA-617 combined with [^{225}Ac]Ac-PSMA-617). Notably, these clusters were less influenced by other parameters such as overall response status (Figures 4B–4D; Figure S6). Nonetheless the combination of lower PSMA and higher EpCAM levels on CTCs, along with increased detected CTC numbers, appears to correlate with treatment resistance. This observation may offer a valuable complementary diagnostic tool for predicting treatment outcomes either before (baseline) or during early treatment cycles (cycles 1 and 2). Given the fact that the observed EpCAM-PSMA dynamics was detected using flow cytometry, our depletion and staining protocol combined with the existing or potentially improved analysis script can offer an easy to adopt method for clinical laboratories. Such tools could be particularly relevant in patient stratification if [^{225}Ac]Ac-PSMA-617 therapy becomes widely adopted—especially as a third- or second-line treatment option—given current constraints in the ^{225}Ac supply.

As for the molecular pathways potentially underpinning both the observed EpCAM-PSMA dynamics and potential treatment resistance, transcriptome analysis revealed significant changes in the expression of various hub genes directly involved in ubiquitination, SUMOylation, DDR, regulation of the PI3K/AKT/mTOR pathway, proliferation control, and most importantly, cellular trafficking (Figure 5B; Table S3). Most of these hub genes were upregulated in response to [^{225}Ac]Ac-PSMA-617 treatment apart from a few notable exceptions (RAB5A, PIK3CA, HPN, and ERBB3), prompting the modulation of key survival processes.

Among the hub genes upregulated upon PSMA-TαT, several may explain the observed surface marker dynamics and highlight mechanisms leading to treatment resistance. For example, we identified upregulated genes such as UBE2N, UBE2V1, and UBE2V2, ubiquitin-conjugating enzymes essential for DSB repair,[26–29] as well as POLR3C, a subunit of RNA polymerase III involved in homologous recombination repair of DSBs.[30] This represents a key survival response to the DNA damage induced by alpha radiation. Upregulation of anti-apoptotic regulators further highlights the adaptive survival mechanisms in TαT-treated CTCs. BIRC2, an inhibitor of apoptosis involved in the ubiquitination and degradation of caspase-3 and -7,[31] and IGF1R, which supports cell survival by activating the AKT pathway,[32,33] were upregulated upon treatment. Significant alterations in cellular trafficking pathways were also observed in TαT-treated CTCs, with the upregulation of key regulators such as COPB2. COPB2, a member of the COPI complex and an important regulator of Golgi-to-ER retrograde trafficking, regulates the nuclear trafficking of receptor tyrosine kinases (RTKs) such as ERBB2.[34] Similarly, members of the RAB family, which is crucial

for intracellular trafficking, were also upregulated. RAB4A, located in early and recycling endosomes, promotes the rapid recycling of integrins, EGFR, and other surface proteins, enhancing proliferation, invasiveness, and metastasis formation.[35–38] RAB11A, a key player in the slow recycling pathway, may disrupt normal trafficking of internalized PSMA (FOLH1), increasing retention in recycling endosomes (RE). This mirrors the behavior observed for transferrin,[38,39] which shares remarkably similar trafficking dynamics with PSMA.[40] The upregulation of TRAPPC2L, a TRAPPII/III complex adaptor with guanosine exchange factor (GEF) activity, may further enhance RAB11 activity and increase COPI and COPII vesicle trafficking, thereby affecting ER-to-Golgi and Golgi-to-ER transport.[41,42]

Conversely, we also identified hub genes with reduced expression upon PSMA-TαT. Among these was RAB5A, another member of the RAB family found in early endosomes. RAB5A is primarily responsible for cargo sequestration and participates in the clathrin-mediated endocytosis of transferrin and G-protein-coupled receptors.[37,43] Its downregulation aligns with previous observations in advanced PCa.[44] Similarly, PIK3CA, the catalytic subunit of phosphoinositide-3-kinase (PI3K), was downregulated. This gene is commonly overexpressed and reported to more frequently harbor activating rather than non-activating mutations in PCa.[45,46] It plays a role in activating the PI3K/AKT/mTOR pathway, which promotes cell survival, growth and cell-cycle progression, and is upstream of AKT.[47] Notably, AKT can still be activated independently of PI3K by various kinases such as TBK1, found to be upregulated in cells from cluster 0.[47–49] Lastly, ERBB3, an RTK connected to PI3K/AKT and AR signaling in PCa,[50] was also downregulated. While ERBB3 typically requires ERBB2 as a dimerization partner for AKT activation, ERBB2 alone can activate the PI3K/AKT pathway when overexpressed.[51]

Overall, these transcriptional changes may represent key survival and adaptation mechanisms in CTCs. They include enhanced DSB repair activity, suppression of apoptotic pathways, and modulation of the trafficking and surface levels of critical proteins such as PSMA, ERBB2, and IGF1R.

Based on these findings, we propose a model to explain the observed surface marker dynamics on CTCs—and potentially on solid metastatic sites—as well as the mechanisms that may contribute to treatment resistance in patients with advanced mCRPC. In this model, mCRPC patients harbor multiple populations of CTCs and likely solid metastatic cells as CTCs stem from solid tumor sites. These populations can be distinguished by a detectable difference in their surface EpCAM and PSMA levels likely linked to the differential expression of genes responsible for cellular trafficking (e.g., COPB2, TRAPPC2L, SUMO1, SUMO2, and UBA2) and recycling (e.g., RAB4A and RAB11A). Additionally, differences in surface marker expression are accompanied by a distinct gene expression pattern, termed the "Cluster 0" (C0) pattern, which promotes CTC survival. This pattern is characterized by increased DSB repair activity (e.g., UBE2N, UBE2V1, UBE2V2, and POLR3C), elevated anti-apoptotic activity (e.g., BIRC2, IGF1R, and CCNO), and enhanced growth and proliferation, driven primarily by the PI3K/AKT/mTOR pathway. Therefore, CTCs and potentially other tumor cells with lower PSMA surface levels tend to be targeted less effectively by [^{225}Ac]Ac-PSMA-617, resist damage more efficiently, and retain the ability to proliferate even under therapeutic pressure. Over time, this results in the selection and enrichment of such CTCs—and potentially PSMA-low solid tumor cells—in treated patients, ultimately leading to reduced treatment efficacy and resistance. Our model suggests that patients who develop treatment resistance earlier possess a larger population of PSMA-low, EpCAM-high, C0-pattern CTCs and potentially solid tumor cells, even before [^{225}Ac]Ac-PSMA-617 treatment begins. This pre-existing cellular heterogeneity could explain the observed reduction in treatment efficacy and poorer clinical outcomes in resistant cases. As the PSMA-low, EpCAM-high, C0-pattern CTC population is not unique to Nonresponders, early detection of treatment resistance is extremely important, as it can offer physicians the time to develop alternative treatment plans and regimes such as the use of combination therapies, using DDR inhibitors, RTK inhibitors, and AKT inhibitors.

Nonetheless, it is important to note that our model is based on data obtained from CTCs, making it challenging to extrapolate findings to solid tumor sites due to their vastly different microenvironments. This might also help to explain the discrepancy with previously reported clinical observations,[5] where resistant patients show strong PSMA expression in solid tumor sites even after multiple treatment cycles (Figure 6). While the relationship between PSMA levels expressed on the tumor cells necessary for diagnostic imaging and those sufficient for effective therapeutic targeting remains poorly understood, it is likely that additional resistance mechanisms independent of targeting evasion are at play and warrant further investigation. Moreover, we cannot exclude preferential loss of fragile CTC subsets, although this limitation applies uniformly across the cohort.

Ultimately, our results suggest the presence of various CTC populations based on their surface EpCAM and PSMA levels, in mCRPC patients. Among these populations, we identified two, which seems to correspond well with the development of resistance to [^{225}Ac]Ac-PSMA-617. The presence of EpCAM-high and PSMA-low population seems to be dominant in patients with poor therapy response, while the presence of EpCAM-low and PSMA-high population seems to be dominant in patients with favorable therapy response even before the first round of treatment. These observations, although from a limited cohort, suggests that the observed EpCAM-PSMA dynamics could be used as a powerful complementary diagnostic tool, in predicting resistance to PSMA-TαT. This gives us an exciting opportunity to further explore these dynamics and develop robust prediction algorithms using large patient cohorts.

MATERIALS AND METHODS

Patient selection and treatment

[^{225}Ac]Ac-PSMA-617 and [^{177}Lu]Lu-PSMA-617 were prepared for injection as described before[52,53] and administered as a compassionate use in adherence to paragraph 37, "Unproven Interventions

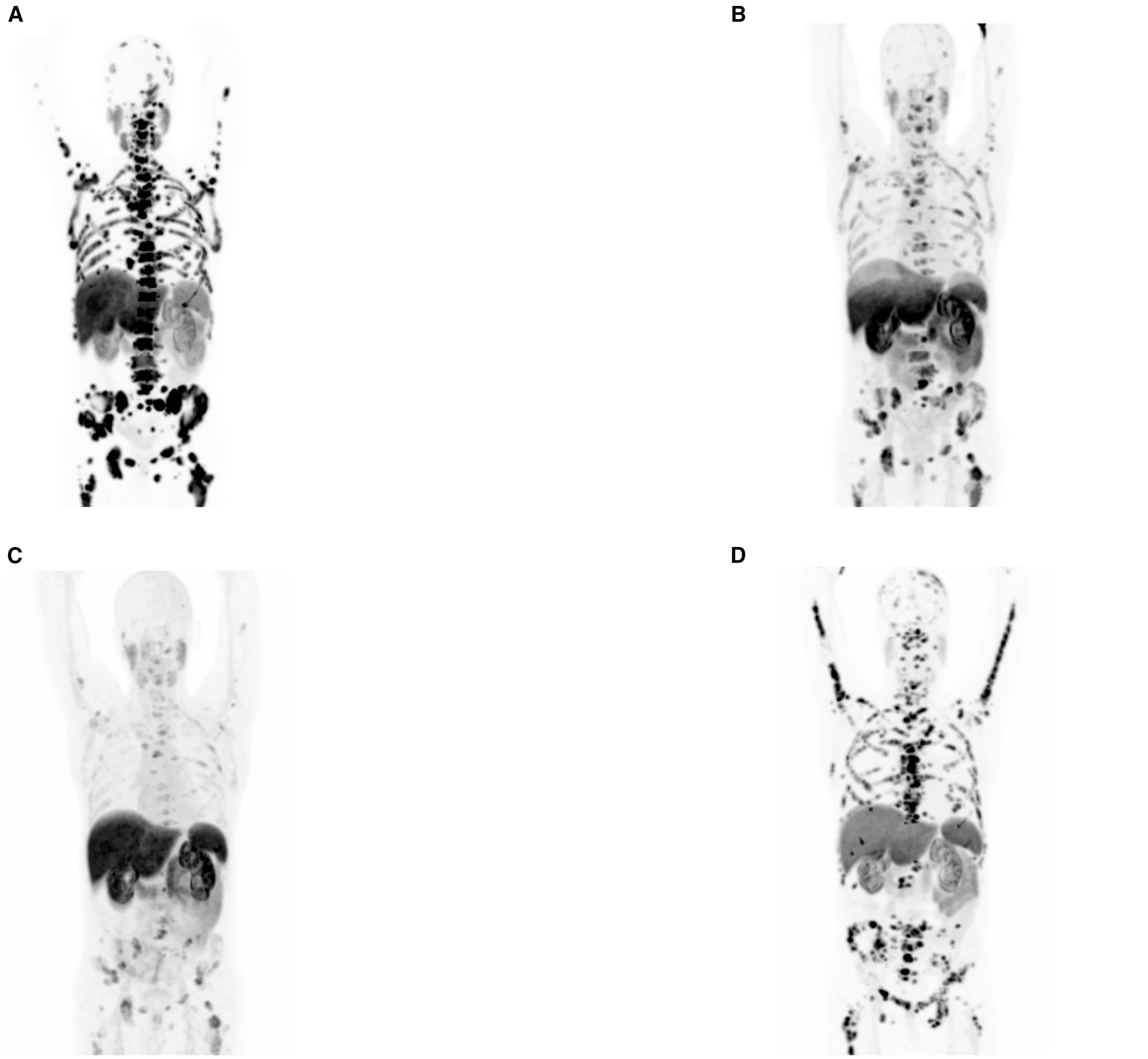

Figure 6. [^{68}Ga]Ga-PSMA-PET scans of a Responder patient (ALM60C8)

(A) A scan showing solid tumor sites before the combined treatment with [^{225}Ac]Ac-PSMA-617-[^{177}Lu]Lu-PSMA-617 (cycle 0). (B) A scan showing solid tumor sites after the 3rd treatment cycle. (C) A follow-up scan ∼3 months after the last recorded treatment cycle. (D) A scan showing the resurgence of the tumor 2 years following the last recorded treatment cycle. The relapsed patient showed resistance to continued [^{225}Ac]Ac-PSMA-617-[^{177}Lu]Lu-PSMA-617 treatment even though the metastatic sites are well visible on the [^{68}Ga]Ga-PSMA-PET scan.

in Clinical Practice," of the revised Declaration of Helsinki. The administered activities ranged from 2 to 8 MBq for [^{225}Ac]Ac-PSMA-617 and 2 to 8.5 GBq for [^{177}Lu]Lu-PSMA-617. In total, 46 heavily pre-treated patients with age ranging from 57 to 88 years (74.3 years average and 75.5 years median) were enrolled for the study. All patients had a high metastatic burden despite receiving multiple lines of prior systemic therapy, including at least one novel androgen-receptor-targeted therapy (e.g., abiraterone or enzalutamide) and/or prior chemotherapy (e.g., docetaxel), and had shown either disease progression or discontinued treatment due to adverse effects. Given the heavily pre-treated nature of the cohort, baseline EpCAM expression reflects therapy-adapted disease rather than early-stage mCRPC biology. Enrollment in the study is contingent upon obtaining written informed consent from patients under ethical approval given by the ethical committee of University Hospital Heidelberg (S-882/2020).

Cell culture

Cell lines were cultured according to standard mammalian tissue culture protocol and sterile technique at 37°C in 5% CO_2. C4-2

and PC-3 cells were cultured in RPMI-1640 media, supplemented with 10% FCS and 1% stable L-glutamine. Routine cell culture was performed twice a week using a room-tempered PBS (pH 7.4) for wash and 0.05% trypsin for cell detachment.

Blood collection

Both patient and healthy control blood was collected in order to isolate CTCs or to optimize the isolating procedure.

Briefly, 2× 9 mL full blood was drawn from patients if health conditions allowed, into 10 mL Vacutainer tubes (BD Biosciences, Franklin Lakes, New Jersey, USA). The collected blood samples were placed on 4°C for 2–4 h until collection. Collected samples were transferred to ice until further buffy coat isolation. In the case of healthy blood used for process optimization, the samples were spiked with 1^2–10^6 C4-2 cells before buffy coat isolation.

Buffy coat isolation and freezing

In order to isolate and preserve CTCs for fluorescent-activated cell sorting, the buffy coat layers of full blood samples were isolated by taking the following steps:

Samples were centrifuged at 800×*g* for 10 min on 4°C, without brake. Following centrifugation, both the plasma and the buffy coat were carefully transferred into 15 mL tubes. Tubes containing plasma were stored on ice until the end of the procedure, then transferred into −80°C for storage.

Tubes containing the buffy coat were supplied with 5× sample volume TheraPEAK ACK lysis buffer (Lonza, Basel, Switzerland, cat # BP10-548E), then gently mixed and incubated for 5 min at room temperature. Following incubation, the samples were centrifuged at 200×*g* for 5 min, then the lysis was repeated once more.

Following complete lysis, the samples were centrifuged at 200×*g* for 5 min and washed with 10 mL of PBS.

After washing, the samples were re-suspended in 500 μL RPMI-1640 media supplemented with 10% FCS. The samples were supplemented with 2.5 nM $MgCl_2$ (Sigma-Aldrich, St. Louis, USA, CAS # 7786-30-3) and 120 IU (32.4 μM) DNAse I (Roche, Basel, Switzerland, cat # 11284932001) and incubated on ice for 30 min. Following incubation, the samples were mixed with an equivalent volume of FCS supplemented with 20% V/V DMSO (Sigma-Aldrich, St. Louis, USA, cat #D2650-100ML) by dropwise mixing.

The mixes were transferred into Mr. Frosty (Nalgene Nunc, Thermo Fisher Scientific, Rochester, USA) freezing containers and placed on −80°C. For storage longer than 3 months, the samples were moved from −80°C to liquid nitrogen.

All samples underwent identical cryopreservation and thawing procedures, minimizing systematic bias between response groups.

Buffy coat processing and CTC staining

In order to set up buffy coat thawing and CTC sorting conditions, C4-2-spiked healthy blood samples were used. Based on these results, further optimization was carried out using real patient samples, which resulted in the finalized procedure consisting of the following main steps.

Buffy coat thawing and surface-marker staining

Frozen buffy coat samples from −80°C or liquid nitrogen were thawed rapidly in a 37°C water bath. To avoid batch effect, Responder and Nonresponder samples were randomly paired, and a two-step isolation process was carried out on the paired buffy coat samples after thawing.

Samples were transferred into 30 mL warm RPMI-1640 media supplemented with 10% FCS, 1 μM Tirofiban Hydrochloride (Sigma-Aldrich, St. Louis, USA, cat # 150915-40-5), 2.5 mM $MgCl_2$, and 600 IU (2.7 μM) DNAse I.

The samples were incubated at room temperature for 30 min to reduce cell aggregation by removing free DNA and inhibiting glycoprotein IIb/IIIa.

Following the incubation, the samples were centrifuged at 200×*g* for 5 min on room temperature, then re-suspended in 200 μL anti-CD45 antibody mix containing 2.5 ng/μL anti-CD45-biotin (eBioscience, San Diego, USA, HI30 cat # 13-0459-82), 2.5 nM $MgCl_2$, and 2 U (27 nM) DNAse I. The samples were placed on ice and incubated for 45 min.

The samples were washed with 1 mL PBS supplemented with 2% FCS (PBS-FCS), then centrifuged on 200×*g* for 5 min.

Following centrifugation, the supernatant was carefully discarded, and the pellet was re-suspended in 240 μL PBS-FCS supplemented with 2.5 nM $MgCl_2$, 2.4 U (27 nM) DNAse I, and 30 μL anti-biotin MicroBeads (Miltenyi, Bergisch Gladbach, Germany, cat # 130-090-485).

The re-suspended samples were incubated on ice for 20 min, then washed with 1 mL PBS-FCS.

Leukocyte depletion, apoptotic staining, and live-dead staining

As the thawed buffy coat is mainly consisting of leukocytes while the CTCs represented an extremely low fraction, we carried out a leukocyte depletion step before cell sorting using a combination of anti-CD45-biotin antibody and magnetic streptavidin beads.

The samples were centrifuged at 200×*g* for 5 min, re-suspended in 500 μL PBS-FCS, and transferred onto LS magnetic separator columns (Miltenyi, Bergisch Gladbach, Germany, cat # 130-042-401) where the CD45-depleted fraction was collected as flow-through.

Following separation, the columns were washed 4× with 2 mL PBS-FCS, and the resulting flow-through was pooled with the CD45-depleted fraction.

Following washing, the columns were removed from the magnetic stand, flushed out with 500 μL PBS-FCS to collect the $CD45^+$ cell containing fraction for further use. This step resulted in a significant reduction in overall cell numbers and allowed us to reduce cell-sort times and preserve CTC viability during the last step of the isolation process.

Next, the CD45-depleted samples were centrifuged at 200×*g* for 5 min, then the supernatant was carefully discarded, and the remaining cells were re-suspended in 160 μL staining-mix containing 1.25 ng/μL Brilliant Violet 421 Streptavidin (BioLegend, San Diego, USA, cat # 405226), Alexa Fluor 488 Anti-EpCAM antibody [323/A3] (Abcam, Cambridge, UK cat # ab253268) in 1:50× dilution, and anti-PSMA antibody REA408—PE-Vio 770 (Miltenyi, Bergisch Gladbach, Germany, cat # 130-131-264) in 1:160× dilution.

The samples were incubated on ice for 60 min, then washed with 1 mL PBS-FCS, centrifuged at 200×*g* for 5 min and re-suspended in 300 μL PBS-FCS, supplemented with 120 nM DAPI (Sigma-Aldrich, St. Louis, USA, cat #D9542-10MG) and 166 nM Incucyte Caspase-3/7 Red Dye (Sartorius, Göttingen, Germany, cat # 4704).

The samples were kept on ice for 15–30 min, which was followed by single-cell sorting on a cell sorter.

Flow cytometry: Single-cell sorting

Stained cell suspensions were sorted using a BD FACSAria Fusion Flow Cytometer using either a 100 μm or 130 μm nozzle (no impact on post-sort quality) with standard BD FACSFlow sheath fluid. The instrument is equipped with five lasers (355 nm UV, 405 nm violet, 488 nm blue, 561 nm yellow-green, and 633 nm red), and further details regarding the optical configuration can be found in Table S5.

Single cells were index-sorted directly into 4.4 μL lysis buffer (0.4% Triton X-100, 2 U/μL RNasin Plus, 1 μM Oligo-dT30VN, 2 μM dNTP) in 96-well plates to enable downstream correlation of EpCAM and PSMA fluorescence intensity with scRNA-seq data. To ensure accuracy and consistency in gating, three types of control samples were routinely used at the beginning of each sorting experiment (Figures S3B–S3D). These included (1) a negative control without antibody staining, (2) a positive control consisting of C4-2 PCa cells processed and stained identically to patient samples, and (3) a $CD45^+$ leukocyte population isolated during the CD45 depletion step for circulating tumor cell (CTC) enrichment, also stained with the same antibody cocktail. The consistent use of all three control samples ensured reliable and reproducible gating across all experiments. The employed gating hierarchy is illustrated in the Supplemental Material (Figure S2F). Following sorting, the plates were stored at −80°C.

Sequencing library preparation

Cells sorted into lysis buffer were processed using a modified version of the SmartSeq2 protocol, based on the works by Picelli et al., 2014[22] and Hennig et al., 2018.[54] As RNA degradation during sample processing cannot be excluded, the Smart-Seq2 method was selected for its robustness in low-input single-cell applications.

Reverse transcription

Samples were thawed on ice after being removed from −80°C and placed into a thermocycler, where they were heat-lysed by incubating at 72°C for 3 min.

The lysed samples were then cooled on ice for at least 2 min before being supplied with 6.35 μL of reverse transcription (RT) master mix per sample. For a single sample, the RT master mix contained 2 μL SSRT IV 5× buffer (Invitrogen/Thermo Fisher Scientific, Waltham, USA, cat # 18091050), 0.5 μL 100 mM DTT, 2 μL 5M betaine, 0.1 μL 1M $MgCl_2$, 0.25 μL RNAse inhibitor (40 U/μL, Clontech Takara, Kusatsu, Japan, cat # 2313A), 0.25 μL SSRT IV (Invitrogen/Thermo, Waltham, USA, cat # 18091050), and 0.1 μL 100 μM Template Switching Oligo (TSO; sequence in Table S7).

The mixture was then pulse-vortexed and placed in a thermocycler for RT, using the following program: 52°C for 15 min, 80°C for 10 min, stored at 10°C.

Pre-amplification

The samples were cooled on ice and supplied with 15 μL of amplification master mix per sample. The amplification master mix contained 12.5 μL of KAPA HiFi Hot start ReadyMix (Roche, Basel, Switzerland, cat # KK2601) and 2.5 μL of H_2O.

The mixture was then pulse-vortexed and placed into a thermocycler for pre-amplification, using the following program: 98°C for 3 min; 23 cycles of 98°C for 20 s, 67°C for 15 s, and 72°C for 6 min; 72°C for 5 min; stored at 10°C.

cDNA purification I

The samples were filled up with H_2O to a final volume of 50 μL, then each sample was supplied with a 0.6× sample volume of solid-phase reversible immobilization (SPRI) beads (Beckman Coulter, Brea, USA). The samples were mixed by pulse-vortexing and were let to incubate for 15 min at room temperature.

Following the incubation, the samples were placed on a magnet, and after bead sedimentation the supernatant was carefully removed. The samples were immediately eluted by adding 13 μL of fresh H_2O to the beads, pulse vortexing them, and incubating for 3 min at room temperature.

Following incubation, the samples were placed back on a magnet, and 12 μL of the supernatant was carefully transferred into new plates.

Quality control I

The eluted samples were analyzed using the Agilent Fragment Analyzer Systems in combination with the HS NGS Fragment kit (Agilent, Santa Clara, USA, cat #DNF-474) according to the manufacturer's recommendations. Samples were selected for further processing based on their cDNA content and fragment analysis profile between the 200 and 1,500 bp range.

Primer annealing

Lyophilized Tn5ME-A, Tn5ME-B, and Tn5RMErev primers (for sequences see Table S7) were re-suspended in annealing buffer (50 mM NaCl, 40 mM Tris-HCl pH 8.0) to a concentration of 100 μM.

Tn5ME-A or Tn5ME-B was mixed with Tn5Merev in a 1:1 ratio in a 50 μM final concentration and annealed in a thermocycler, using the following program: 95°C for 5 min, cool down to 65°C (ramp-down rate: 0.1°C/s), keep at 65°C for 5 min, and finally slowly cool down to 4°C (ramp-down rate: 0.1°C/s).

The annealed oligonucleotides were diluted to 35 μM with H_2O and store at −20°C until use.

Transposase loading

The Tn5 enzyme (0.9 mg/mL, $Tn5_{(R27S),E54K,L372P}$, EMBL-Protein Expression and Purification Core Facility, Heidelberg, Germany) was diluted in H_2O in a 1:1 ratio, in 10 μL final volume, then mixed with 0.7 μL annealed Tn5ME-A/Tn5Merev and Tn5ME-B/Tn5Merev oligoes, pulse-vortexed, and placed in a shaker at 23°C, 350 rpm for 45 min. Following pre-loading, the Tn5 mix was diluted in 1:450 ratio in H_2O and was kept on ice until use.

As cDNA concentration after pre-amplification was generally low (median 0.4 ng/μL), 4 μL of each sample was transferred into a new 96-well plate and was concentrated to ∼1–1.5 μL using a SpeedVac (Eppendorf, Hamburg, Germany) vacuum concentrator.

Following concentration, each sample was supplied with 1.25 μL Tn5 mix (1:450 dilution), 1.25 μL 4× tagmentation mix (40 mM Tris-HCl pH 7.5, 40 mM $MgCl_2$), and 1.25 μL 100% DMF (Sigma-Aldrich, St. Louis, USA), mixed well by pipetting, and incubated on 55°C for 3 min in a thermocycler.

Following incubation, the samples were cooled on ice for a short time, then they were supplied with 1.25 μL of 0.2% SDS, mixed well, and incubated on room temperature for 5 min to stop the tagmentation. Following incubation, the samples were stored on ice until further use.

PCR amplification

Samples were supplied with 6.75 μL Kappa HiFi Hot start ReadyMix (cat # KK2601), 0.75 μL 100% DMSO (Sigma-Aldrich, St. Louis, USA), and 2.5 μL i5/i7adapter index primer mix (Table S4).

The samples were mixed and amplified in a thermocycler using the following program: 72°C for 3 min; 18 cycles of 95°C for 30 s, 98°C for 20 s, 58°C for 15 s, and 72°C for 30 s; 72°C for 3 min, stored at 10°C.

cDNA purification II

Following final PCR amplification, the samples were filled up with H_2O to a final volume of 50 μL, then each sample was supplied with a 0.7× sample volume of SPRI beads.

The samples were mixed by pulse vortexing and were let to incubate for 10 min on room temperature, then the samples were placed on a magnet, and after bead sedimentation the supernatant was carefully removed. The samples were washed 2× with 200 μL 80% EtOH (30 s each) and were dried for 2 min at room temperature.

After drying, the samples were eluted by adding 14 μL of fresh H_2O to the beads, pulse-vortexing them, and incubating for 3 min at room temperature. Following incubation, the samples were placed back on a magnet, and 13 μL of the supernatant was carefully transferred into new plates.

Quality control II and concentration adjustment

The transferred samples were analyzed using the Agilent Fragment Analyzer Systems in combination with the HS NGS Fragment kit (Agilent, Santa Clara, USA, cat #DNF-474) according to the manufacturer's recommendations.

The molarity of each sample was calculated based on their fragment analysis profile in the 200–1,500 bp range, using the ProSize data analysis software (Agilent, Santa Clara, USA, version 4.0.2.7). The resulting .csv files were further processed in R v.4.2.3, where a median molar concentration of 6.95 nM was calculated (see the [CTC_scRNA-seq_fragment_cleanup_and_analysis.R] analysis script on GitHub [https://github.com/UBE2C/psma-epcam-scrna-flow]).

Samples were divided into above median and below median groups based on the calculated median molar concentration. Samples above median molar concentration were diluted to the median concentration in a 5 μL final volume in a new 96-well plate. Following dilution, the samples were pooled by transferring approximately 6.95 fmol from each sample into a 1.5 mL DNA LoBind Tube (Eppendorf, Hamburg, Germany). Samples below 1 nM were discarded.

The resulting sample pool was concentrated using SPRI beads as described at the end of sample tagmentation, except that the bead volume was 1× of the sample volume, and the final elution volume was 25 μL.

Sample multiplexing and final quality control

Below median and above median sample pools were mixed in an equimolar concentration and was assessed using a Bioanalyzer 2100 (Agilent, Santa Clara, USA). The measurement showed a relatively high number of fragments between the lengths of 150 and 300 bp. The final sample pool was once again cleaned by SPRI beads,

as described at the end of sample tagmentation, except that the bead volume was 0.65× of the sample volume. Following cleanup, the final sample multiplex was assessed one last time by a Bioanalyzer 2100 and was used for sequencing.

RNA sequencing

The prepared dual barcoded libraries were sequenced on an Illumina NextSeq2000 sequencer using the P3 flow cell in a single-end 50 bp configuration.

Flow cytometry data analysis

Index-sort FCS files (C4-2 control, patient) were loaded into R v.4.2.3 with flowCore v.2.16.

Data loading and quality control

The obtained .fcs files were loaded into two separate R lists (C4-2 control list and sample list) using the flowCore::read.FCS function (default arguments). The median of the MFI) values of each recorded channel were calculated for the C4-2 control samples. As part of the quality control (QC) process, any duplicated entries were removed.

To ensure the recorded control samples are fit to be used for normalization, the control samples were evaluated by calculating and plotting the grand median, median absolute deviation (MAD), and median absolute deviation of the medians (MADM) for each control sample. Comparison between the grand median values was done using a Mann-Whitney U test (Figure S4B).

Next, any sample name errors for sorted CTCs were fixed, and any sort control samples were filtered out to match the sorted samples. The cleaned and fixed patient sample sort data were merged into a single data frame for MFI normalization.

Data normalization, MFI artifact correction, and filtering

MFI values of all fluorescent channels were normalized, to account for the sort-to-sort variation and to facilitate a more precise sample comparison. MFI values of each sorted patient sample were divided by the session-matched control median MFI. Samples from patients with unknown treatment outcomes were excluded, and negative EpCAM and PSMA MFI values—artefacts of baseline subtraction—were imputed. To ensure that imputed values remained close to the detection baseline while avoiding value duplication, a channel-specific drawing set was constructed from the 10 lowest positive MFI values. Two values were randomly drawn from this set, and their mean was used as the imputed value.

Following imputation, the data were filtered on both EpCAM and PSMA channels to remove outliers with exceptionally high and low MFI values (Figures S4C and S4D).

Marker dynamics, population distribution, and statistical analysis

Following data preparation, parameters such as marker expression, marker distribution, and marker dynamics over treatment cycles were analyzed using the base stats R package. Statistics involved median, MAD, Mann-Whitney U test, Shapiro-Wilk test of normality, and χ^2 goodness-of-fit test, respectively.

The obtained data were visualized using ggplot2 v.3.5.1, ggsci v.3.2.0, and ggpubr v.0.6.0 R packages and Affinity Designer 2. For the detailed analysis, see the [flow Cytometry_analysis_script_V1.0.0.R] analysis script on GitHub [https://github.com/UBE2C/psma-epcam-scrna-flow].

scRNA-seq: Read alignment and filtering

Following sequencing, raw reads were demultiplexed, adapter- and quality-trimmed, and aligned to the GRCh38 reference with STAR v.2.7.10b (–quantMode). The resulting ReadsPerGene.out.tab files were parsed in R v.4.3.1–4.4.1 to extract ENSEMBL ID-based gene identifiers and unstranded read counts, which were aggregated into a single gene-by-cell matrix and combined with sample metadata into a Seurat v.4.3.0.1 object for downstream analysis.

QC was performed to remove potential duplets and low-quality cells from the dataset. Cells with <4,000 total counts, <1,000 or >7,800 detected genes, or >75% mitochondrial reads were removed (Figures S5B–S5E). A lenient mitochondrial cutoff was chosen, given our dual live-dead/apoptotic sorting, potential radiation-induced mitochondrial upregulation, and the higher MT read fraction typical of Smart-Seq2 (for a detailed explanation see supplemental methods).

scRNA-seq: Data normalization, clustering, and composition analysis

Gene counts were normalized and variance-stabilized with Seurat's SCTransform (glmGamPoi method, glmGamPoi package v.1.14.0) before PCA on the top 3,000 highly variable genes. The first 20 PCs—determined by an elbow plot—were used to compute a UMAP embedding (dims 1:20) for visualization and downstream clustering. Cell neighbors were identified with FindNeighbors (dims = 1:20), and clusters were defined via FindClusters (resolution = 0.6). Cluster composition was then compared across treatment variables (detailed/overall response status, treatment type, cycle, cell-cycle phase, and surface marker status) by χ^2 tests of independence ($\alpha = 0.05$).

scRNA-seq: Differential gene expression and gene set enrichment analysis

Differential expression analysis between clusters was performed with Seurat v.5.0.2's FindMarkers (default Wilcoxon test). Resulting genes were annotated with gene symbols and Entrez IDs, while entries lacking symbols were removed. Genes were ranked by $\log_2$ FC × ($-\log_{10}$ adjusted p value) and plotted as a volcano plot (ggplot2; see Figure 5A).

GSEA was conducted with irGSEA v3.3.2 using standard settings and the following msigbdr v.7.5.1 collections: C2 (BIOCARTA, KEGG, REACTOME, WIKIPATHWAYS), C3 (TFT:GTRD), C4 (CGN,

CM), C5 (GO:BP, GO:MF, GO:CC), C6, and H. Enrichment results were integrated and visualized via irGSEA.integrate and irGSEA.heatmap. Hub genes within significant pathways were identified with irGSEA.hub and filtered for entries with a rank-correlation score of at least 0.2, a Spearman's *p* value of <0.05 in at least two methods, and with a significant DEG status. Data visualization was done using ggplot2 v.3.5.1, ggsci v.3.2.0, and ggpubr v.0.6.0 R packages and Affinity Designer 2. For the detailed analysis, see the [CTC_scRNA-seq_data_analysis_script_V1.0.0.R] analysis script on GitHub [https://github.com/UBE2C/psma-epcam-scrna-flow].

DATA AND CODE AVAILABILITY

All data supporting the findings of this study are available from the corresponding author on reasonable request.

ACKNOWLEDGMENTS

Co-funding of the research group Molecular Biology of Systemic Radiotherapy was made available by industrial collaboration with Bayer AG, Berlin, Germany. We would like to thank the EMBL Flow Cytometry Core facility and the EBML Gene Core for all the help and support they provided for the project. This research received no external funding.

AUTHOR CONTRIBUTIONS

Conceptualization, G.B., M.R., C.K., and M.B.-S.; patient recruitment and blood collection, C.K.; methodology, G.B., U.B.-W., B.R., and M.B.-S.; investigation, G.B., U.B.-W., B.R., and M.B.-S.; data analysis and interpretation, G.B., J.L., B.R., and M.B.-S.; visualization, G.B. and B.R.; writing—original draft, G.B. and M.B.-S.; writing—review & editing, G.B., B.R., J.L., M.R. C.K., F.B., A.M., V.B., and M.B.-S.; funding acquisition, M.B.-S.; supervision, G.B. and M.B.-S.

DECLARATION OF INTERESTS

Patent application on PSMA-617 has been filed by German Cancer Research Center (DKFZ) and University Clinic (UKHD) in Heidelberg, Germany. U.B.W., C.K., and M.B.S. are co-inventors of this patent. No other potential conflicts of interest relevant to this article were reported.

SUPPLEMENTAL INFORMATION

Supplemental information can be found online at https://doi.org/10.1016/j.omton.2026.201143.

REFERENCES

1. Morgenstern, A., Apostolidis, C., Kratochwil, C., Sathekge, M., Krolicki, L., and Bruchertseifer, F. (2018). An Overview of Targeted Alpha Therapy with (225)Actinium and (213)Bismuth. Curr. Radiopharm. *11*, 200–208. https://doi.org/10.2174/1874471011666180502104524.

2. Miederer, M., Benesova-Schafer, M., Mamat, C., Kastner, D., Pretze, M., Michler, E., Brogsitter, C., Kotzerke, J., Kopka, K., Scheinberg, D.A., and McDevitt, M.R. (2024). Alpha-Emitting Radionuclides: Current Status and Future Perspectives. Pharmaceuticals *17*, 76. https://doi.org/10.3390/ph17010076.

3. Sathekge, M.M., Bruchertseifer, F., Vorster, M., Morgenstern, A., and Lawal, I.O. (2021). Global experience with PSMA-based alpha therapy in prostate cancer. Eur. J. Nucl. Med. Mol. Imaging *49*, 30–46. https://doi.org/10.1007/s00259-021-05434-9.

4. Ballal, S., Yadav, M.P., Sahoo, R.K., Tripathi, M., Dwivedi, S.N., and Bal, C. (2021). (225) Ac-PSMA-617-targeted alpha therapy for the treatment of metastatic castration-resistant prostate cancer: A systematic review and meta-analysis. Prostate *81*, 580–591. https://doi.org/10.1002/pros.24137.

5. Kratochwil, C., Giesel, F.L., Heussel, C.P., Kazdal, D., Endris, V., Nientiedt, C., Bruchertseifer, F., Kippenberger, M., Rathke, H., Leichsenring, J., et al. (2020). Patients Resistant Against PSMA-Targeting alpha-Radiation Therapy Often Harbor Mutations in DNA Damage-Repair-Associated Genes. J. Nucl. Med. *61*, 683–688. https://doi.org/10.2967/jnumed.119.234559.

6. Noor, J., Chaudhry, A., Noor, R., and Batool, S. (2023). Advancements and Applications of Liquid Biopsies in Oncology: A Narrative Review. Cureus *15*, e42731. https://doi.org/10.7759/cureus.42731.

7. Allen, T.A. (2024). The Role of Circulating Tumor Cells as a Liquid Biopsy for Cancer: Advances, Biology, Technical Challenges, and Clinical Relevance. Cancers (Basel) *16*, 1377. https://doi.org/10.3390/cancers16071377.

8. Schwartz, L.H., Litière, S., de Vries, E., Ford, R., Gwyther, S., Mandrekar, S., Shankar, L., Bogaerts, J., Chen, A., Dancey, J., et al. (2016). RECIST 1.1-Update and clarification: From the RECIST committee. Eur. J. Cancer *62*, 132–137. https://doi.org/10.1016/j.ejca.2016.03.081.

9. Hartrampf, P.E., Hüttmann, T., Seitz, A.K., Kübler, H., Serfling, S.E., Higuchi, T., Schlötelburg, W., Michalski, K., Gafita, A., Rowe, S.P., et al. (2024). Prognostic Performance of RECIP 1.0 Based on [(18)F]PSMA-1007 PET in Prostate Cancer Patients Treated with [(177)Lu]Lu-PSMA I&T. J. Nucl. Med. *65*, 560–565. https://doi.org/10.2967/jnumed.123.266702.

10. Nejlund, S., Smith, J., Kraan, J., Stender, H., Van, M.N., Langkjer, S.T., Nielsen, M.T., Sölétormos, G., and Hillig, T. (2016). Cryopreservation of Circulating Tumor Cells for Enumeration and Characterization. Biopreserv. Biobank. *14*, 330–337. https://doi.org/10.1089/bio.2015.0074.

11. Allard, W.J., Matera, J., Miller, M.C., Repollet, M., Connelly, M.C., Rao, C., Tibbe, A.G.J., Uhr, J.W., and Terstappen, L.W.M.M. (2004). Tumor cells circulate in the peripheral blood of all major carcinomas but not in healthy subjects or patients with nonmalignant diseases. Clin. Cancer Res. *10*, 6897–6904. https://doi.org/10.1158/1078-0432.CCR-04-0378.

12. Eslami-S, Z., Cortes-Hernandez, L.E., and Alix-Panabieres, C. (2020). Epithelial Cell Adhesion Molecule: An Anchor to Isolate Clinically Relevant Circulating Tumor Cells. Cells *9*, 1836. https://doi.org/10.3390/cells9081836.

13. Po, J.W., Roohullah, A., Lynch, D., DeFazio, A., Harrison, M., Harnett, P.R., Kennedy, C., de Souza, P., and Becker, T.M. (2018). Improved ovarian cancer EMT-CTC isolation by immunomagnetic targeting of epithelial EpCAM and mesenchymal N-cadherin. J. Circ. Biomark. *7*. 1849454418782617. https://doi.org/10.1177/1849454418782617.

14. Silver, D.A., Pellicer, I., Fair, W.R., Heston, W.D., and Cordon-Cardo, C. (1997). Prostate-specific membrane antigen expression in normal and malignant human tissues. Clin. Cancer Res. *3*, 81–85.

15. Bravaccini, S., Puccetti, M., Bocchini, M., Ravaioli, S., Celli, M., Scarpi, E., De Giorgi, U., Tumedei, M.M., Raulli, G., Cardinale, L., and Paganelli, G. (2018). PSMA expression: a potential ally for the pathologist in prostate cancer diagnosis. Sci. Rep. *8*, 4254. https://doi.org/10.1038/s41598-018-22594-1.

16. Benesova, M., Schafer, M., Bauder-Wust, U., Afshar-Oromieh, A., Kratochwil, C., Mier, W., Haberkorn, U., Kopka, K., and Eder, M. (2015). Preclinical Evaluation of a Tailor-Made DOTA-Conjugated PSMA Inhibitor with Optimized Linker Moiety for Imaging and Endoradiotherapy of Prostate Cancer. J. Nucl. Med. *56*, 914–920. https://doi.org/10.2967/jnumed.114.147413.

17. Jang, A., Kendi, A.T., and Sartor, O. (2023). Status of PSMA-targeted radioligand therapy in prostate cancer: current data and future trials. Ther. Adv. Med. Oncol. *15*. 17588359231157632. https://doi.org/10.1177/17588359231157632.

18. Liu, H., Moy, P., Kim, S., Xia, Y., Rajasekaran, A., Navarro, V., Knudsen, B., and Bander, N.H. (1997). Monoclonal antibodies to the extracellular domain of prostate-specific membrane antigen also react with tumor vascular endothelium. Cancer Res. *57*, 3629–3634.

19. Stylianou, N., Lehman, M.L., Wang, C., Fard, A.T., Rockstroh, A., Fazli, L., Jovanovic, L., Ward, M., Sadowski, M.C., Kashyap, A.S., et al. (2019). A molecular portrait of epithelial-mesenchymal plasticity in prostate cancer associated with clinical outcome. Oncogene *38*, 913–934. https://doi.org/10.1038/s41388-018-0488-5.

20. Sayar, E., Patel, R.A., Coleman, I.M., Roudier, M.P., Zhang, A., Mustafi, P., Low, J.Y., Hanratty, B., Ang, L.S., Bhatia, V., et al. (2023). Reversible epigenetic alterations mediate PSMA expression heterogeneity in advanced metastatic prostate cancer. JCI Insight *8*, e162907. https://doi.org/10.1172/jci.insight.162907.

21. Chai, S., Ruiz-Velasco, C., Naghdloo, A., Pore, M., Singh, M., Matsumoto, N., Kolatkar, A., Xu, L., Shishido, S., Aparicio, A., et al. (2022). Identification of epithelial and mesenchymal circulating tumor cells in clonal lineage of an aggressive

prostate cancer case. npj Precis. Oncol. *6*, 41. https://doi.org/10.1038/s41698-022-00289-1.

22. Picelli, S., Faridani, O.R., Björklund, A.K., Winberg, G., Sagasser, S., and Sandberg, R. (2014). Full-length RNA-seq from single cells using Smart-seq2. Nat. Protoc. *9*, 171–181. https://doi.org/10.1038/nprot.2014.006.

23. Fan, C., Chen, F., Chen, Y., Huang, L., Wang, M., Liu, Y., Wang, Y., Guo, H., Zheng, N., Liu, Y., et al. (2024). irGSEA: the integration of single-cell rank-based gene set enrichment analysis. Brief. Bioinform. *25*, bbae243. https://doi.org/10.1093/bib/bbae243.

24. James, N.D., Tannock, I., N'Dow, J., Feng, F., Gillessen, S., Ali, S.A., Trujillo, B., Al-Lazikani, B., Attard, G., Bray, F., et al. (2024). The Lancet Commission on prostate cancer: planning for the surge in cases. Lancet *403*, 1683–1722. https://doi.org/10.1016/S0140-6736(24)00651-2.

25. Ballal, S., Yadav, M.P., Satapathy, S., Raju, S., Tripathi, M., Damle, N.A., Sahoo, R.K., and Bal, C. (2023). Long-term survival outcomes of salvage [(225)Ac]Ac-PSMA-617 targeted alpha therapy in patients with PSMA-expressing end-stage metastatic castration-resistant prostate cancer: a real-world study. Eur. J. Nucl. Med. Mol. Imaging *50*, 3777–3789. https://doi.org/10.1007/s00259-023-06340-y.

26. Masud, T., Soong, C., Xu, H., Biele, J., Bjornson, S., McKinney, S., and Aparicio, S. (2021). Ubiquitin-mediated DNA damage response is synthetic lethal with G-quadruplex stabilizer CX-5461. Sci. Rep. *11*, 9812. https://doi.org/10.1038/s41598-021-88988-w.

27. Ulrich, H.D., and Jentsch, S. (2000). Two RING finger proteins mediate cooperation between ubiquitin-conjugating enzymes in DNA repair. EMBO J. *19*, 3388–3397. https://doi.org/10.1093/emboj/19.13.3388.

28. Huen, M.S.Y., Huang, J., Yuan, J., Yamamoto, M., Akira, S., Ashley, C., Xiao, W., and Chen, J. (2008). Noncanonical E2 variant-independent function of UBC13 in promoting checkpoint protein assembly. Mol. Cell Biol. *28*, 6104–6112. https://doi.org/10.1128/MCB.00987-08.

29. Zhao, G.Y., Sonoda, E., Barber, L.J., Oka, H., Murakawa, Y., Yamada, K., Ikura, T., Wang, X., Kobayashi, M., Yamamoto, K., et al. (2007). A critical role for the ubiquitin-conjugating enzyme Ubc13 in initiating homologous recombination. Mol. Cell *25*, 663–675. https://doi.org/10.1016/j.molcel.2007.01.029.

30. Liu, S., Hua, Y., Wang, J., Li, L., Yuan, J., Zhang, B., Wang, Z., Ji, J., and Kong, D. (2021). RNA polymerase III is required for the repair of DNA double-strand breaks by homologous recombination. Cell *184*, 1314–1329.e10. https://doi.org/10.1016/j.cell.2021.01.048.

31. Gyrd-Hansen, M., and Meier, P. (2010). IAPs: from caspase inhibitors to modulators of NF-kappaB, inflammation and cancer. Nat. Rev. Cancer *10*, 561–574. https://doi.org/10.1038/nrc2889.

32. Adams, T.E., Epa, V.C., Garrett, T.P., and Ward, C.W. (2000). Structure and function of the type 1 insulin-like growth factor receptor. Cell. Mol. Life Sci. *57*, 1050–1093. https://doi.org/10.1007/PL00000744.

33. Ofer, P., Heidegger, I., Eder, I.E., Schöpf, B., Neuwirt, H., Geley, S., Klocker, H., and Massoner, P. (2015). Both IGF1R and INSR Knockdown Exert Antitumorigenic Effects in Prostate Cancer In Vitro and In Vivo. Mol. Endocrinol. *29*, 1694–1707. https://doi.org/10.1210/me.2015-1073.

34. Evergren, E., Mills, I.G., and Kennedy, G. (2024). Adaptations of membrane trafficking in cancer and tumorigenesis. J. Cell Sci. *137*, jcs260943. https://doi.org/10.1242/jcs.260943.

35. Jin, H., Tang, Y., Yang, L., Peng, X., Li, B., Fan, Q., Wei, S., Yang, S., Li, X., Wu, B., et al. (2021). Rab GTPases: Central Coordinators of Membrane Trafficking in Cancer. Front. Cell Dev. Biol. *9*, 648384. https://doi.org/10.3389/fcell.2021.648384.

36. Goueli, B.S., Powell, M.B., Finger, E.C., and Pfeffer, S.R. (2012). TBC1D16 is a Rab4A GTPase activating protein that regulates receptor recycling and EGF receptor signaling. Proc. Natl. Acad. Sci. USA *109*, 15787–15792. https://doi.org/10.1073/pnas.1204540109.

37. Xu, S., Cao, B., Xuan, G., Xu, S., An, Z., Zhu, C., Li, L., and Tang, C. (2024). Function and regulation of Rab GTPases in cancers. Cell Biol. Toxicol. *40*, 28. https://doi.org/10.1007/s10565-024-09866-5.

38. Perrin, L., Lacas-Gervais, S., Gilleron, J., Ceppo, F., Prodon, F., Benmerah, A., Tanti, J.F., and Cormont, M. (2013). Rab4b controls an early endosome sorting event by interacting with the gamma-subunit of the clathrin adaptor complex 1. J. Cell Sci. *126*, 4950–4962. https://doi.org/10.1242/jcs.130575.

39. Moore, R.H., Millman, E.E., Alpizar-Foster, E., Dai, W., and Knoll, B.J. (2004). Rab11 regulates the recycling and lysosome targeting of beta2-adrenergic receptors. J. Cell Sci. *117*, 3107–3117. https://doi.org/10.1242/jcs.01168.

40. O'Keefe, D.S., Bacich, D.J., Huang, S.S., and Heston, W.D.W. (2018). A Perspective on the Evolving Story of PSMA Biology, PSMA-Based Imaging, and Endoradiotherapeutic Strategies. J. Nucl. Med. *59*, 1007–1013. https://doi.org/10.2967/jnumed.117.203877.

41. Papaioannou, P., Wallace, M.J., Malhotra, N., Mohler, P.J., and El Refaey, M. (2023). Biochemical Structure and Function of TRAPP Complexes in the Cardiac System. JACC. Basic Transl. Sci. *8*, 1599–1612. https://doi.org/10.1016/j.jacbts.2023.03.011.

42. Szul, T., and Sztul, E. (2011). COPII and COPI traffic at the ER-Golgi interface. Physiology *26*, 348–364. https://doi.org/10.1152/physiol.00017.2011.

43. Roosterman, D., Schmidlin, F., and Bunnett, N.W. (2003). Rab5a and rab11a mediate agonist-induced trafficking of protease-activated receptor 2. Am. J. Physiol. Cell Physiol. *284*, C1319–C1329. https://doi.org/10.1152/ajpcell.00540.2002.

44. Johnson, I.R.D., Parkinson-Lawrence, E.J., Keegan, H., Spillane, C.D., Barry-O'Crowley, J., Watson, W.R., Selemidis, S., Butler, L.M., O'Leary, J.J., and Brooks, D.A. (2015). Endosomal gene expression: a new indicator for prostate cancer patient prognosis? Oncotarget *6*, 37919–37929. https://doi.org/10.18632/oncotarget.6114.

45. Agell, L., Hernández, S., Salido, M., de Muga, S., Juanpere, N., Arumí-Uria, M., Menendez, S., Lorenzo, M., Lorente, J.A., Serrano, S., and Lloreta, J. (2011). PI3K signaling pathway is activated by PIK3CA mRNA overexpression and copy gain in prostate tumors, but PIK3CA, BRAF, KRAS and AKT1 mutations are infrequent events. Mod. Pathol. *24*, 443–452. https://doi.org/10.1038/modpathol.2010.208.

46. Herberts, C., Murtha, A.J., Fu, S., Wang, G., Schönlau, E., Xue, H., Lin, D., Gleave, A., Yip, S., Angeles, A., et al. (2020). Activating AKT1 and PIK3CA Mutations in Metastatic Castration-Resistant Prostate Cancer. Eur. Urol. *78*, 834–844. https://doi.org/10.1016/j.eururo.2020.04.058.

47. Glaviano, A., Foo, A.S.C., Lam, H.Y., Yap, K.C.H., Jacot, W., Jones, R.H., Eng, H., Nair, M.G., Makvandi, P., Geoerger, B., et al. (2023). PI3K/AKT/mTOR signaling transduction pathway and targeted therapies in cancer. Mol. Cancer *22*, 138. https://doi.org/10.1186/s12943-023-01827-6.

48. Faes, S., and Dormond, O. (2015). PI3K and AKT: Unfaithful Partners in Cancer. Int. J. Mol. Sci. *16*, 21138–21152. https://doi.org/10.3390/ijms160921138.

49. Mahajan, K., and Mahajan, N.P. (2012). PI3K-independent AKT activation in cancers: a treasure trove for novel therapeutics. J. Cell. Physiol. *227*, 3178–3184. https://doi.org/10.1002/jcp.24065.

50. Vellky, J.E., Kirkpatrick, B.J., Gutgesell, L.C., Morales, M., Brown, R.M., Wu, Y., Maienschein-Cline, M., Notardonato, L.D., Weinfeld, M.S., Nguyen, R.H., et al. (2024). ERBB3 Overexpression is Enriched in Diverse Patient Populations with Castration-sensitive Prostate Cancer and is Associated with a Unique AR Activity Signature. Clin. Cancer Res. *30*, 1530–1543. https://doi.org/10.1158/1078-0432.CCR-23-2161.

51. Ruiz-Saenz, A., Dreyer, C., Campbell, M.R., Steri, V., Gulizia, N., and Moasser, M.M. (2018). HER2 Amplification in Tumors Activates PI3K/Akt Signaling Independent of HER3. Cancer Res. *78*, 3645–3658. https://doi.org/10.1158/0008-5472.CAN-18-0430.

52. Kratochwil, C., Giesel, F.L., Stefanova, M., Benešová, M., Bronzel, M., Afshar-Oromieh, A., Mier, W., Eder, M., Kopka, K., and Haberkorn, U. (2016). PSMA-Targeted Radionuclide Therapy of Metastatic Castration-Resistant Prostate Cancer with 177Lu-Labeled PSMA-617. J. Nucl. Med. *57*, 1170–1176. https://doi.org/10.2967/jnumed.115.171397.

53. Kratochwil, C., Bruchertseifer, F., Giesel, F.L., Weis, M., Verburg, F.A., Mottaghy, F., Kopka, K., Apostolidis, C., Haberkorn, U., and Morgenstern, A. (2016). 225Ac-PSMA-617 for PSMA-Targeted alpha-Radiation Therapy of Metastatic Castration-Resistant Prostate Cancer. J. Nucl. Med. *57*, 1941–1944. https://doi.org/10.2967/jnumed.116.178673.

54. Hennig, B.P., Velten, L., Racke, I., Tu, C.S., Thoms, M., Rybin, V., Besir, H., Remans, K., and Steinmetz, L.M. (2018). Large-Scale Low-Cost NGS Library Preparation Using a Robust Tn5 Purification and Tagmentation Protocol. G3 (Bethesda) *8*, 79–89. https://doi.org/10.1534/g3.117.300257.

OMTON, Volume *34*

Supplemental information

EpCAM–PSMA: Potential predictors of treatment outcomes for PSMA-targeted alpha therapies in metastatic castration-resistant prostate cancer

Gábor Bakos, Ulrike Bauder-Wüst, Jonathan Landry, Mareike Roscher, Beáta Ramasz, Frank Bruchertseifer, Alfred Morgenstern, Clemens Kratochwil, Vladimír Beneš, and Martina Benešová-Schäfer

Supplemental Figures

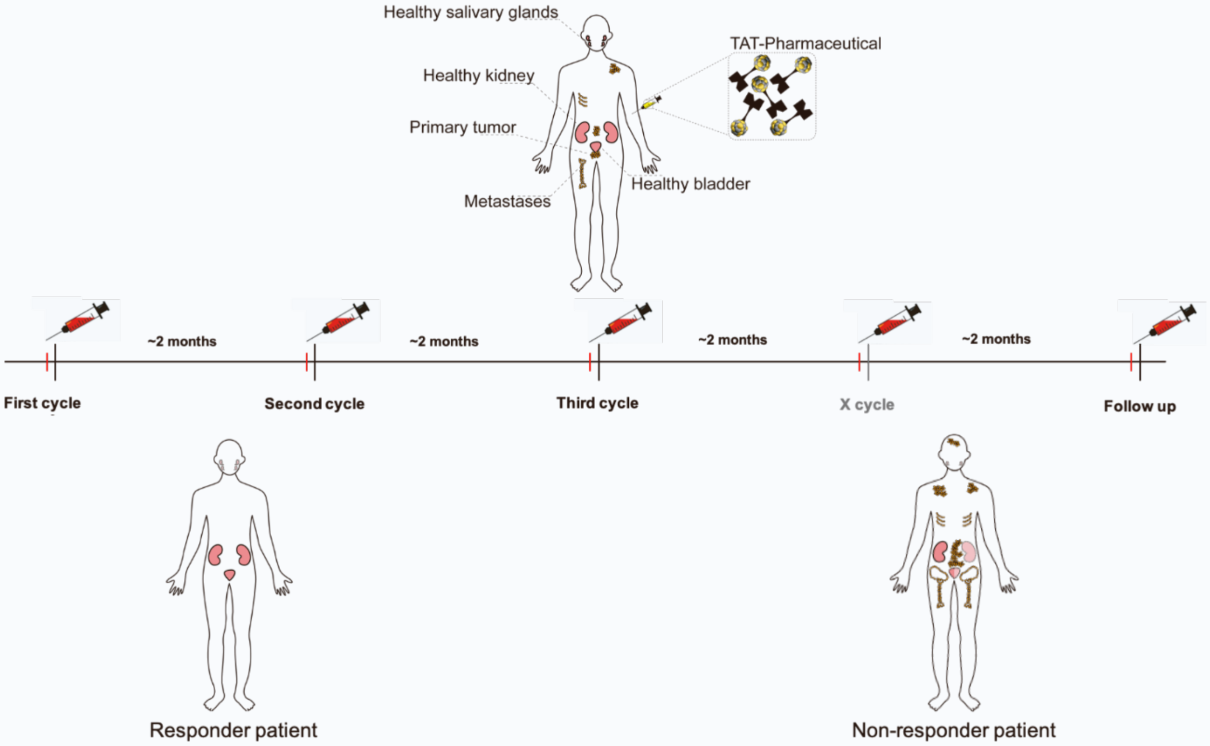

Figure S1. Blood collection layout. Patients with mCRPC who are receiving [^{177}Lu]Lu-PSMA-617 and [^{225}Ac]Ac-PSMA-617 treatment were selected by physicians for blood collection. Blood samples were collected before drug administration for each treatment cycle. The blood samples were prepared for storage the same day as the collection. Later, the patients were classified by trained physicians to three groups: Responder group, Nonresponder group and Unclassified patients. Only the Responder and Nonresponder groups were selected for further analysis.

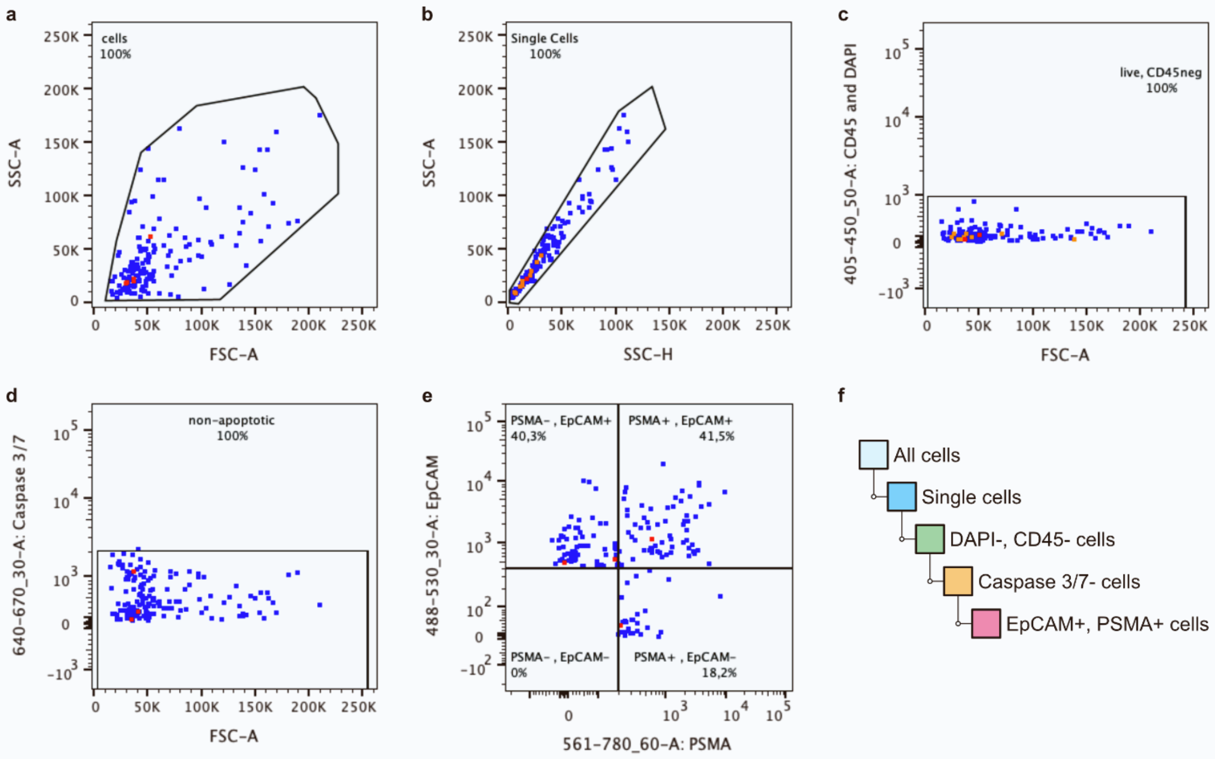

Figure S2. Gating strategy for CTC sorting. The data shown are concatenated from multiple indexed single-cell sorts and include only the cells that were successfully sorted. **(a)** The first step where, cells were identified while debris and aggregates were excluded. **(b)** Next, doublets were removed based on side scatter area (SSC-A) versus side scatter height (SSC-H) parameters. **(c)** Dead cells and CD45^{+} leukocytes were simultaneously excluded using a combined DAPI and CD45 staining gate. **(d)** To avoid sorting apoptotic cells, caspase-positive cells were excluded. **(e)** In the final step, three distinct populations of interest were identified based on PSMA and EpCAM expression. Only cells positive for at least one of these markers were sorted, while cells negative for both were excluded. **(f)** Gating hierarchy, with each population corresponding to the plots shown above.

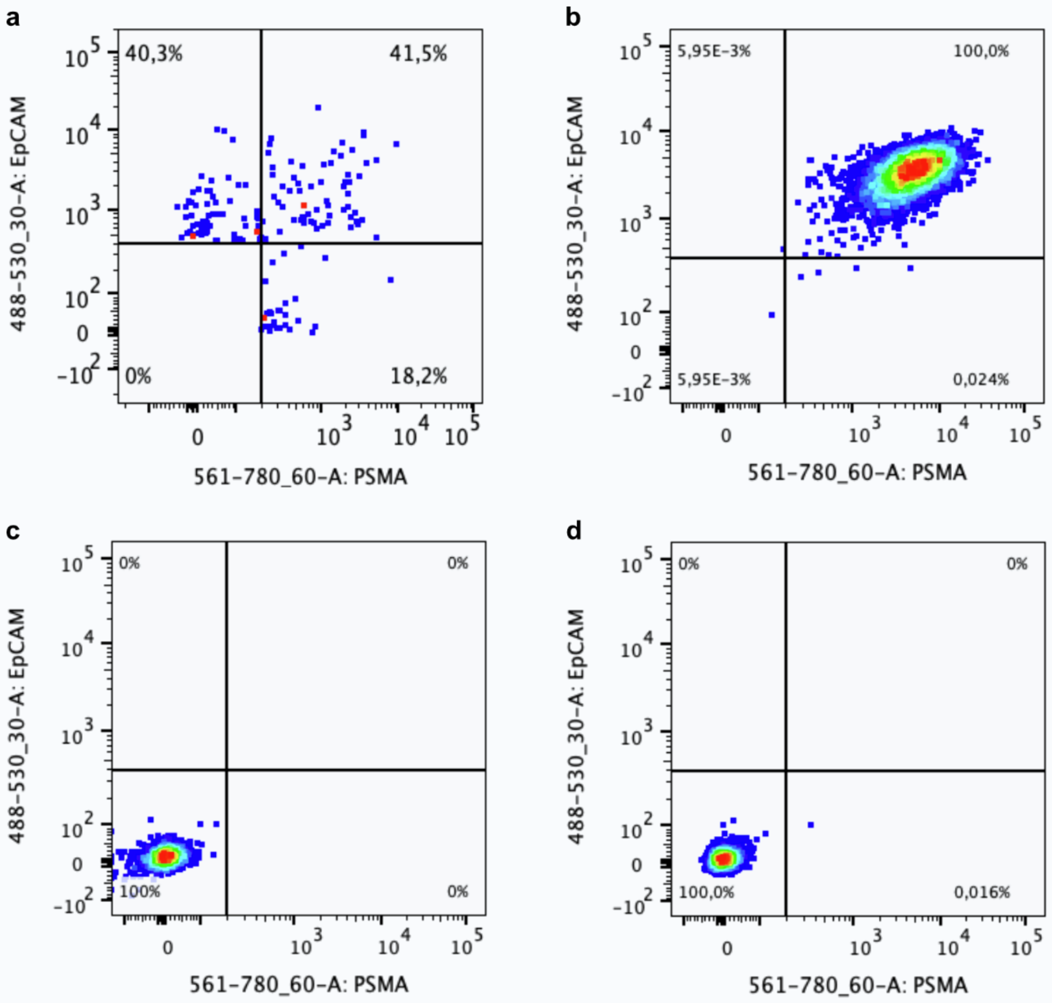

Figure S3. Controls used to establish accurate gating for CTC identification. (a) A panel showing concatenated data from multiple indexed single-cell sorts. All cells depicted were classified as circulating tumor cells (CTCs), as they expressed at least one of the markers PSMA or EpCAM. **(b)** The positive control, consisting of C4-2 prostate cancer cells, which are known to express both PSMA and EpCAM. **(c)** $CD45^+$ leukocytes that underwent the same staining protocol as the pre-enriched $CD45^-$ cell fraction; these cells were stained with the full antibody panel, including PSMA and EpCAM. **(d)** The negative control, which was not stained with any antibodies and served to assess background fluorescence.

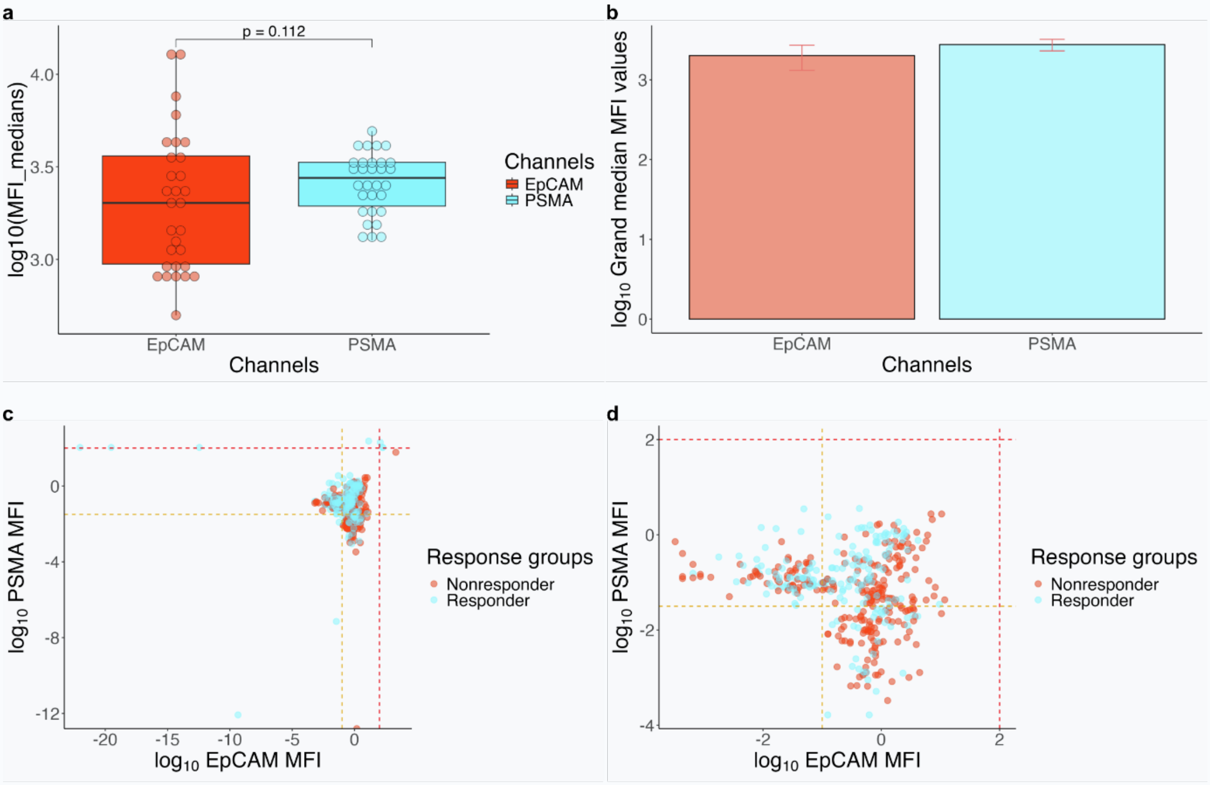

Figure S4. Control C4-2 surface marker levels and CTC filtering after FACS sorting. (a) A box plot showing the median EpCAM and PSMA MFI values on the sorted C4-2 control cells from multiple FACS sorts. The p-value (0.112) was calculated using the Mann-Whitney-U-test. **(b)** A box plot showing the grand median MFI values for EpCAM and PSMA on the control C4-2 cells, and the median absolute deviation of the medians (MADM). The grand median was calculated from multiple FACS experiments. **(c)** A scatter plot showing the CTC filtering strategy where cells with outlying EpCAM or PSMA values were excluded from the downstream analysis. **(d)** A scatter plot showing the distribution of sorted CTCs, which passed the filtering step.

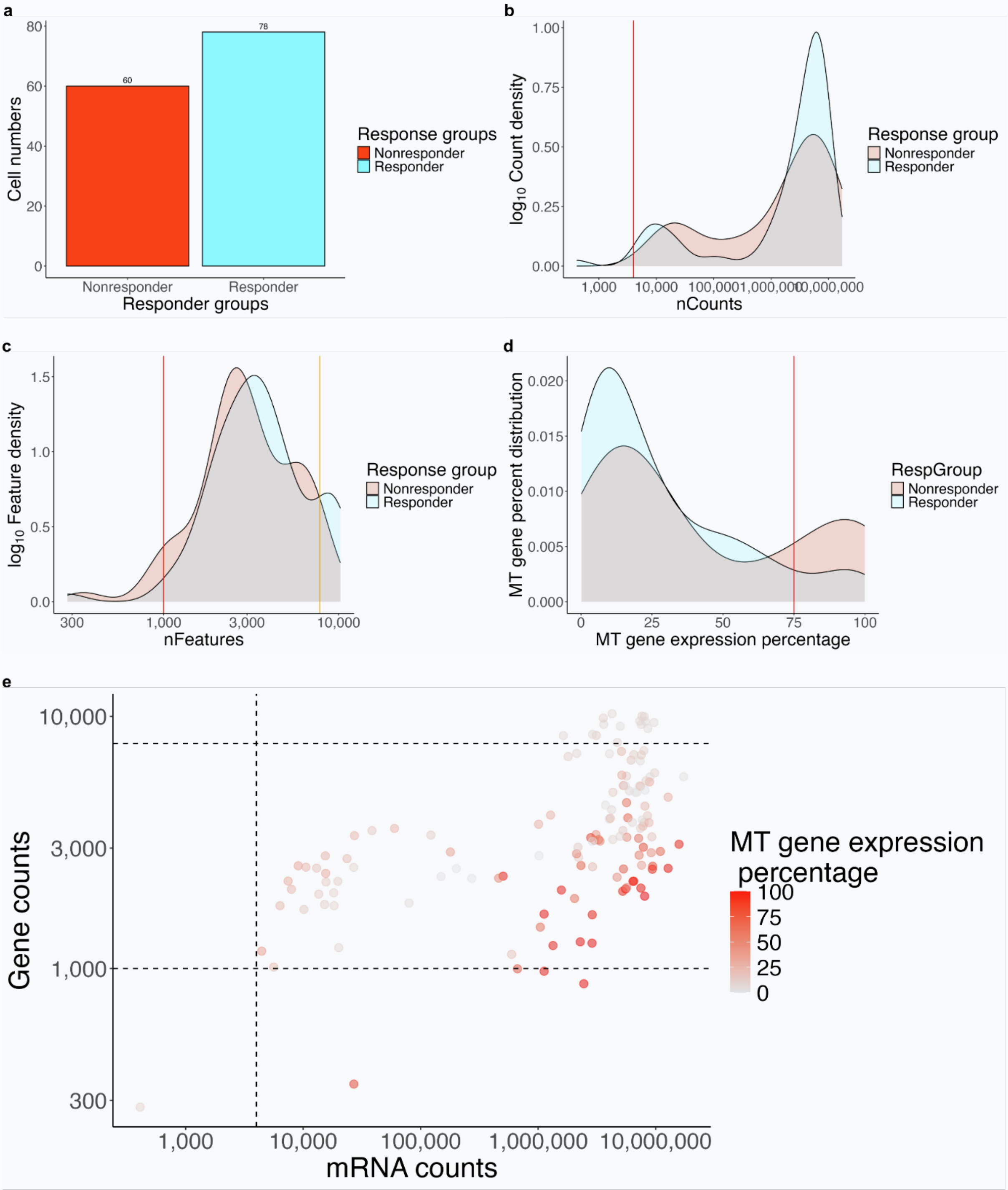

Figure S5. CTC filtering based on various metrics. (a) A bar chart showing the total number of sequenced CTCs from both Responder and Nonresponder samples before filtering. **(b)** A density plot showing the distribution of RNA counts obtained from the sequenced CTCs before filtering. The red vertical bar represents the minimum number of accepted counts for filtering. **(c)** A density plot showing the distribution of detected genes (Features) in the sequenced CTCs before filtering. The red vertical bar represents the minimum number of accepted genes, while the orange bar represents the maximum number of accepted genes for filtering. **(d)** A density plot showing the distribution of mitochondrial (MT) gene percentage obtained from the sequenced CTCs before filtering. The red vertical bar represents the maximum accepted MT gene percentage for filtering. **(e)** A scatter plot showing the distribution of the sequenced CTCs based on RNA and gene counts, and colored by their MT gene expression percentage before filtering. The dotted lines represent the various RNA and gene count thresholds used for filtering.

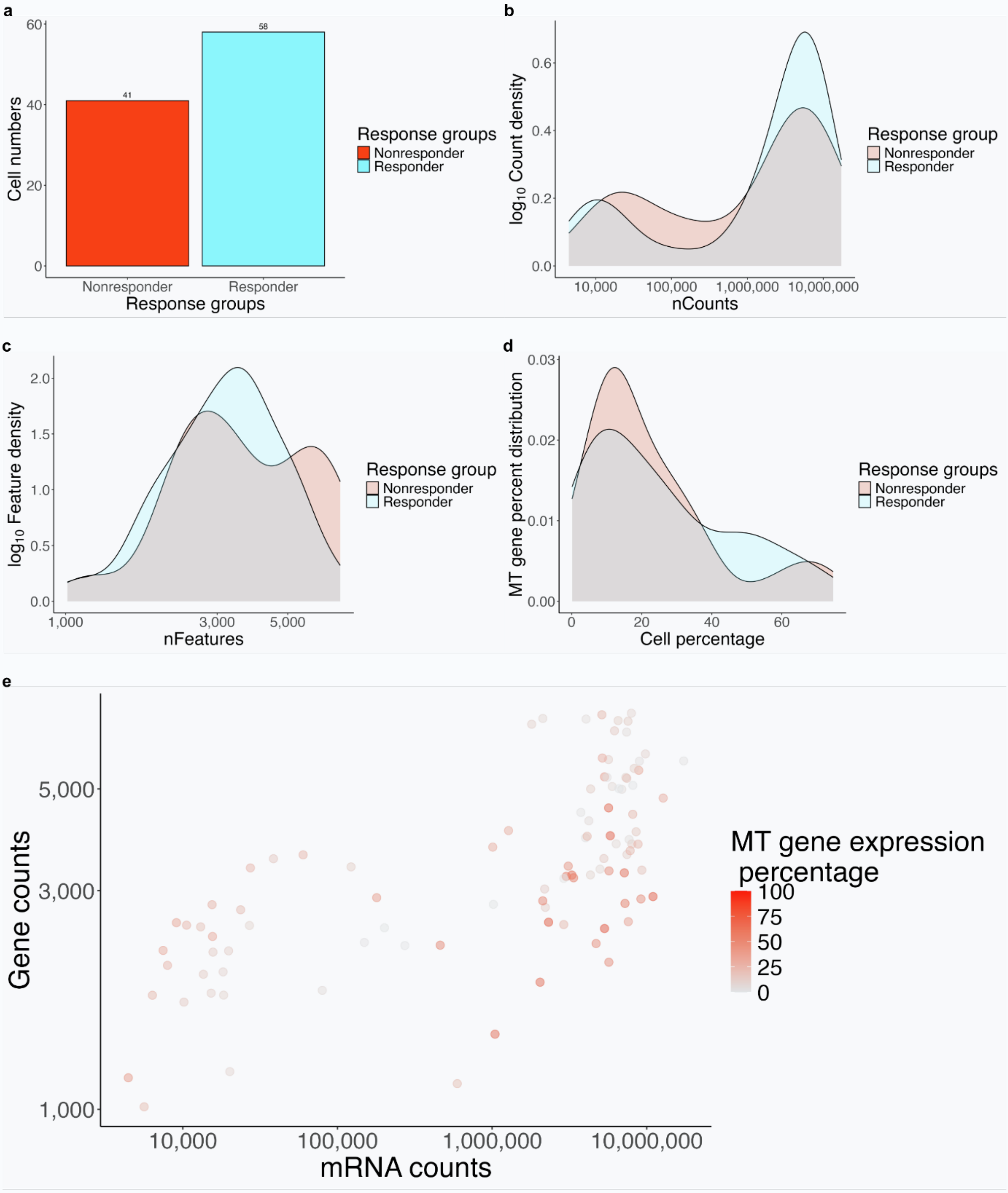

Figure S6. Various CTC metrics after filtering. (a) A bar chart showing the total number of sequenced CTCs from both Responder and Nonresponder samples after filtering. **(b)** A density plot showing the distribution of RNA counts in the filtered CTCs. **(c)** A density plot showing the distribution of detected genes (Features) in the filtered CTCs. **(d)** A density plot showing the distribution of mitochondrial (MT) gene percentages in the filtered CTCs. **(e)** A scatter plot showing the distribution of the filtered CTCs based on RNA and gene counts, and colored by their MT gene expression percentage.

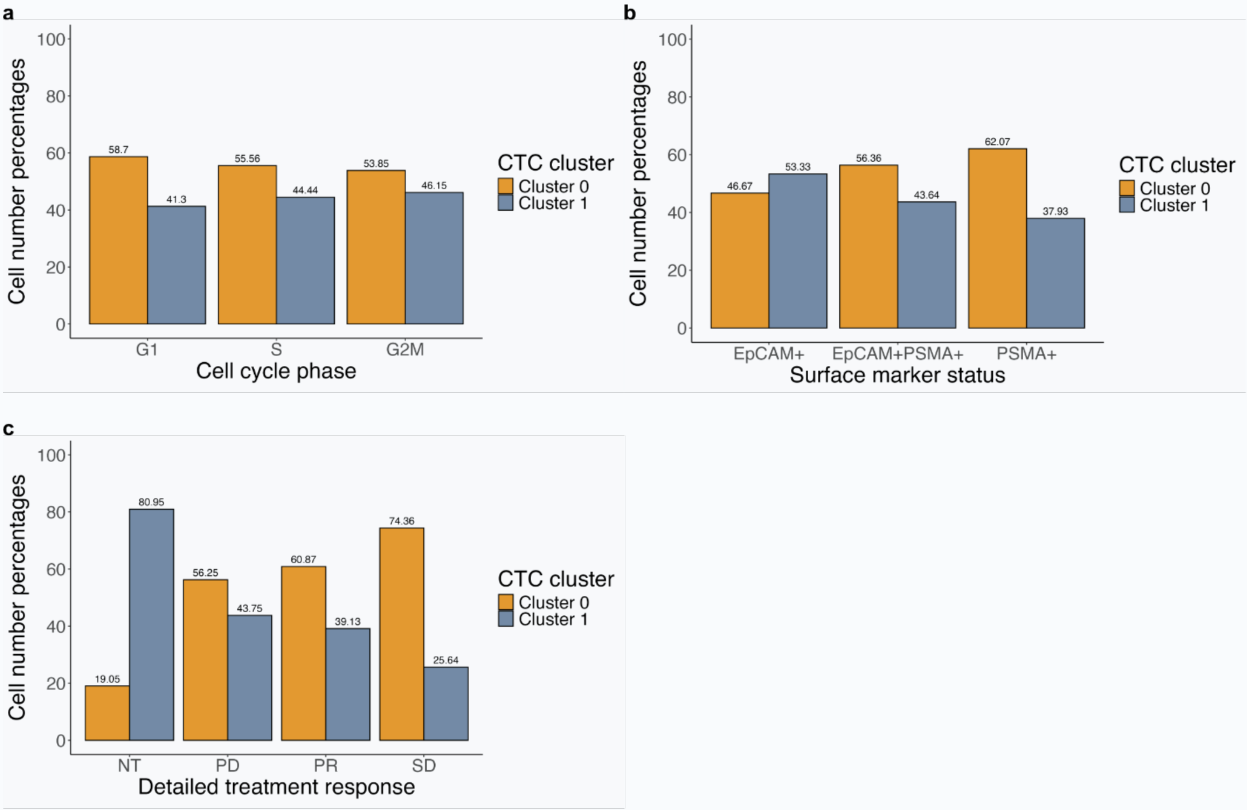

Figure S7. CTC clusters and their correlation to various treatment variables. (a) A bar chart showing the distribution of CTCs between clusters 0 and 1, based on their cell cycle phase. **(b)** A bar chart showing the distribution of CTCs between clusters 0 and 1, based on their surface marker status. **(c)** A bar chart showing the distribution of CTCs between clusters 0 and 1, based on detailed patient response status. NT = not treated, PD = progressive disease, PR = partial response, SD = stable disease.

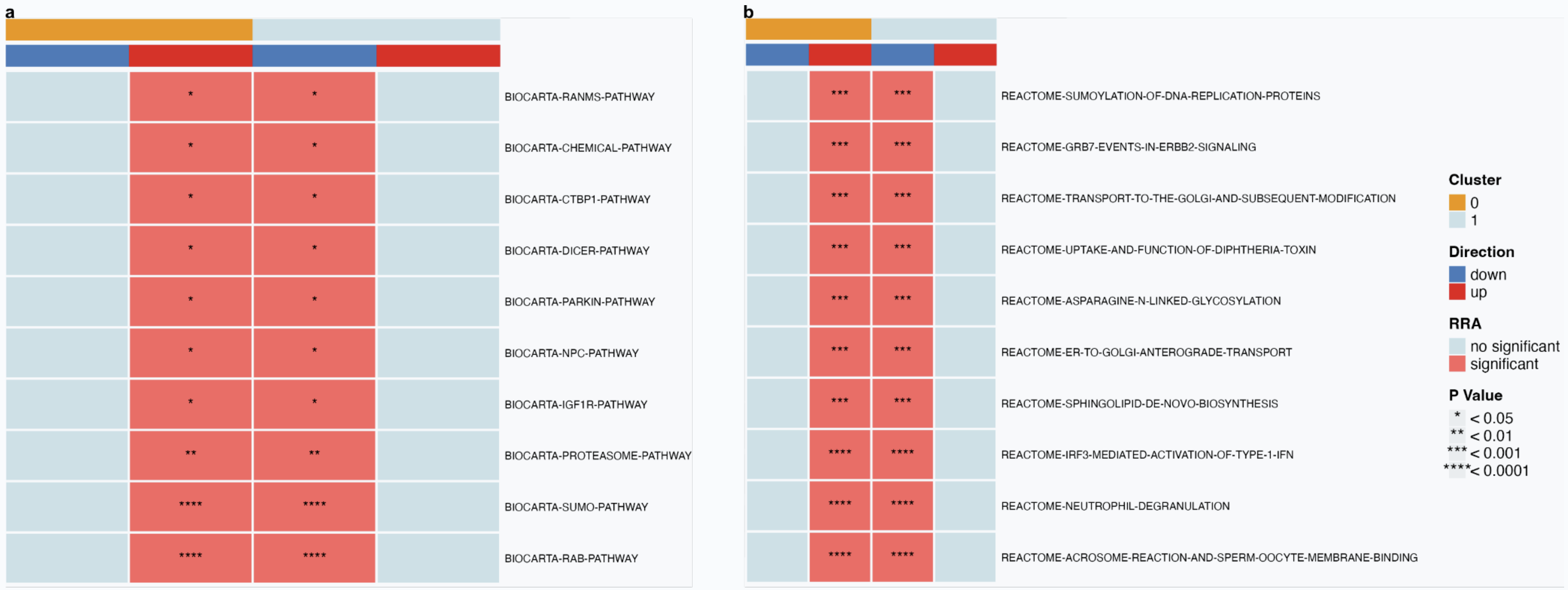

Figure S8. Differentially regulated pathways among the CTC clusters found by irGSEA. **(a)** A heat map showing the 10 most significantly impacted pathways based on the BIOCARTA gene sets. **(b)** A heat map showing the 10 most significantly impacted pathways based on the REACTOME gene sets.

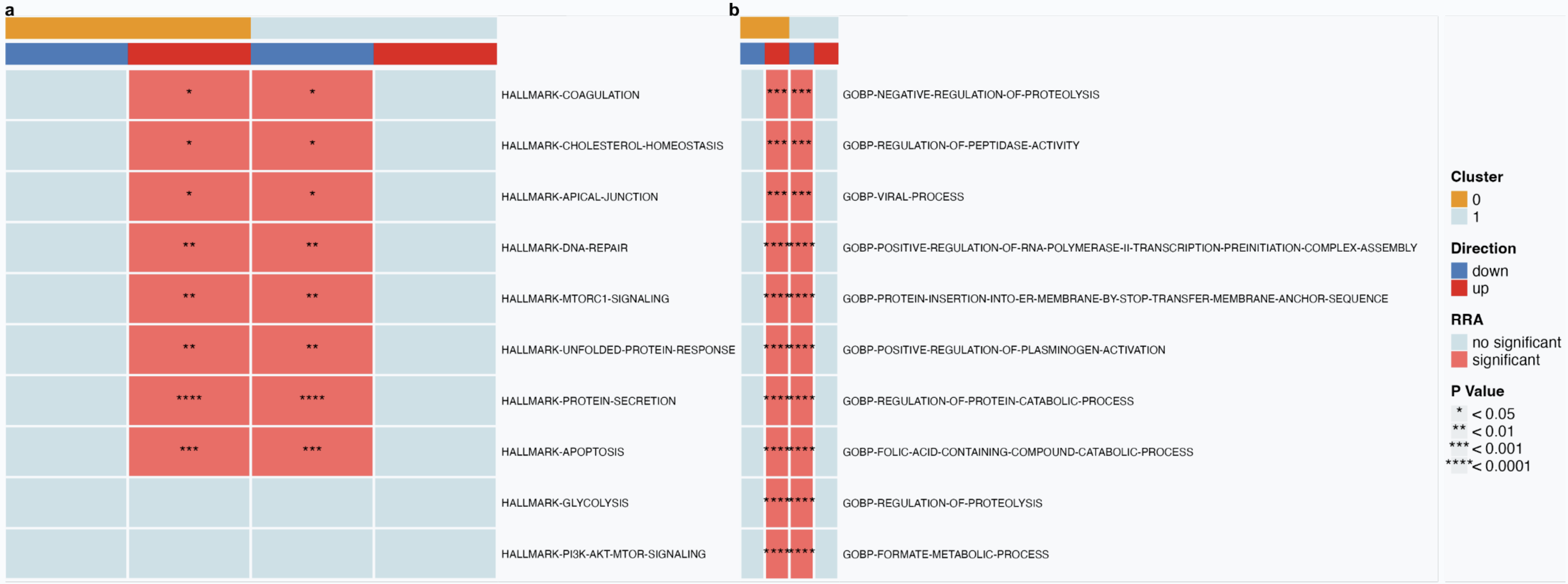

Figure S9. Differentially regulated pathways among the CTC clusters found by irGSEA. (a) A heat map showing the 10 most significantly impacted pathways based on the HALLMARK gene sets. **(b)** A heat map showing the 10 most significantly impacted pathways based on the GENE ONTOLOGY: BIOLOGICAL PROCESSES (GO:BP) gene sets.

Supplemental Tables

Table S1. PSA values over the treatment cycles. A table showing changes in PSA values for patients whose CTC samples were successfully sequenced and included in the final scRNA-seq analysis during a combined [225Ac]Ac-PSMA-617 - [177Lu]Lu-PSMA-617 treatment.

Patient code	Baseline PSA (ng/ml)	Follow up 1 (FU1) (ng/ml)	FU2 (ng/ml)	FU3 (ng/ml)	FU4 (ng/ml)	FU5 (ng/ml)	Status at each cycle	Final Response status
ALM60C8002	190	174	80,32	47,39	20,82	15,39	1.-5. PR	Responder
ALM63C9005	328,2	210,9					1. SD	Nonresponder
ALM69C5003	56,17	84,42	105,75				1. SD; 2 PD	Nonresponder
ALM70C8006	688,65	443,74					1. SD; 2 PD	Nonresponder
ALM74C8008	4,7	3,13	1,01				1-2 PR	Responder
ALM76C6003	111,27	373,09					1. PD	Nonresponder
ALM77C5004	68,9	13,35	8,19				1. SD; 2. PR	Responder
ALM77C5005	38,98	17,88	5,36				1. SD; 2. PR	Responder
ALM78C7005	332,8	248,7	83,8	26,2	8,9		1-4 PR	Responder
ALM79C9003	42,7	111,29					1. PD	Nonresponder
ALM80C6007	823,69	633,97	251	480,47			1. SD; 2. SD; 3. SD	Nonresponder
ALM80C7001	119,56	4,34	0,17				1.-2. PR	Responder
ALM80C8004	137,69	66,96	30,5	15,34			1. PR; 2. PR; 3. PR	Responder
ALM81C2001	308,6	48,84	16,39	5,11			1. PR; 2. PR; 3. PR	Responder
ALM82C6002	88,21	26.95					1. PR; 2. PR	Responder
ALM82C7004	12,77	19,5	47,8				1. SD; 2. PD	Nonresponder
ALM83C8001	0,5	0,33	<0,1				1.-2. PR	Responder
ALM88C3002	846	464,25	1684,57				1. SD; 2. PD	Nonresponder
LM66C3003	6,4	4,21	5,58	12,32	13,3	23,8	1-5. SD	Nonresponder
LM67C6011	584	99,4	28,2	4,6			1-3. PR	Responder
LM68C6005	715	578,82	537,08	358,53			1. PR; 2. SD; 3. PR	Responder
LM75C7006	153,2	80,5	41,7	22,5			1-3. PR	Responder
LM81C6017	82,9	15,5	186,9	378			1. PR ; 2. PD; 3. PD	Nonresponder

Table S2. Differentially Expressed Genes (DEG) table. A table showing the significant (p_adjusted_value < 0,05; Bonferroni-correction) DEGs between Cluster 0 and Cluster 1 based on the Wilcoxon Rank Sum test.

Table S3. irGSEA hub gene analysis table. This table presents the hub genes whose expression significantly differs between Cluster 0 and Cluster 1. It includes the genes, their associated MSigDB database entries, the pathways they impact, and relevant references provided in the Supplemental Materials.

ENSEMBL_ID	Gene_symbol	General_expression_in_PCa	DEG_staus-Cluster0_vs_1	References	MSigDB	Pathway
ENSG00000015475	BID	Downregulated	Upregulated	[4, 5]	BIOCARTA	Chemical_pathway
ENSG00000100401	RANGAP1	-	Upregulated	[6]	BIOCARTA	Nuclear_Pore_Complex_pathway
ENSG00000103769	RAB11A	Upregulated	Upregulated	[7, 8]	BIOCARTA	RAB_pathway
ENSG00000116030	SUMO1	Upregulated	Upregulated	[9, 10, 11, 12, 13, 14]	BIOCARTA	SUMO_pathway and CTBP1_pathway
ENSG00000121879	PIK3CA	Upregulated	Downregulated	[15, 16, 17]	BIOCARTA	IGFR1R_signaling
ENSG00000123595	RAB9A	-	Upregulated	[7]	BIOCARTA	RAB_pathway
ENSG00000126261	UBA2	-	Upregulated	[9, 11]	BIOCARTA	SUMO_pathway
ENSG00000140443	IGF1R	Upregulated	Upregulated	[18, 19]	BIOCARTA	IGFR1R_signaling
ENSG00000144566	RAB5A	Downregulated	Downregulated	[7, 20]	BIOCARTA	RAB_pathway
ENSG00000165806	CASP7	Downregulated	Upregulated	[21, 22]	BIOCARTA	Chemical_pathway
ENSG00000168118	RAB4A	Downregulated	Upregulated	[7, 20]	BIOCARTA	RAB_pathway
ENSG00000188612	SUMO2	-	Upregulated	[9, 23, 11]	BIOCARTA	SUMO_pathway
ENSG00000074800	ENO1	Upregulated	Upregulated	[24]	GOBP	Plasminogen_activation
ENSG00000105707	HPN	Upregulated	Downregulated	[25, 26]	GOBP	Plasminogen_activation
ENSG00000110330	BIRC2	-	Upregulated	[27, 28, 29]	GOBP	K63-linked_ubiquitination
ENSG00000111530	CAND1	Upregulated	Upregulated	[30]	GOBP	RNA-POL-II_preinit._complex_assembly
ENSG00000141510	TP53	Upregulated	Upregulated	[31, 32, 33]	GOBP	RNA-POL-II_preinit._complex_assembly
ENSG00000169139	UBE2V2	Upregulated	Upregulated	[34, 35, 36, 37, 38]	GOBP	K63-linked_ubiquitination
ENSG00000177889	UBE2N	Upregulated	Upregulated	[34, 35, 36]	GOBP	K63-linked_ubiquitination
ENSG00000244687	UBE2V1	-	Upregulated	[34, 35, 36]	GOBP	K63-linked_ubiquitination
ENSG00000152669	CCNO	-	Upregulated	[39, 40, 41, 42]	HALLMARK	DNA_repair
ENSG00000184432	COPB2	Upregulated	Upregulated	[43, 44, 45]	HALLMARK	protein_secretion
ENSG00000186141	POLR3C	Upregulated	Upregulated	[46, 47]	HALLMARK	DNA_repair
ENSG00000205302	SNX2	Upregulated	Upregulated	[48, 49, 50]	HALLMARK	protein_secretion
ENSG00000087586	AURKA	Upregulated	Upregulated	[51, 52, 53, 54, 55]	HALLMARK and REACTOME	mTROC1_signaling and Sumoylation_of_DNA-replication_proteins
ENSG00000065361	ERBB3	Upregulated	Downregulated	[56, 57, 58, 59]	REACTOME	GRB7_events_in_ERBB2_signaling
ENSG00000134690	CDCA8	Upregulated	Upregulated	[60, 61, 62, 55, 63]	REACTOME	Sumoylation_of_DNA-replication_proteins
ENSG00000141736	ERBB2	Upregulated	Upregulated	[64, 65, 66]	REACTOME	GRB7_events_in_ERBB2_signaling
ENSG00000196655	TRAPPC4	-	Upregulated	[67, 68]	REACTOME	ER_to_golgi_transsport
ENSG00000198431	TXNRD1	Upregulated	Upregulated	[69, 70]	REACTOME	Diphtheria_toxin_signaling

Table S4. Index primers. A table showing the i5 - i7 index primer pairs and their sequences used during library preparation.

Table S5. Optical configuration table. A table showing the optical configuration of the BD FACSAria Fusion Flow Cytometer used for single-cell sorting.

Laser (nM)	Channel Name-Collection Filter	LP	Fluorochromes (Some)
**355 UV**	450/50	-	DAPI, DyLight 350
	525/50	505	BUV496
405 **Violet**	405-450/50	-	DAPI, Pacific Blue, AF 405, BV421, v450, BFP, CFP, CellTrace Violet
	405-525/50	505	Sytox Blue, L/D Fix Aqua, AmCyan, v500, AF 430, Pacific Orange, Qdot 525
	405-610/20	595	BV605, Qdot 605, eFluor 605NC, L/D Fix Yellow, Pacific Orange
	405-660/20	630	BV650, Qdot 655
	405-710/50	690	BV711
	405-780/60	750	BV786
488 **Blue**	488-530/30	502	FITC, GFP, YFP, Alexa 488, mCitrine, Syto9, Sytox Green, Calcein
	488-710/50	690	PerCP-Cy5.5, DRAQ5, PerCP, efluor710
561 **Yellow-Green**	561-586/15	-	PE, DsRed, RFP, tdTomato, Sytox Orange
	561-610/20	600	PI, mCherry, Texas Red, PE-CF594
	561-670/14	630	7-AAD, PE-Cy5
	561-710/50	685	PE-Cy5.5, PE-Cy5, 7-AAD
	561-780/60	735	PE-Cy7, APC-Vio770, PE-Vio 770
633 **Red**	640-670/30	-	APC, AF 647, DRAQ5, DRAQ7, L/D Fix Far Red, TO-Pro3
	640-730/45	690	AF 700, APC-CY5.5, DRAQ7, Dye Cycle Ruby
	640-780/60	755	APC-Cy7, AF 750, APC-eFluor 780, APC-H7, L/D Fix near-IR

Table S6. Patient treatment history. A table showing the pre-treatment history of patients whose whose CTC samples were successfully sequenced and included in the final scRNA-seq analysis during a combined [225Ac]Ac-PSMA-617 - [177Lu]Lu-PSMA-617 treatment.

Patient code	PSMA-RLT pretreatment	Chemoterapy received	Taxanes received	Platins received	Docetaxel received	Cabazitaxel received	Etoposide received	Xofigo received	ADT received	ADT	Denosumab received	Immunotherapy received	External Adjuvant Radiation received	Palliative External Radiation received	BRCA status	PARP inhibitor received	Surgery (total prostatectomy)
ALM60C8002	No	Yes	Yes	No	Yes	No	No	No	Yes	Leuprorelin acetate, Goserelin Acetate, Bicalutamide	Yes	NA	Yes	Yes	Negative	No	No
ALM63C9005	No	Yes	Yes	No	Yes	No	No	No	Yes	Bicalutamide, Goserelin Acetate, Leuprorelin, Abiraterone	No	NA	Yes	No	Negative	No	Yes
ALM69C5003	No	Yes	Yes	Yes	Yes	No	Yes	No	Yes	Enzalutamide	Yes	NA	Yes	Yes	Postive	Yes	Yes
ALM70C8006	No	Yes	Yes	No	Yes	No	No	No	Yes	Enzalutamide, Abiraterone	No	NA	Yes	No	Negative	Yes	No
ALM74C8008	No	No	No	No	No	No	No	No	Yes	Enzalutamide	Yes	NA	Yes	Yes	Negative	No	No
ALM76C6003	Yes	Yes	Yes	No	Yes	Yes	No	No	Yes	Enzalutamide, Abiraterone, Bicalutamid	No	NA	No	Yes	Negative	No	No
ALM77C5004	no	Yes	Yes	No	Yes	Yes	No	No	Yes	Enzalutamide	Yes	NA	Yes	No	Unknown	Yes	Yes
ALM77C5005	Yes	Yes	Yes	No	Yes	Yes	No	No	Yes	Enzalutamide, Abiraterone	Yes	NA	No	Yes	Negative	No	No
ALM78C7005	Yes	Yes	Yes	No	Yes	No	No	No	Yes	Abiraterone, Goserelin Acetate	No	NA	yes	yes	Unknown	No	No
ALM79C9003	Yes	Yes	Yes	No	Yes	Yes	No	No	Yes	Enzalutamide, Abiraterone, Leuprorelin acetate,	Yes	NA	No	No	Negative	No	No
ALM80C6007	Yes	Yes	Yes	No	Yes	Yes	No	No	Yes	Enzalutamide	No	NA	No	Yes	Negative	No	No
ALM80C7001	Yes	No	No	No	No	No	No	No	Yes	Enzalutamide, Bicalutamid	No	NA	No	No	Unknown	No	No
ALM80C8004	Yes	Yes	Yes	Yes	Yes	Yes	No	No	Yes	Enzalutamide, Abiraterone	No	NA	No	Yes	Negative	No	No
ALM81C2001	Yes	No	No	No	No	No	No	No	Yes	Bicalutamide, Buserelin Acetate, Abiraterone	No	NA	Yes	No	Unknown	No	No
ALM82C6002	Yes	Yes	Yes	No	Yes	Yes	No	No	Yes	Bicalutamide, Leuprorelin acetate, Flutamide	NA	No	Yes	Yes	Unknown	No	Yes
ALM82C7004	Yes	Yes	Yes	No	Yes	No	No	No	Yes	Unknown	NA	No	Yes	Yes	Unknown	No	Yes
ALM83C8001	No	Yes	Yes	Yes	Yes	No	No	No	Yes	Unknown	NA	No	No	Yes	Unknown	No	Yes
ALM88C3002	No	Yes	Yes	No	Yes	No	No	No	Yes	Unknown	NA	No	Yes	Yes	Unknown	No	Yes
LM66C3003	No	No	No	No	No	No	No	No	Yes	Unknown	NA	No	No	No	Unknown	No	Yes
LM67C6011	No	No	No	No	No	No	No	No	Yes	Unknown	NA	No	No	No	Unknown	No	No
LM68C6005	No	Yes	Yes	No	Yes	Yes	No	Yes	Yes	Unknown	NA	No	No	Yes	Unknown	No	No
LM75C7006	No	No	No	No	No	No	No	No	Yes	Unknown	NA	No	No	No	Unknown	Yes	No
LM81C6017	No	Yes	Yes	No	Yes	No	No	No	Yes	Unknown	NA	No	Yes	No	Unknown	No	No

Table S7. Primer sequence table. A table showing the sequences for the primers used for Tn5 based tagmentation.

Primer name	Sequence
**TSO**	5′-AAGCAGTGGTATCAACGCAGAGTACATrGrG+G-3′
**Tn5ME-A**	5′-TCGTCGGCAGCGTCAGATGTGTATAAGAGACAG-3′
**Tn5ME-B**	5′-GTCTCGTGGGCTCGGAGATGTGTATAAGAGACAG-3′
**Tn5RMErev**	5′-[phos]CTGTCTCTTATACACATCT-3′

Supplemental Methods

Single-cell RNA sequencing data analysis – read alignment and filtering

<u>Data filtering:</u>

We opted to be more lenient with the chosen MT gene expression cutoff for three main reasons:

a. We used both live-dead and apoptotic staining during cell sorting, which should ensure that dead and apoptotic cells were excluded from sequencing.
b. Ionizing radiation can affect the expression of various mitochondrial genes and metabolic processes, which might have an impact on radiation resistance [1,2].
c. Smart-Seq2 tends to generate a considerably higher proportion of mitochondrial reads than similar methods, such as 10X Genomics. This is most likely due to the more thorough organelle membrane disruption during the library preparation process [3]. This might be amplified in our case, as we used a higher-than-standard concentration of Triton X-100, to ensure proper cell lysis.

Supplemental References

1. Chaudhry, M.A., and Omaruddin, R.A. (2011). Mitochondrial gene expression in directly irradiated and nonirradiated bystander cells. Cancer Biother Radiopharm *26*, 657-663. 10.1089/cbr.2010.0940.
2. Kostyuk, S.V., Proskurnina, E.V., Konkova, M.S., Abramova, M.S., Kalianov, A.A., Ershova, E.S., Izhevskaya, V.L., Kutsev, S.I., and Veiko, N.N. (2021). Effect of Low-Dose Ionizing Radiation on the Expression of Mitochondria-Related Genes in Human Mesenchymal Stem Cells. Int J Mol Sci *23*. 10.3390/ijms23010261.
3. Wang, X., He, Y., Zhang, Q., Ren, X., and Zhang, Z. (2021). Direct Comparative Analyses of 10X Genomics Chromium and Smart-seq2. Genomics Proteomics Bioinformatics *19*, 253-266. 10.1016/j.gpb.2020.02.005.
